# Regiospecific Synthesis of Ring A Fused Withaferin A Isoxazoline Analogues: Induction of Premature Senescence by W-2b in Proliferating Cancer Cells

**DOI:** 10.1038/s41598-017-13664-x

**Published:** 2017-10-23

**Authors:** Faheem Rasool, Debasis Nayak, Archana Katoch, Mir Mohd Faheem, Syed Khalid Yousuf, Nazar Hussain, Chetan Belawal, N. K. Satti, Anindya Goswami, Debaraj Mukherjee

**Affiliations:** 1 Academy of Scientific and Innovative Research (AcSIR), New Delhi, India; 2 Natural Product Chemistry-Microbes, CSIR-Indian Institute of Integrative Medicine, Jammu, 180001 India; 3 Cancer Pharmacology Division, CSIR-Indian Institute of Integrative Medicine, Jammu, 180001 India; 4 Medicinal Chemistry Division, CSIR-Indian Institute of Integrative Medicine, Srinagar, 190005 India; 5 Natural Product Chemistry-Plants, CSIR-Indian Institute of Integrative Medicine, Jammu, 180001 India

## Abstract

Induction of premature senescence represents a novel functional strategy to curb the uncontrolled proliferation of malignant cancer cells. This study unveils the regiospecific synthesis of novel isoxazoline derivatives condensed to ring A of medicinal plant product Withaferin-A. Intriguingly, the *cis* fused products with β-oriented hydrogen exhibited excellent cytotoxic activities against proliferating human breast cancer MCF7 and colorectal cancer HCT-116 cells. The most potent derivative **W-2b** triggered premature senescence along with increase in senescence-associated β-galactosidase activity, G2/M cell cycle arrest, and induction of senescence-specific marker p21^Waf1/Cip1^ at its sub-toxic concentration. **W-2b** conferred a robust increase in phosphorylation of mammalian checkpoint kinase-2 (Chk2) in cancer cells in a dose-dependent manner. Silencing of endogenous Chk2 by siRNA divulged that the amplification of p21 expression and senescence by **W-2b** was Chk2-dependent. Chk2 activation (either by ectopic overexpression or through treatment with **W-2b**) suppressed NM23-H1 signaling axis involved in cancer cell proliferation. Finally, **W-2b** showed excellent *in vivo* efficacy with 83.8% inhibition of tumor growth at a dose of 25 mg/kg, b.w. in mouse mammary carcinoma model. Our study claims that **W-2b** could be a potential candidate to limit aberrant cellular proliferation rendering promising improvement in the treatment regime in cancer patients.

## Introduction

Natural products, particularly steroids, have been employed as a powerful tool for deciphering new biological targets^1,2^. In the last two decades, the search for biologically active steroids has led to the successful development of emerging heterocyclic steroid derivatives^3,4^. The main driving force towards the preparation of such compounds primarily confers upon the modification of the receptor-binding ability by chemical transformation of the extant functional groups for the reduction or elimination of the undesirable side effects and also modulation of pharmacodynamic and pharmacokinetic properties^5^. Indeed, transforming parent bioactive natural steroids to more/new bioactive ones via semisynthetic approach has enlightened researchers for paving way of drug development.

Withaferin A (WA) is a naturally occurring steroidal lactone, the first member of the withanolide class of compounds derived from the medicinal plant *Withania somnifera*, commonly known as Ashwagandha or Indian winter cherry^6^. The presence of the steroidal framework has endowed WA with antiangiogenic properties. Its tremendous potential to modulate various oncogenes and tumor-suppressor genes with appreciable *in vivo* activities, bioavailability and less toxicity have conferred the molecule a suitable anticancer candidate^7,8^. Among the five-membered heterocyclic compounds, 2-isoxazolines have gained tremendous attention from the medicinal chemists as structural building blocks of biologically active molecules and versatile intermediates in organic synthesis^9^. The importance of isoxazolines also stem from their utility as precursors in the synthesis of 1,3-aminoalcohols, which are excellent starting materials for a wide variety of natural products and related compounds such as alkaloids and nucleoside antibiotics^10^. Thus, the isoxazoline ring system could be semi-synthetically manipulated in presence of bioactive natural product WA for the discovery of novel leads with anticancer therapeutic potential.

Cellular senescence is regarded as a safeguard mechanism to protect the organism by preventing uncontrolled proliferation of malignant cancer cells^11^. Senescent cells possess characteristic features including growth arrest, flattened cellular morphology, SA-β-gal activity, and augmentation of cell-cycle specific marker such as cyclin-dependent kinase inhibitor p21^12^. Premature senescence occurs in response to various exogenous and endogenous insults including DNA damage and reactive oxygen species (ROS) generation etc., which is independent of telomere length and number of replications^13^. Checkpoint kinase 2 (Chk2) is a universal tumor suppressor gene that is activated in response to various genotoxic threats including ionizing radiation (IR) or chemotherapies^14^. DNA double-strand breaks (DSBs) activate ataxia telangiectasia mutated (ATM) protein kinase that phosphorylates Chk2 at Thr68 and activates it^15^. The ATM and Chk2 act in a linear fashion to stabilize tumor suppressor p53 leading to either cell-cycle arrest or apoptosis^15^. Chk2 is an essential component to induce both replicative and premature senescence through cell-cycle arrest by activating p21 in a p53 dependent manner^16^. However, studies also found that Chk2 can activate senescence in cancer cells by inducing p21, independent of the p53 status of the cell^17,18^. Hence, Chk2 is a lucratic target that can be manipulated to promote senescence in proliferating cancer cells.

Though therapeutic agents and small molecule natural products such as doxorubicin, camptothecin, resveratrol, triptolide etc., are reported to induce senescence by augmenting p21 through various mechanisms in human cancer cells^19,20^, the effect of WA and its derivatives on induction of premature senescence is yet to be examined. In this endeavour, we sought to examine the potential of fused 2-isoxazoline derivatives of WA to induce cytotoxicity in human cancer cells by abrogating cell proliferation through the induction of premature senescence.

## Results

### Chemistry of Withaferin A isoxazolines

**Figure 1:**
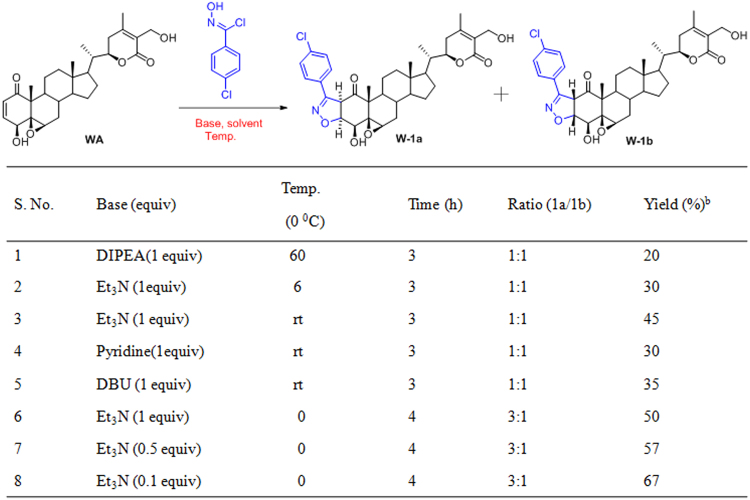
Standardization of the reaction conditions for the cis-fused isoxazoline derivatives of WA.^a^ ^a^In all cases WA (1 equiv), N-hydroxy-4-chlorobenzenecarboximidoyl chloride (1.2 equiv) in DMF was taken. ^b^Isolated yield after column chromatography.

Although there are plenty of reports available in the literature for 1,3-dipolar cycloaddition of nitrile oxide with alkenes^21^, but there are limited reports of it when the alkene is a part of internal α,β-unsaturated cyclic system^22^. Fused 2-isoxazoline derivatives of WA were prepared by reacting WA with aromatic hydroximidoyl chlorides (precursors of nitrile oxides), obtained from the corresponding aromatic aldehydes via two steps. We initiated our optimization study by taking WA and N-hydroxy-4-chlorobenzenecarboximidoyl chloride in DMF as summarized in Fig. 1. Initially, two stereoisomeric isoxazoline products namely **W-1a**, **W-1b** were obtained in lower yield (20%) using diisopropylethylamine (DIPEA) as base (Fig. 1, entry 1) at elevated temperature. In order to increase the yield of the reaction, we explored various bases in different proportion to find that triethylamine was the most appropriate for this cycloaddition (Fig. 1, entries 1–7). Again triethylamine in catalytic amount (10 mol %) was more effective than stoichiomeric amount (Fig. 1, entry 8). Temperature played a vital role in obtaining one stereoisomer over other as major product, such as by decreasing the temperature from rt to 0 °C, stereoisomer **W-1b**, in which both H2 and H3 are β was obtained in major quantity (entries 5–7). Thus, from the optimization study we concluded that WA (1 equiv), triethylamine (0.1 equiv), aromatic hydroximidoyl chloride (1.2 equiv) in DMF at 0 °C for 3 h was the optimal reaction condition for this cycloaddition reaction.

**Figure 2:**
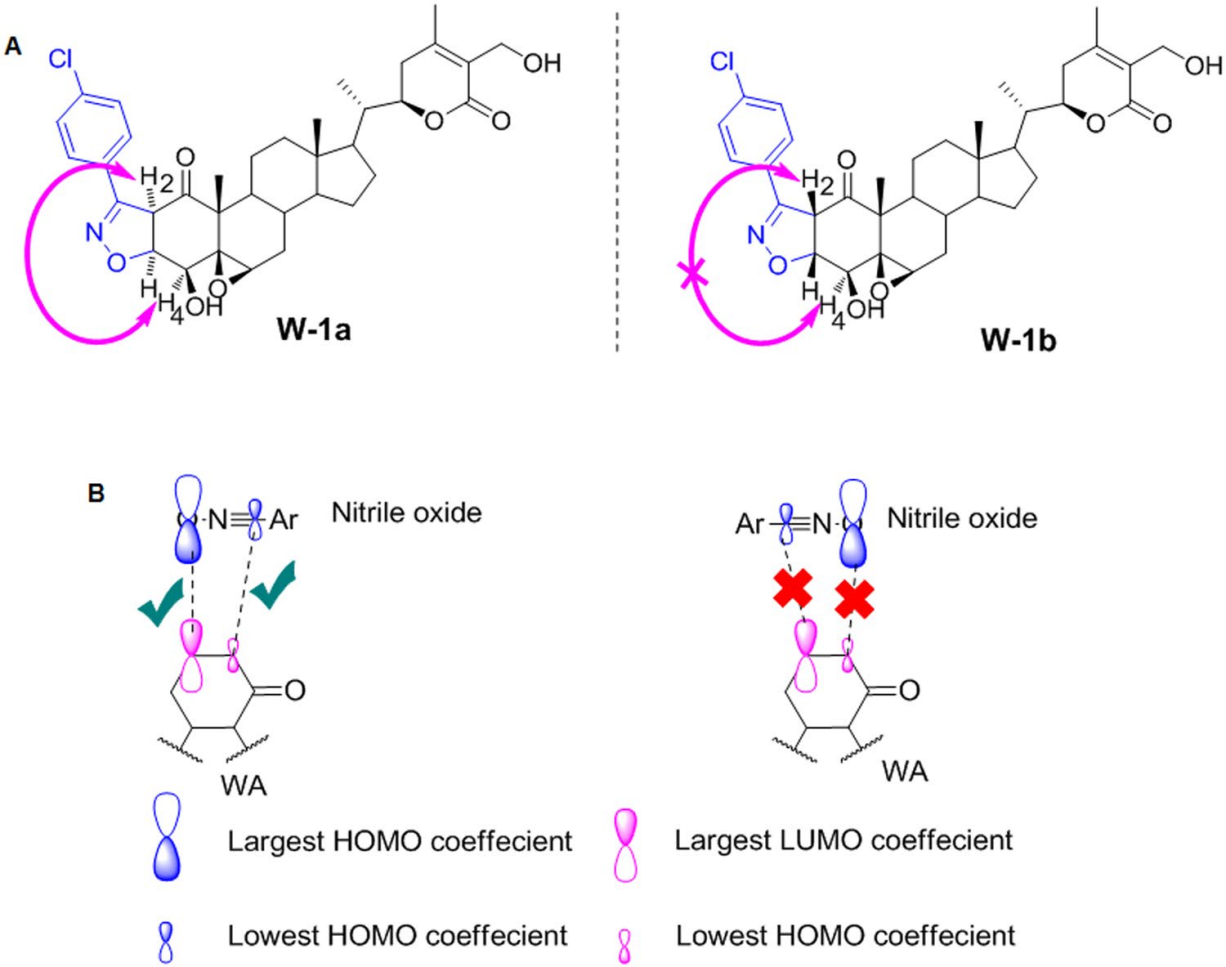
(**A**) NOESY of W-1a and W-1b. (**B**) Regioselectivity in 1,3-dipolar cycloaddition reaction of aromatic nitrile oxides with α,β-unsaturated ketone of ring A of WA.

**Figure 3:**
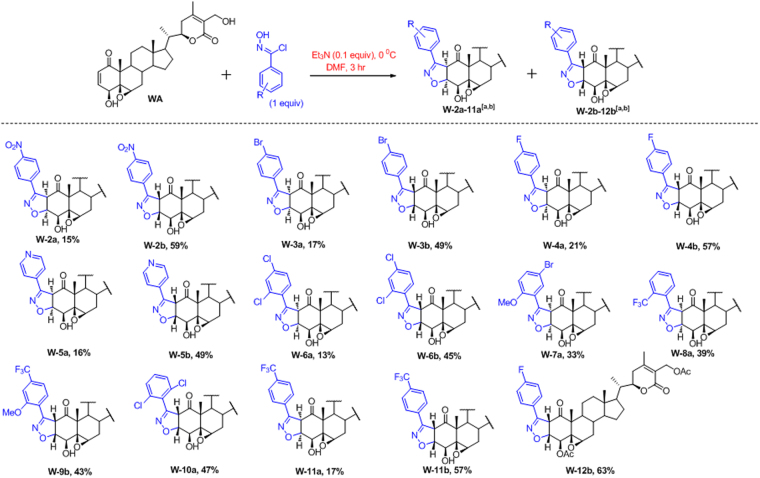
Reaction of WA with various N-hydroxy benzenecarboximidoyl chlorides.^a,b a^Reaction condition: WA (1 equiv), aromatic hydroximidoyl chloride (1.2 equiv), Triethylamine (0.1 equiv), in DMF at 0 °C for 3 h. ^b^Yields refer to the isolated yields after column chromatography.

The structure of the products **W-1a** and **W-1b** were elucidated by 1D and 2D NMR analysis (Tables 1 and 2). ^1^H and ^13^C NMR spectra of major strereoisomer **W-1b** revealed that the signals relating to B/C/D ring systems remained largely unaltered. But the profound shifting of resonance positions of H2 and H3 from δ 6.0 and δ 7.0 to δ 4.82 and δ 5.10 respectively in the ^1^H NMR combined with the shifting of C2 and C3 signals from δ 132.8 and 146.7 to 59.1 and 84.2 respectively in ^13^C NMR provided the clear indication of formation of 2-isoxazoline ring in **W-1b** (Table 1). The coupling constant of 11.9 Hz between H2 and H3 was consistent with the 2,3-(*cis*)-annulations of the heteroring. Similar types of signal shifts were observed for the other isomer **W-1a** (Table 2). However in the NOESY of **W-1a**, peaks for H2 and H4 showed a strong correlation whereas no such correlation was observed in the spectrum of **W-1b** indicating β,β ring juncture (Fig. 2A). From the mechanistic point of view, depending on the dipole orientation of nitrile oxide relative to the double bond, four diastereomers (two *cis* and two *trans*) are possible from the cycloaddition. Further, the regioisomer in which the oxygen of the nitrile oxide is attached to the β-carbon of the α,β-unsaturated system is preferred due to the favourable Large-Large HOMO-LUMO favourable orbital interaction (Fig. 2B). The attack of the dipole from above the general plane of the sterane framework (the β side) is less feasible in WA because of steric interactions with 4,5,10 substituents, forming the stereoisomer having β,β-ring juncture in major quantity. Finally, we established the structures of both the stereoisomers by HMBC and HSQC and all signals are listed in Tables 1 and 2. The reaction proceeded well with other substituted benzonitrile oxides, most of which formed the fused isoxazoline having the β,β-ring juncture as the major stereoisomer. The para substituted benzonitrile oxides reacted much more effectively than the meta or ortho substitution ones (Fig. 3). Among para substituted aromatic aldehydes, those with a electron withdrawing group (EWG) reacted much faster and with better yield. With aliphatic hydroximidoyl chloride, the reaction yielded a complex mixture of different products which could not be characterized.

**Table 1:** ^1^H NMR and ^13^C NMR assignments of WA and W-1b.

Position	**WA**	^13^C NMR	**W-1b**	^13^C NMR
	^1^H NMR (DMSO, 400 MHz)		^1^H NMR (DMSO, 400 MHz)	
1	—	203.3	—	203.6
2	6.0 (d, *J* = 9.8 Hz)	132.8	4.82 (d, *J* = 12 Hz)	59.1
3	7.0 (dd, *J* = 9.8, 6.3 Hz)	146.7	5.10 (dd, *J* = 12.00, 3.6 Hz)	84.2
4	3.5 (dd, *J* = 6.3, 4.2 Hz)	70.1	3.46 (d, *J* = 3.4 Hz)	72.3
4-OH	5.6 (d, *J* = 4.2 Hz)		6.13 (d, *J* = 3.3 Hz)	
5	—	64.7	—	62.4
6	3.1 (brs)	60.2	3.3 (bs)	57.2
18	0.5 (s)	12.8	0.48 (s)	11.6
19	1.2 (s)	17.7	1.12 (s)	15.2
21	0.7 (d, *J* = 6.3 Hz)	16.4	0.78 (d, *J* = 6.6 Hz)	13.5
22	4.2 (m)	79.0	4.2 (d, *J* = 12.3 Hz)	77.9
23	2.5 (m)	28.8	2.5 (m)	29.7
24	—	156.3	—	155.0
25	—	126.9	—	125.9
26	—	166.9	—	165.7
27a,b	4.02 (dq, *J* = 16.6, 5.3)	56.0	4.10 (dq, *J* = 16.6; 5.3)	56.3
27-OH	4.52 (t, *J* = 5.3)		4.5 (t, *J* = 5.3)	—
28	1.88 (s)	21.4	2.04 (s)	20.3

**Table 2:** ^1^H NMR and ^13^CNMR of WA and W-1a.

position	**WA**	^13^C NMR	**W-1a**	^13^C NMR
	^1^H NMR (DMSO, 400 MHz)		^1^H NMR (DMSO, 400 MHz)	
1	—	203.3	—	204.2
2	6.0 (d, *J* = 9.8 Hz)	132.8	4.5 (m)	55.8
3	7.0 (dd, *J* = 9.8, 6.3 Hz)	146.7	4.9 (d, *J* = 10.9)	85.2
4	3.5 (dd, *J* = 6.3, 4.2 Hz)	70.1	3.3 (s)	71.8
4-OH	5.6 (d, *J* = 4.2 Hz)	—	6.0 (s)	—
5	—	64.7	—	63.1
6	3.1 (brs)	60.2	3.3 (s)	56.6
18	0.5 (s)	12.8	3.3 (s)	11.6
19	1.2 (s)	17.7	1.2 (s)	15.6
21	0.7 (d, *J* = 6.3 Hz)	16.4	0.9 (d, *J* = 6.3 Hz)	13.5
22	4.2 (m)	79.0	4.2 (d, *J* = 12.3 Hz)	78.0
24	—	156.3	—	155.1
25	—	126.9	—	125.9
26	—	166.9	—	165.8
27a,b	4.02 (dq, *J* = 16.6; 5.3)	56.0	4.1 (m)	54.9
27-OH	4.52 (t, *J* = 5.3)	—	4.5 (m)	—
28	1.88 (s)	21.4	2.0 (s)	20.3

### Withaferin A isoxazolines are cytotoxic and the most potent derivative (W-2b) induces premature senescence in proliferating cancer cells

**Figure 4:**
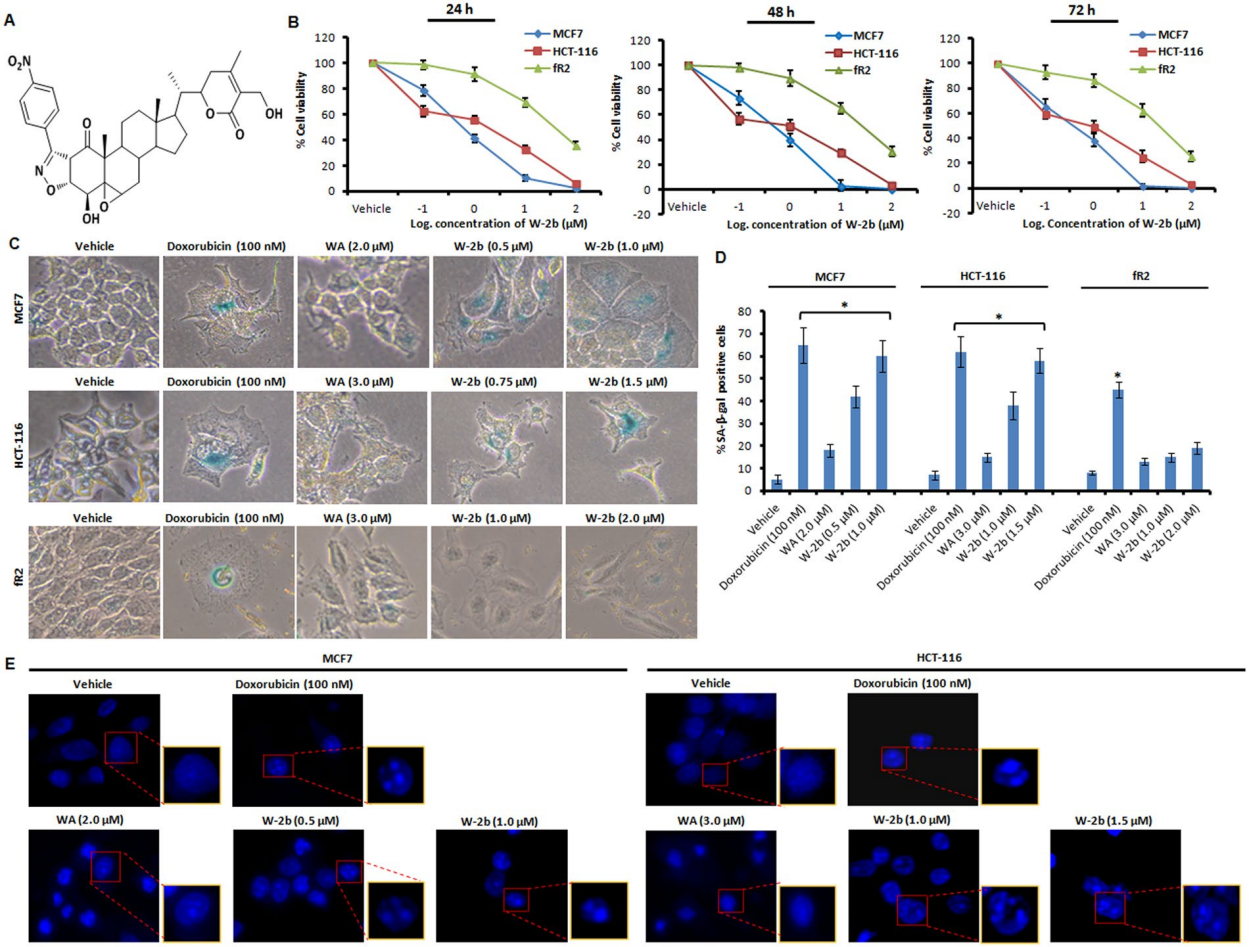
**W-2b** is cytotoxic and induces premature senescence in cancer cells. (**A**) Structure of novel isoxazoline derivative of withaferin A (**W-2b**). (**B**) Graphs showing the percent cell viability of MCF7, HCT-116 and fR2 cells in response to logarithmic concentrations of **W-2b** for 24 h, 48 h, and 72 h. (**C**) Effect of vehicle, doxorubicin (100 nM), WA, and various concentrations of **W-2b** on SA-β-gal activity in MCF7, HCT-116, and fR2 cells. Original magnification 20x. (**D**) Bar graph showing quantification of SA-β-gal positive cells. Error bars: mean ± s.d. *P < 0.05. (**E**) After treatment, MCF7 and HCT-116 cells were stained with DAPI containing mounting media for 15–20 min and observed for the formation of SAHF under Floid Cell Imaging Station. Insets (red boxes) showing magnified images for proper visualization of SAHF. Data are representative of three independent experiments. Original magnification 20x.

Recent reports reveal that WA possesses anti-tumor activities against breast and colorectal cancer models^23–26^. In order to check the cytotoxic activities of WA isoxazoline derivatives against human breast cancer MCF7 and colorectal cancer HCT-116 cells, we performed cell viability assay through MTT dye reduction method and the cytotoxicity results are presented in terms of IC_50_ values in Table 3. The parent WA and isoxazoline derivatives of WA (W-1a to W-12b) displayed significant cytotoxic activities against both the MCF7 and HCT-116 cells. In general, the fused isoxazolines having β,β-ring juncture were found to be more potent than the other corresponding stereoisomer having α,α-ring juncture. The nitro derivative **W-2b** showed the most promising cytotoxic effects among the synthesized molecules in both MCF7 and HCT-116 cell lines with comparatively less toxicity towards human normal breast epithelial (fR2) cells (Fig. 4A,B). We examined the cytotoxicity of **W-2b** in both dose-dependent as well as time-dependent manner in these three cell lines (Table 4). The 24 h IC_50_ values of **W-2b** were 0.881 ± 0.052 µM and 1.48 ± 0.129 µM, the 48 h IC_50_ values: 0.705 ± 0.059 µM and 1.25 ± 0.156 µM, and the 72 h IC_50_ values: 0.682 ± 0.075 µM and 1.03 ± 0.33 µM in MCF7 and HCT-116 cells respectively (Table 4). However, the IC_50_ values of **W-2b** in fR2 cells were 39.66 ± 7.3 µM, 35.5 ± 6.12 µM, and 33.9 ± 8.23 µM in 24 h, 48 h, and 72 h respectively (Table 4). Senescence is an important tumor suppressive mechanism that works as a barrier to uncontrolled cell proliferation^11^. SA-β-gal activity is regarded as a specific marker for cells undergoing senescence^12^. In order to check the ability of our test compound to induce premature senescence, we carried out SA-β-gal activity assays in MCF7 and HCT-116 cells following treatment with indicated concentrations of **W-2b** along with positive control doxorubicin for five days. Indeed, we noticed a remarkable increase in SA-β-gal positive cells (58% in MCF7 and 53% in HCT-116) following treatment with the sub-toxic concentrations of **W-2b** and characteristic senescent features - flattened cellular morphology and bluish nuclear stains were distinctly visible in both the cell lines compared to the vehicle treated cells (Fig. 4C,D). Albeit, the results of SA-β-gal staining demonstrated a substantial increase in the number of cells with distinct senescence phenotype in doxorubicin (100 nM) treated cells whereas very less number of senescent cells were observed in presence of the parent molecule WA even at the higher concentration (2–3 µM). However, some early apoptotic population of cells were visible in WA treatment conditions (Fig. 4C,D). We also confirmed the effect of **W-2b** on fR2 cells through SA-β-gal staining and the results indicated significantly lower number of SA-β-gal positive cells (15–19%) at the sub-toxic doses of **W-2b** (1–2 µM) unveiling the selectivity of the molecule towards proliferating cancer cells (Fig. 4C,D). For further confirmation, we performed senescence-associated heterochromatin foci (SAHF) formation assay through nuclear staining of the cells by DAPI containing mounting media^12,27^. We found a notable increase in SAHF formation with appearance of typical beaded nucleus in cells treated with **W-2b**/doxorubicin, whereas the nucleus of the cells treated with vehicle (DMSO) were totally devoid of such beaded appearances (Fig. 4E). Although there observed some SAHF formation in WA treated cells but the features of early apoptosis were predominating in these cells (Fig. 4E). The unlimited proliferative capability of cancer cells supports the growth of primary tumor that eventually leads to cancer progression and morbidity of the patient^28^. However, the onset of senescence further hinders the division and colony forming ability of proliferating cancer cells^29^. To check the effect of **W-2b** on proliferation of MCF7 and HCT-116 cells, we then employed colony formation assay. Our results obtained a significant number of colonies in vehicle treated wells, whereas, the colony formation was inhibited gradually at the lower doses (0.5 µM for MCF7 and 1.0 µM for HCT-116) and significantly (>70%) at the higher doses (1.0 µM for MCF7 and 1.5 µM for HCT-116) similar to the positive control doxorubicin (100 nM) (Supplementary Fig. S1). As in most cases, the generation of reactive oxygen species (ROS) is linked with loss of cell proliferation and senescence^30^, we sought to determine the effect of **W-2b** on ROS generation in MCF7 and HCT-116 cells. Our results revealed that **W-2b** triggered sufficient quantity of mitochondrial ROS at sub-toxic doses of the molecule within 48 h of treatment similar to the positive control H_2_O_2_ (10 µM), which further supports its anti-proliferative effects in these cells (Supplementary Fig. S2). Together, these findings demonstrate that **W-2b** is potentially cytotoxic and induces premature senescence in proliferating cancer cells.

**Table 3:** Cytotoxicity of WA and derivatives (48 h) in MCF7, HCT-116, and fR2 cells; IC_50_: µM.

Compounds	MCF7	HCT-116	fR2
W-1a	3.8 ± 0.378	5.62 ± 0.412	45.8 ± 5.19
W-1b	1.64 ± 0.267	16.2 ± 0.676	39.2 ± 3.81
W-2a	5.82 ± 0.418	8.2 ± 0.211	52.1 ± 6.6
**W-2b**	**0.705 ± 0.059**	**1.25 ± 0.156**	**35.5 ± 6.12**
W-3a	95.5 ± 5.412	45.8 ± 2.343	>100
W-3b	1.82 ± 0.33	15.9 ± 1.67	85.3 ± 9.5
W-4a	>100	19.6 ± 1.81	>100
W-4b	>100	>100	>100
W-5a	3.52 ± 0.185	63.8 ± 5.433	>100
W-5b	9.7 ± 0.55	42.4 ± 3.189	91.25 ± 7.33
W-6a	2.28 ± 0.265	>100	>100
W-6b	>100	11.51 ± 2.21	63.3 ± 2.5
W-7a	70.64 ± 5.18	8.1 ± 1.655	>100
W-8a	17.6 ± 1.676	7.5 ± 1.129	28.2 ± 4.21
W-9b	>100	15.8 ± 3.311	66.23 ± 7.63
W-10a	58.3 ± 3.24	5.28 ± 0.561	98.1 ± 7.18
W-11a	>100	24.5 ± 2.55	>100
W-11b	83.3 ± 6.512	4.16 ± 0.344	>100
W-12b	26.9 ± 2.224	5.29 ± 0.151	75.6 ± 4.82
WA	1.75 ± 0.376	2.58 ± 0.415	43.32 ± 3.77

IC_50_ values are indicated as mean ± standard deviation of three independent experiments performed.

**Table 4:** Time-dependent cytotoxicity of WA and W-2b in MCF7, HCT-116, and fR2 cells.

Compounds	MCF7	HCT-116	fR2
***24 h, IC***_***50***_ ***: µM***			
WA	9.18 ± 1.358	4.38 ± 1.673	51.12 ± 5.18
W-2b	0.881 ± 0.052	1.48 ± 0.129	39.66 ± 7.3
***48 h, IC***_***50***_ ***: µM***			
WA	1.75 ± 0.376	2.58 ± 0.415	43.32 ± 3.77
W-2b	0.705 ± 0.059	1.25 ± 0.156	35.5 ± 6.12
***72 h, IC***_***50***_ ***: µM***			
WA	1.46 ± 0.291	2.13 ± 0.55	38.34 ± 5.22
W-2b	0.682 ± 0.075	1.03 ± 0.33	33.9 ± 8.23

IC_50_ values are indicated as mean ± standard deviation of three independent experiments performed.

### W-2b triggers cell-cycle arrest and p21^Waf1/Cip1^ upregulation

**Figure 5:**
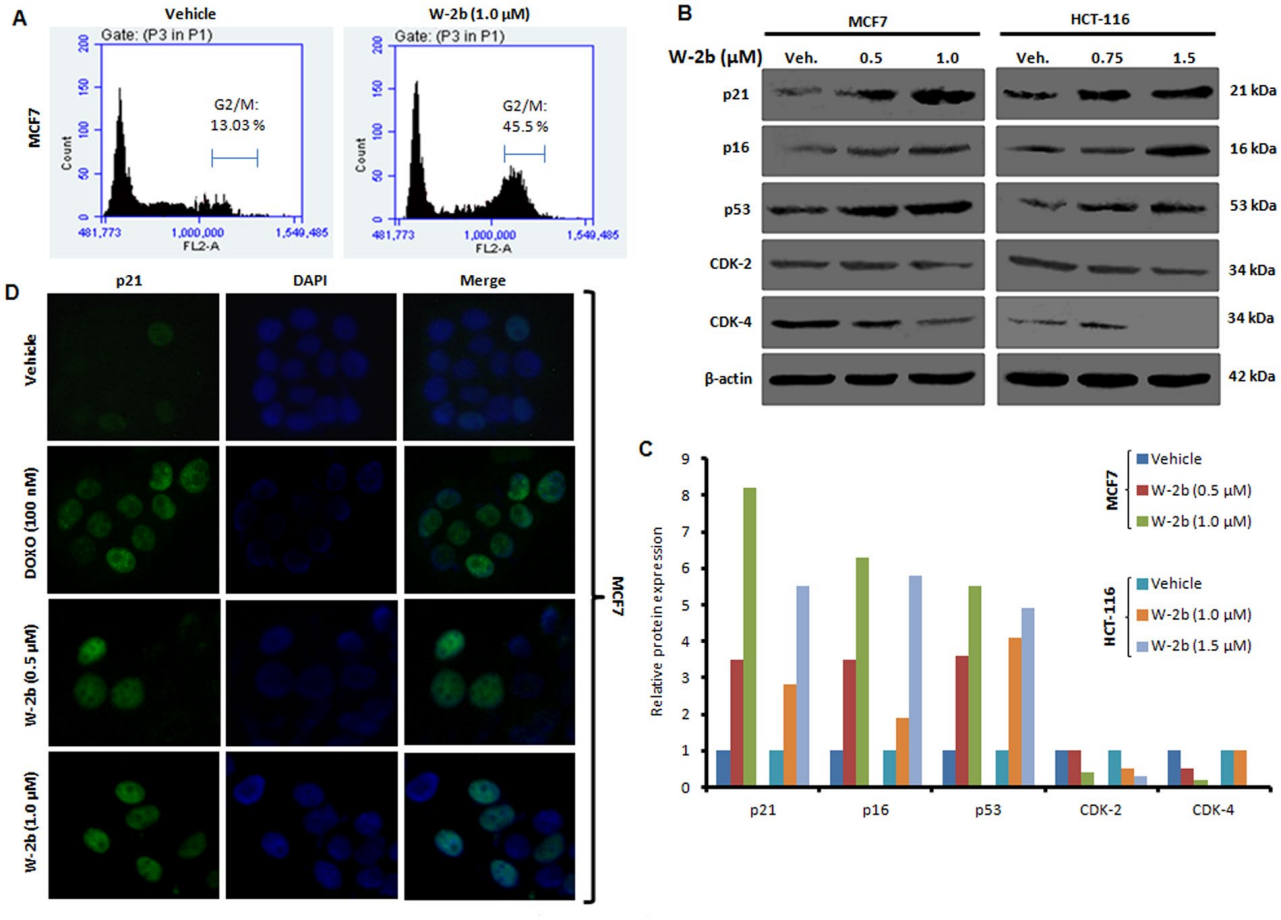
**W-2b** confers cell-cycle arrest and p21 induction in proliferating cancer cells. (**A**) Flow cytometric cell cycle analysis in MCF7 cells treated with vehicle and **W-2b** (1.0 µM) for 48 h. (**B**) MCF7 and HCT-116 cells were treated with vehicle and increasing concentrations of **W-2b** for 48 h; whole cell lysates were prepared and subjected to western blot analysis for the expression of p21, p16, p53, CDK-2, CDK-4 and β-actin. (**C**) Densitometry analysis showing relative protein expression for the above western blots. (**D**) Immunofluorescence analysis results depicting the effect of vehicle, doxorubicin and **W-2b** on induction of p21 in MCF7 cells. Original magnification 20x. Data are representatives of two independent experiments.

Though, therapeutics induced premature senescence is directly correlated with cell-cycle arrest (preferably in the G0/G1 phase), studies also uncovered that senescence can be induced through G2/M arrest^31^. Our cell-cycle analysis experiments through propidium iodide staining demonstrated that **W-2b** (1.0 µM) arrested the MCF7 cells in G2/M phase (45.5%) compared to 13.03% in the vehicle treated cells (Fig. 5A). Alterations in the expression of vital genes occur in the cells undergoing senescence. Cyclin-dependent kinase inhibitor p21 is regarded as a senescent-specific marker, as its upregulation has been documented in almost all cells undergoing senescence^12,18,30^. The expression of p16^INK4a^ (another cyclin-dependent kinase inhibitor) is also an indicative marker of senescence^12^. The p16-mediaed senescence takes place through the retinoblastoma (Rb) pathway suppressing the cyclin-dependent kinases leading to cell-cycle arrest^32,33^. The tumor suppressor p53 (also called as the guardian of the genome) is too considered as a molecular marker of cellular senescence. In response to DNA-damage, the p53 gets activated in cells transmitting directly signals to p21 for the execution of cell-cycle arrest, apoptosis, and/or senescence^33,34^. Induction of p21 suppresses the cyclin-dependent kinases (CDKs), thereby leading to cell cycle arrest and loss of cell proliferation^12^. Correspondingly, our western blotting experiments showed a robust increase in expression of p21 in both the MCF7 and HCT-116 cells along with upregulation of p16, p53, and concomitant downregulation of CDK-2 and CDK-4 expression with increasing concentrations of **W-2b** (Fig. 5B,C). The immunocytochemistry results further confirmed that **W-2b** could induce the expression and nuclear localization of p21 in these cells (Fig. 5D). These collective results strongly support that **W-2b** is a potential inducer of premature senescence through cell-cycle arrest and activation of p21 in proliferating cancer cells.

### W-2b confers induction of Chk2 in proliferating cancer cells

**Figure 6:**
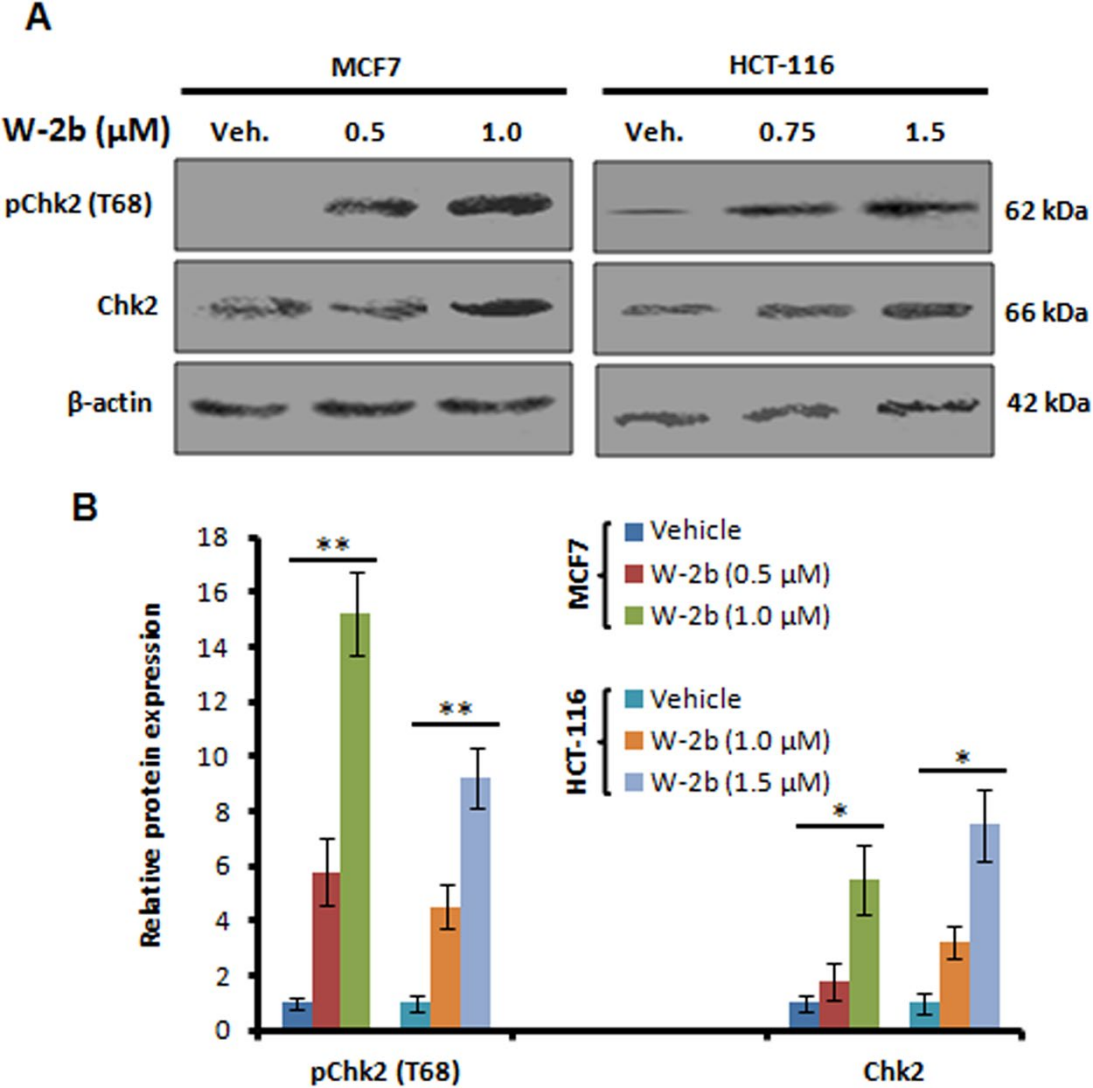
**W-2b** triggers Chk2 activation in cancer cells. (**A**) MCF7 and HCT-116 cells were treated with vehicle and increasing concentrations of **W-2b** for 48 h; whole cell lysates were prepared and subjected to western blot analysis for the expression of pChk2 (T68), Chk2 and β-actin. (**B**) Densitometry analysis of the bands obtained from above experiments. Data are representatives of three independent experiments. Error bars: mean ± s.d. *P < 0.05, **P < 0.01.

Chk2 kinase is an important tumor suppressor protein that preserves genomic stability of the organisms during critical situations such as DNA damage response by inducing cell-cycle arrest to facilitate either DNA repair or apoptosis or senescence^15,16,18^. It serves as a key target for small molecules from natural product/synthetic sources that can be modulated to circumvent cancer cell proliferation, invasion, and metastasis^35,36^. Though our results underscored a substantial provocation of premature senescence in two rapidly proliferating epithelial cancer cells (MCF7 and HCT-116), we hypothesized that **W-2b** might be targeting Chk2 at the molecular level. We carried out western blotting experiments with whole cell lysates prepared from MCF7 and HCT-116 cells after treatment with **W-2b** for 48 h. Interestingly, we found a significant increase in phosphorylation of Chk2 (Thr68) with increasing concentrations of **W-2b**, whereas no/negligible expression of pChk2 were observed in vehicle treated conditions (Fig. 6A,B). We also found a proportionate induction in expression of total Chk2 in a dose-dependent manner in both these cell lines (Fig. 6A,B). These results unveiled that **W-2b** causes phosphorylation-mediated induction of Chk2 in proliferating cancer cells.

### SiRNA-mediated knockdown of Chk2 abrogates the induction of senescence by W-2b

**Figure 7:**
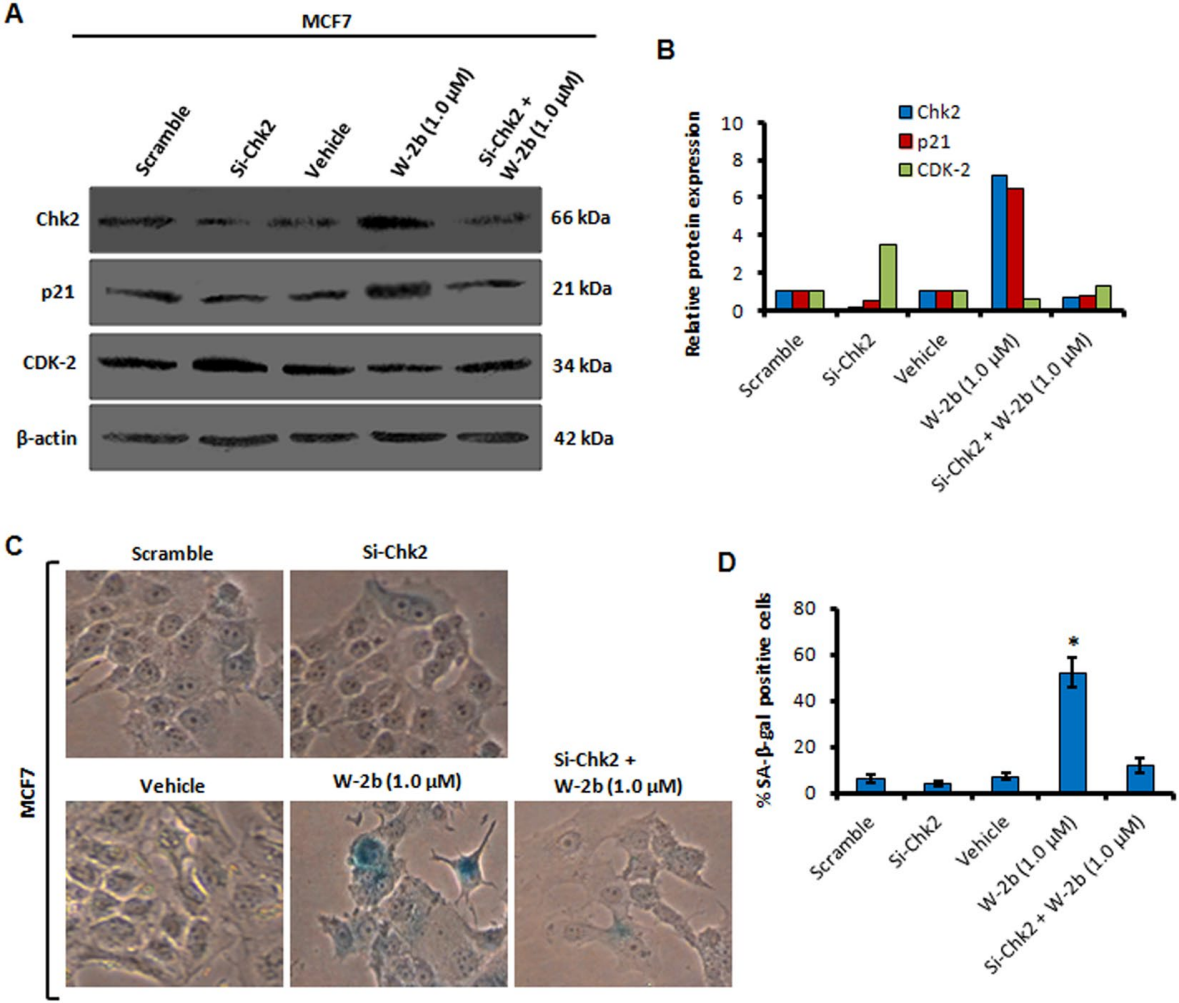
Effect of silencing of Chk2 on induction of senescence by **W-2b**. (**A**) MCF7 cells were either transfected with scramble, Si-Chk2 or treated with vehicle, **W-2b** (1.0 µM) and Si-Chk2 plus **W-2b** (1.0 µM) for 48 h; the whole cell lysates were analysed for the expression of Chk2, p21, and CDK-2 through western blotting. (**B**) Graph shows densitometry analysis of the bands obtained from above experiments. (**C,D**) MCF7 cells were treated under above mentioned conditions and subjected to SA-β-gal staining. Original magnification 20x. Bar graphs: mean ± s.d. of three independent experiments. *P < 0.05.

To confirm whether the molecule (**W-2b**) targets and induces the expression of Chk2 to provoke senescence, we performed SiRNA mediated knockdown of Chk2 in MCF7 cells followed by treatment with **W-2b**. The western blotting results disclosed three fold decrease in expression of Chk2 in Si-Chk2 treated wells with concomitant decrease in p21 expression. However, **W-2b** became unable to induce sufficient Chk2 and, thereby, p21 expression in Si-Chk2 plus **W-2b** (1.0 µM) treated samples (Fig. 7A,B). The CDK-2 expression also remained unaffected in the Si-Chk2 plus **W-2b** treated conditions (Fig. 7A,B). The SA-β-gal assay further verified no such increase in SA-β-gal positive cells in Si-Chk2 plus **W-2b** (1.0 µM) treated cells compared to the **W-2b** treatment alone (Fig. 7C,D). These results strongly demonstrate that **W-2b** promotes premature senescence in a Chk2-dependent manner to limit aberrant cellular proliferation.

### Chk2 activation negatively regulates NM23-H1 signaling axis to control cell proliferation

**Figure 8:**
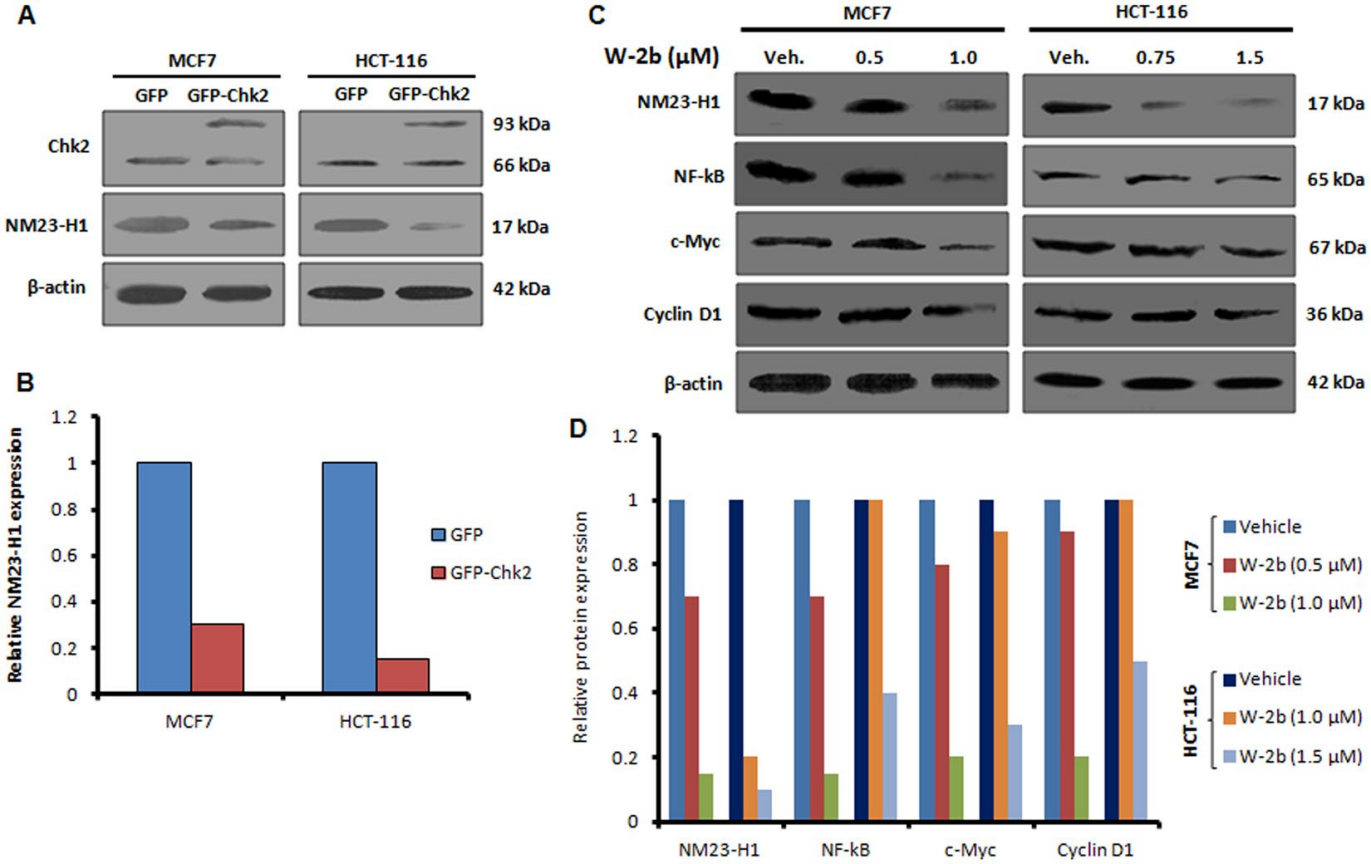
Effect of Chk2 activation on NM23-H1 signaling. (**A**) MCF7 and HCT-116 cells were transfected transiently with GFP and GFP-Chk2 plasmid construct for 48 h. Whole cell lysates were prepared and checked for the expression of Chk2, NM23-H1 and β-actin. (**B**) Bar graph showing relative protein expression as determined by densitometry analysis of the bands. (**C**) Cells were treated with indicated concentrations of **W-2b** for 48 h; whole cell lysates were subjected to immunoblotting experiments for checking the expression of NM23-H1, NF-kB, c-Myc, Cyclin D1 and β-actin. (**D**) Bar graph showing relative protein expression as determined by densitometry analysis of the bands. Blots are representatives of three independent experiments.

To explore the molecular mechanism behind this regulation of cell proliferation and induction of senescence by **W-2b**, and how it induces p21 at the molecular level, we were curious to look at some regulators of cancer cell proliferation and malignancy. Emerging evidences demonstrate that NM23-H1 is an important regulator expressed at the S-phase of the cell cycle leading to cell proliferation in human epithelial breast cancer cell line MCF-10A and human peripheral blood lymphocytes^37^. To assess whether Chk2 activation could affect the intracellular NM23-H1, we transiently overexpressed Chk2 with the help of GFP-Chk2 plasmid construct in MCF7 and HCT-116 cells. Western blotting of the whole cell lysates prepared from the above transfected cells revealed that ectopically overexpressed Chk2 strongly suppressed NM23-H1 expression in both these cell lines compared to the vector/GFP transfected cells (Fig. 8A,B). We also checked the effect of **W-2b** on NM23-H1expression; the results found a steady downregulation in the expression of NM23-H1 in a dose-dependent manner in MCF7 and HCT-116 cells after 48 h of treatment (Fig. 8C,D). We, then, investigated the expression of few downstream target genes of NM23-H1 involved in cell proliferation and tumor growth such as NF-kB, c-Myc and Cyclin D1; the synchronization of these genes regulate CDKs and p21. Our immunoblot results further validated a consistent downregulation in the expression of NF-kB (p65), c-Myc and Cyclin D1 expression in a dose-dependent treatment of **W-2b** in both these cell lines (Fig. 8C,D). Collectively, these data envisaged that Chk2 activation (either by ectopic overexpression or through treatment with **W-2b**) hinders NM23-H1 function and its target genes to regulate CDKs and p21 expression.

### W-2b is an effective inhibitor of tumor growth *in vivo*

**Figure 9:**
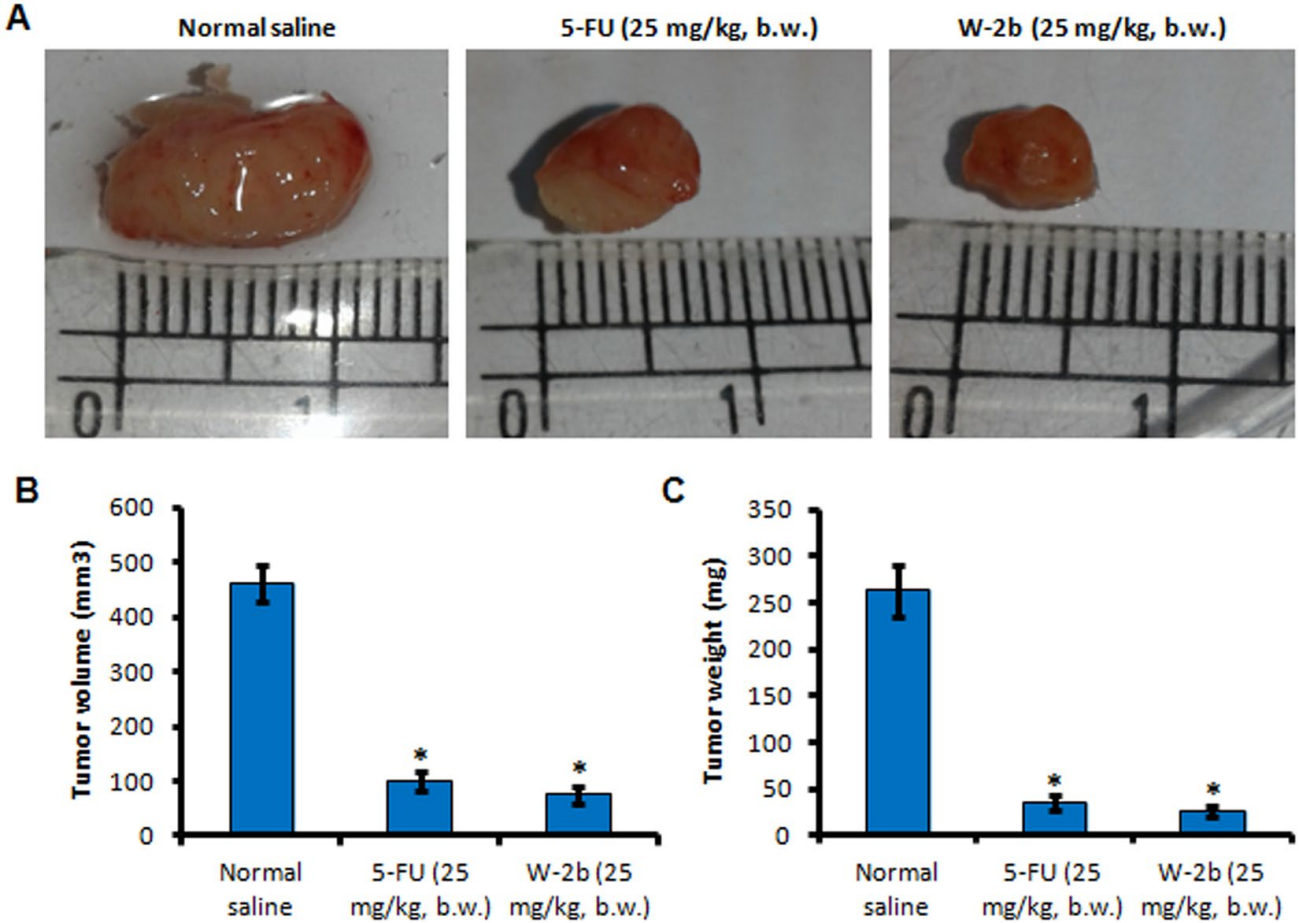
*In vivo* anti-tumor activity of **W-2b** in 4T1 mouse mammary carcinoma model. (**A**) After tumors were grown over the mammary fat pad, animals were injected with either vehicle (normal saline) or 5-FU (25 mg/kg, b.w.) or **W-2b** (25 mg/kg, b.w.) in each alternate days for two weeks. The tumors were dissected out carefully and photographed. (**B**) Bar graph showing tumor volume recorded at the end of the experiment. (**C**) Tumor weight was measured after sacrificing the animals at the end of the experiment. Error bars: mean ± s.d. *P < 0.05.

Though onset of premature senescence impedes the growth of primary tumor and further cancer progression, we were interested to evaluate the *in vivo* efficacy of **W-2b** on tumor growth in 4T1 mouse mammary carcinoma model. Upon intraperitoneal administration of 25 mg/kg, b.w. of **W-2b** in each alternate day for two weeks, we found 83.8% inhibition in tumor volume compared to the 78.4% inhibition in 5-FU (25 mg/kg, b.w.) treated group (Fig. 9A,B). The tumor weight also reduced significantly and the results showed 91.2% inhibition in tumor weight in **W-2b** treated group whereas 86.7% in 5-FU treated group of animals compared to the normal saline treated group (Fig. 9C). Moreover, the animals remained healthy without any serious side effects or mortality throughout the experimental period. These data strongly imply that **W-2b** is a potential and tolerable inhibitor of tumor growth similar to or more efficacious than the standard anticancer drug 5-fluorouracil.

## Discussion

We earlier demonstrated that blocking the irreversible covalent binding in active sites of WA by biological nucleophiles via Michael addition at the β-position retains or even enhance its anticancer potential with minimal side effect making it more target specific^38^. Our recent approach towards the development of ring A modified derivatives of withaferin A successfully generated a 3-azido analogue with strong anticancer activities. Studies from our laboratory demonstrate that 3-azido withaferin A (3-AWA**)** inhibits invasion of cervical and prostate cancer cells and angiogenesis by modulating extracellular prostate-apoptosis response-4 (Par-4)^39^. 3-AWA mediated induction of Par-4 regulates cellular β-catenin to control EMT and invasion in prostate cancer PC-3 and breast cancer MCF7 cells^40^. Another study by Rah *et al*. demonstrated that Par-4 mediated suppression of Bcl-2 by 3-AWA promotes switching of autophagy to apoptosis in prostate cancer cells^41^. Rasool *et al*. described that 3-AWA confers translational attenuation through dephosphorylation of eukaryotic translation initiation factor 4E (eIF4E), that results in inhibition of tumor growth and metastasis^42^. Keeping in mind the potential anticancer activities of 3-AWA, we contemplated that addition at both the α and β-position of the α,β-unsaturated carbonyl system may modify the biological activity further. Considering the promising biological importance of isoxazoline ring system, we intriguingly explored the synthesis of a combined motif involving ring A of WA using 1,3-dipolar cycloaddition reaction on arylnitrile oxides. Another goal was to investigate the regio- and stereoselectivity of the processes vis-à-vis the influence of steric and electronic factors on the ring closures and compare the reactivity of WA ring A against various nitrile oxides. We envisioned that the addition of steric bulk adjacent to the extant functional groups on C-3, essential for hormone-receptor binding, may contribute to a change in biological activity and these derivatives may therefore deserve attention from a pharmacological aspect. Moreover, the vicinity of the angular methyl groups (C-19) to the reaction centre and also the rigidity of the sterane skeleton overall was thought to have a significant influence on the stereo and regiocontrol of the process. In this regard, our medicinal chemistry approach with the ring A modified WA isoxazolines found out a potential lead molecule (**W-2b**) with strong antiproliferative and antitumor activities.

**Figure 10:**
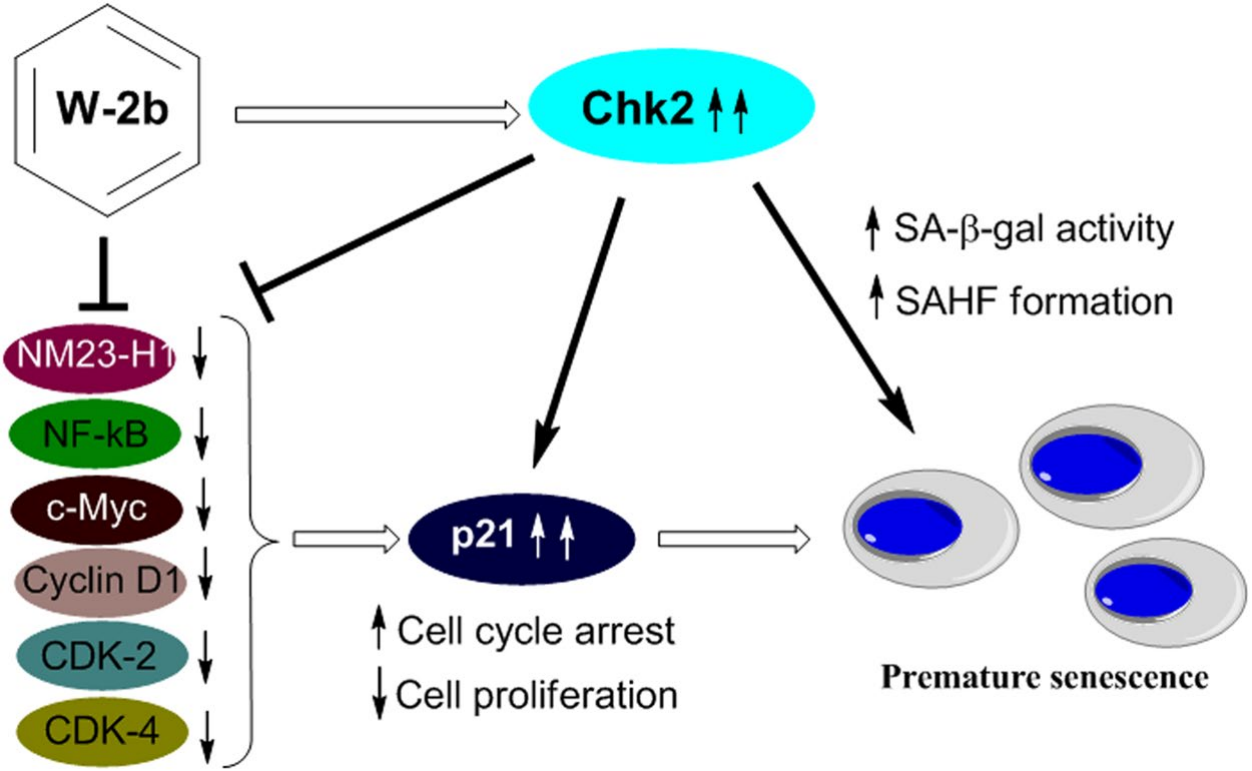
Schematic diagram represents the proposed mechanism of action of **W-2b**. **W-2b** triggers Chk2 activation in cancer cells, thereby, inhibiting cell proliferation by NM23-H1/NF-kB/c-Myc/Cyclin D1/CDK-2/CDK-4 signaling axis. These effects of **W-2b** further induce senescence-specific marker p21 expression and its nuclear localization to promote premature senescence in proliferating cancer cells.

Senescence is an important biological phenomenon in normal as well as cancer cells that facilitates as a barrier to control aberrant cell proliferation^11^. In response to various cellular stresses, including genotoxic stress by DNA damaging agents, proliferating cells cease to divide permanently and attain an enlarged morphology. Growth arrest occurs usually in the G1 or G2/M phases of the cell cycle. Senescence prevents the growth of damaged or stressed cells that are harmful for the organism^12^. Chk2 kinase is an important component of the DNA damage checkpoint signaling pathway, which is activated directly by ATM in response to the ionizing radiation^14^. Chk2, in turn, activates and stabilizes major tumor suppressor proteins *viz*. p53 to carry out antitumor activities by inducing cell cycle arrest and apoptosis^15^. Ample evidences demonstrate that Chk2 is a suitable target that can be modulated to promote senescence in proliferating cancer cells^17,18^. Though many small molecules from natural, semi-synthetic as well as synthetic sources are reported to induce premature senescence^19^, the finding of a potential compound that can activate Chk2 to limit uncontrolled proliferation in cancer cells is extremely limited. Our recent approach in this direction uncovered 4′-Demethyl deoxypodophyllotoxin glucoside (4DPG), a natural podophyllotoxin congener from the medicinal plant *Podophyllum hexandrum* as a strong anticancer candidate that modulates Chk2 activity to suppress proliferation, invasion and metastasis in aggressive cancer cells. The molecule (4DPG) also induces premature senescence in p53-defective invasive cancer cells^35,36^. In this study, our hunch for small molecule inducers of Chk2 found out a potential lead from Withaferin A isoxazoline derivatives (**W-2b**) that phosphorylates Chk2 (T68) and induces its expression in two rapidly proliferating cancer cells from diverse tissue origin (MCF7 and HCT-116). Evidence suggests that sub-lethal level of intracellular ROS generation could initiate premature senescence by inducing p21 expression through G1 arrest^30^. Being a key regulator of the cell cycle machinery, p21 controls cell proliferation and DNA replication through regulation of cyclin-dependent kinases (CDKs)^30^. Although p53 is a major transcription factor that regulates p21, studies also found that Chk2 can induce senescence in cancer cells via p21, irrespective of the p53 status of the cell^18^. Indeed, **W-2b** causes a significant increase in senescence phenotypes with remarkable SA-β-gal activity coupled with G2/M cell cycle arrest and induction of p21 in a dose-dependent manner (Fig. 10).

*NM23* gene belongs to the family of nucleoside diphosphate kinases (NDPKs) that catalyze the phosphorylation of nucleoside diphosphates to their corresponding nucleoside triphosphates through oxidative phosphorylation^43^. There are ten NM23 isoforms characterized so far; NM23-H1, encoding for a 17 kDa protein in human, is the most studied^44^. It was initially identified as a metastasis suppressor gene because of its reduced expression in highly metastatic mouse melanoma cells^45^. However, handful of evidence clearly demonstrates that overexpression of NM23-H1 is associated with disease progression and poor patient survival in thyroid carcinomas, cervical cancer, neuroblastomas and osteosarcoma patients^46^. Though the expression of NM23-H1 in a cell cycle specific manner, its role in regulating metastasis and its loss of control in advance stages of the tumor progression has been understood substantially^47^, how this protein can be modulated to induce premature senescence in proliferating cancer cells has not been explored till date. In this context, we hypothesized that Chk2 activation could suppress the oncogenic signaling of NM23-H1 in proliferating cancer cells. Surprisingly, our study found that ectopically induced Chk2 downregulates NM23-H1 expression substantially in both the MCF7 and HCT-116 cells after 48 h of post transfection. We also found a steady downregulation in the expression of NM23-H1 in MCF7 and HCT-116 cells after 48 h of treatment with increasing concentrations of **W-2b** compared to the vehicle treated cells (Fig. 8C). The nuclear factor kB (NF-kB), one of the major transcription factors, is known to induce inflammatory responses, cancer cell survival, proliferation and tumor progression^48^. Though a splicing variant of NM23-H1 is reported to negatively regulate NF-kB signalling^49^, the effect of NM23-H1 itself on the regulation of NF-kB is poorly understood. The expression of c-Myc oncogene is associated with growth, differentiation and advancement of many tumors^50^. Studies also reported that c-Myc oncogene contains two responsive elements on its promoter for the NF-kB family of transcription factors and classical NF-kB (p65/p50) is a potential activator of the c-Myc promoter^51^. Accumulating evidence revealed that NF-kB activates Cyclin D1 expression at the transcriptional level through direct binding of NF-kB to multiple sites in the Cyclin D1 promoter and promote G1 to S phase transition^52^. Cell-cycle progression through G1 phase of the cell cycle requires the association of specific cyclin: cyclin-dependent kinase (CDK). Cyclin D1, CDK-2 and CDK-4 are the key players in this regard, forming stable complexes leading to G1/S transition^53^. In mammalian cells, p21 binds to and inhibits the kinase activity of several cyclin-dependent kinases including CDK-2 and CDK-4 leading to growth arrest at specific phases of the cell cycle^54^. Rationally, **W-2b** treatment suppressed the expression of NF-KB (p65), c-Myc, Cyclin D1 along with CDK-2 and CDK-4 at its sub-toxic doses in both MCF7 and HCT-116 cells (Fig. 10). These results also support the induction of p21 in these cells by **W-2b** in a dose-dependent manner.

In conclusion, our study reports a potential lead from Withaferin A isoxazoline derivatives (**W-2b**) that induces premature senescence as an antitumor safeguard mechanism against proliferating cancer cells through activation of tumor suppressor Chk2. It’s (**W-2b**) strong *in vivo* efficacy and tolerability claim for its further development as a therapeutically relevant anticancer candidate.

## Materials and Methods

### Biology

#### Cell culture and reagents

The cell lines used in this study were procured from American Type Culture Collection (ATCC), Manassas, USA and European Collection of Authenticated Cell Cultures (ECACC), Porton Down, Salisbury, UK. The MCF7, HCT-116, and fR2 cells were cultured in RPMI-1640 medium supplemented with 10% fetal bovine serum (Gibco) and 1% penicillin/streptomycin (Sigma) in a humidified CO_2_ incubator (New Brunswick Galaxy 170 R) with 5% CO_2_. For the treatment, withaferin A and its derivatives were solubilized in dimethylsulfoxide (DMSO) and delivered to the cells in culture through complete medium. The DMSO was treated as vehicle in each experiments performed. Reagents such as paraformaldehyde, Triton X-100, phenylmethylsulfonyl fluoride (PMSF), dithiothreitol (DTT), DMSO, crystal violet, 2′,7′-dichlorofluorescin diacetate (DCFDA) and Bradford’s reagent were purchased from Sigma-Aldrich. X-gal (5-bromo-4-chloro-3-indolyl-beta-D-galacto-pyranoside) substrate was obtained from Thermo Fisher Scientific. Protease inhibitor cocktail was procured from Roche. The UltraCruz DAPI mounting media and antibodies for human p21^Waf1/Cip1^, NM23-H1, NF-kB, c-Myc, Cyclin D1, CDK-2, CDK-4 and BCL-2 were procured from Santa-Cruz Biotechnology. The anti-β-actin antibody and the secondary antibodies such as anti-rabbit IgG and anti-mouse IgG were procured from Sigma-Aldrich. Fluorescence-conjugated anti-mouse secondary antibody Alexa-Fluor 488 (green) was purchased from Thermo Fisher Scientific.

#### Cell viability assay

The cell viability assay was performed according to the procedure previously described with minor modifications^39^. Briefly, MCF7, HCT-116 and fR2 cells were plated in 96 well plates at a density of 5 × 10^3^ cells per well and incubated overnight. On the next day, varying concentrations (100, 10, 1 and 0.1 µM) of WA and the isoxazoline derivatives were added along with DMSO as vehicle for 48 h. MTT dye solution (2.5 mg/mL) was introduced to the cells in medium 4 h before the completion of the treatment period and the formed formazan crystals were solubilized with DMSO. Optical density was measured with the help of a UV-visible spectrophotometer coupled with microplate reader (TECAN, Infinite M200 Pro), the percent inhibition was calculated and IC_50_ values were determined with the help of GraphPad Prism software (GraphPad software Inc. CA, USA).

#### SA-β-gal assay

The procedure was followed as described previously with some modifications^27^. Briefly, MCF7 and HCT-116 cells (0.4 × 10^6^ per well) were seeded in six-well plates and treated with indicated concentrations of **W-2b** along with WA, vehicle and positive control doxorubicin for five days. Cells were accordingly washed twice with PBS, fixed with 4% paraformaldehyde for 5 min, rinsed twice with PBS and incubated with freshly prepared staining solution (40 mM citric acid/Na phosphate buffer, 5 mM K_4_[Fe(CN)_6_] 3H_2_O, 5 mM K_3_[Fe(CN)_6_], 150 mM sodium chloride, 2 mM magnesium chloride and 1 mg/ml X-gal in distilled water, pH 6.0) for 48 h at 37 °C. Stained cells were thoroughly washed and air dried in dark. Cells were then observed under bright field microscope (NIKON) for the SA-β-gal positive cells and images were captured at 20x magnification.

#### SAHF detection

The senescence-associated heterochromatin foci (SAHF) detection method was carried out as previously described by our group^27^. About 20 × 10^3^ cells/well were seeded in 8 well chamber slides and treated with vehicle, doxorubicin, WA, and **W-2b** for five days. Subsequently, these cells were washed with PBS and fixed with 4% paraformaldehyde (w/v) at room temperature for 10 min. Cells were then washed with PBS, stained and mounted with DAPI containing mounting media (Invitrogen). Fluorescence images were captured with Floid cell imaging station (Thermo Scientific) using 20x objective.

#### Flow cytometric cell cycle analysis

MCF7 cells were seeded at a density of 0.4 × 10^6^ cells/well in 35 mm dishes and incubated overnight at 37 °C and 5% CO_2_. Next day, cells were treated with vehicle and **W-2b** at indicated concentrations for 48 h. They were then trypsinized, centrifuged at 2000 rpm for 3 min, washed twice with ice-cold PBS and fixed with ice-cold 70% ethanol (v/v) for 1 hour at 4 °C. Cells were then pelleted down at 2000 rpm for 3 min, washed with PBS and incubated first with 200 μg/ml of RNase A in PBS at 37 °C water bath for 90 min and then with 50 μg/mL of propidium iodide (PI) at room temperature for 30 min in dark. The samples were then analyzed by BD Accuri C6 flow cytometer (BD Biosciences).

#### Western blotting

Western blot analysis was carried out with MCF7 and HCT-116 cells as per the indicated conditions (figure legends) as previously described^39^. Briefly, cells (0.5 × 10^6^/well in 6 well plates) after treatment were harvested, washed with chilled PBS and lysed with lysis buffer containing; HEPES 1 mM, KCl 60 mM, NP-40 0.3%, EDTA 1 mM, DTT 1 mM, sodium orthovanadate 1 mM, PMSF 0.1 mM, protease inhibitor cocktail. Cell extracts were centrifuged at 12,000 rpm for 10 min at 4 °C, the supernatants were collected and protein estimation was performed with Bradford’s reagent. Equal quantity of protein (20 µg) from each sample was employed for gel electrophoresis, transferred to PVDF membranes, blocked with 5% non-fat milk and incubated with primary antibody (1: 1000 dilution) overnight at 4 °C. Membranes were subsequently washed and probed with species-specific secondary antibodies coupled to horse-radish peroxidase. Immunoreactive proteins were detected with the help of Western Bright ECL chemiluminescent HRP substrate (Advansta Inc. CA, USA) and exposed over the CL-XPosure film (Thermo Scientific).

#### Immunofluorescence staining

MCF7 cells were seeded in 8 well chamber slides at a density of 0.5 × 10^5^ cells/well. Cells were further treated with vehicle, doxorubicin and **W-2b** as per the indicated conditions for 48 h. Subsequently, immunocytochemical analysis was carried out following the published protocol^40^. Images were captured under Floid Cell Imaging Station (Thermo Scientific) at 20x magnification.

#### siRNA knockdown experiments

MISSION^®^ esiRNAs for human *CHEK2* (EHU158481), were procured from Sigma-Aldrich (Sigma-Aldrich, St. Louis, MO, USA) and transfection experiments were performed using oligofectamine transfection reagent (Thermo Fisher Scientific) according to the manufacturer’s instructions.

#### Transient transfection

MCF7 and HCT-116 cells were harvested and transfected with GFP and GFP-Chk2 plasmid construct (generously gifted by Dr. Domenico Delia, Fondazione IRCCS Istituto Nazionale Tumori, Italy) using Neon Transfection System (Invitrogen) according to the manufacturer’s instruction.

#### Clonogenic assay

The experiment was carried out according to the standardized protocol with some modifications^40^. MCF7 and HCT-116 cells were trypsinized properly, seeded in 6 well plates at a density of 1 × 10^3^ cells/well and incubated overnight. Then treatment was given to the cells with vehicle, doxorubicin and various concentrations of **W-2b** for five days. Cells were then washed, fixed with 4% paraformaldehyde for 10 min, rewashed twice and stained with 0.25% crystal violet solution for 1 h. The wells containing the cells were then washed thoroughly with distilled water to remove any extra stains and then air dried overnight. The plate was then observed under an inverted microscope and colonies from three random fields were counted, averaged and photographed with NIKON camera (D3100) at 4x magnification.

#### ROS determination assay

The procedure followed was according to the protocol previously described by our group^41^. Accordingly, cells were plated in 12-well plates at a density of 50 × 10^3^ cells/well, incubated overnight and then treated with vehicle and increasing concentrations of **W-2b** for 48 h. Two hours before the completion, H_2_O_2_ (+ve control) was added to the cells in indicated wells and then ROS dye (DCFDA) was added to the cells and further incubation was done in dark for 30 min. Cells were successively washed with PBS thoroughly and images were captured with Floid Cell Imaging Station (Thermo Scientific) at 20x magnification. Fluorescent intensity was measured with the help of a fluorescence spectrometer coupled with microplate reader (TECAN, Infinite M200 Pro).

#### Experimental animals

All animals used in this study were bred and maintained at the central animal facility of Indian Institute of Integrative Medicine, Jammu, India. Animals were maintained at 20–25 °C in a 12 h light dark cycle, routinely monitored for their diet and water consumption and proper sanitations were maintained to avoid any risk of possible pathogenic contamination. Animal studies were performed in accordance with the experimental guidelines that were approved by the Animal Ethics Committee of the institute “CPCSEA” (IAEC No. 51/02/15). During the animal experiments, special handling and care were taken in a humane way, so that no extra pains/injuries were imparted to the animals. To minimize the mortality of animals during experimentation, only a limited number of animals were employed to yield the statistically significant results.

#### *In vivo* studies for tumor growth

The experiment was performed according to the pre-standardized protocol with minor modifications^42^. To evaluate the *in vivo* anti-tumor efficacy of **W-2b**, healthy female Balb/c mice (25–30 g) were taken. Animals were randomized into three groups, and six animals were taken per group. For the tumor cells implantation, mouse mammary carcinoma 4T1 cells (1 × 10^6^ per 200 μL) diluted in serum-free RPMI medium were injected subcutaneously into the mammary pad of each mouse around the second right mammary gland. A week after tumor cell implantation, when the palpable mammary tumors develop, mice were injected intraperitoneally with either vehicle (normal saline) or 5-FU (25 mg/kg/b.w.) or **W-2b** (25 mg/kg/b.w.) in each alternative day for two weeks. Tumor sizes were measured in each alternate day after tumor cell injection, and the body weight was recorded once in a week. Mice were sacrificed on the 15^th^ day after treatment initiation, and tumors were dissected out carefully from the mammary pad area.

#### Statistical analysis

Data were expressed as the mean ± standard deviation of three independent experiments performed and analyzed by Student’s *t*-test. IC_50_ values were determined with the help of GraphPad Prism software Version 5.0 (GraphPad Software, Inc., USA) by taking the log of inhibitor vs. response. A 2-sided value of *P < 0.05 was considered significant in all cases.

### Chemistry

#### General information

^1^H and ^13^C NMR spectra were recorded on 400 and 500 MHz spectrometers with TMS as internal standard. Chemical shifts are expressed in parts per million (*δ* ppm). *J* values are given in Hz and s, d, dd, t, q, m abbreviations correspond to singlet, doublet, doublet of doublet, triplet, quartet, multiplet respectively. Silica gel coated aluminium plates were used for TLC. The products were purified by column chromatography on silica gel (100–200 mesh) using petroleum ether–ethyl acetate as the eluent to obtain the pure products. Exact mass of all products were analysed by using HRMS having QTOF analyser. Reagents used were mostly purchased from Sigma Aldrich.

#### General Procedure for the synthesis of the cis-fused isoxazoline derivatives of Withaferin A

To a solution of aromatic hydroximidoyl chloride (1.2 equiv) in DMF at 0 °C was added Et_3_N (0.1 equiv) first and then WA (1 equiv) after ten minutes. The reaction was allowed to stir for 3 hours at 0 °C and after completion of the reaction; the reaction mixture was diluted with ethyl acetate and extracted with water (5 mL) and brine (5 mL). The organic layer was evaporated and the residue was purified by flash column chromatography (petroleum ether/EtOAc) (7:3) to afford the product as white solid powder.

***4-chlorophenyl-2-isoxazoline withaferin A (W-1a)***. White solid powder; ^1^H NMR (500 MHz, DMSO) *δ* 7.73 (d, *J* = 7.7 Hz, 2H), 7.60 (d, *J* = 7.5 Hz, 2H), 6.06 (s, 1H), 4.93 (d, *J* = 10.9 Hz, 1H), 4.63–4.54 (m, 2H), 4.29 (d, *J* = 12.4 Hz, 1H), 4.16–4.12 (m, 2H), 3.34 (bs, 2H), 2.42 (d, *J* = 16.9 Hz, 2H), 2.11 (d, *J* = 17.5 Hz, 1H), 2.02 (s, 3H), 1.89 (d, *J* = 11.0 Hz, 1H), 1.76 (d, *J* = 31.6 Hz, 2H), 1.55 (d, *J* = 9.8 Hz, 2H), 1.31–1.29 (m, 3H), 1.23 (s, 3H), 1.20 (s, 3H), 1.15 (d, *J* = 13.1 Hz, 4H), 1.04 (d, *J* = 9.4 Hz, 3H), 0.91 (d, *J* = 5.3 Hz, 3H), 0.62 (s, 3H). ^13^C NMR (126 MHz, DMSO) *δ* 204.29 (s), 165.80 (s), 156.58 (s), 155.13 (s), 135.88 (s), 129.98 (s), 129.07 (s), 126.90 (s), 125.93 (s), 85.21 (s), 78.01 (s), 71.89 (s), 63.16 (s), 56.69 (s), 56.18 (s), 55.86 (s), 54.99 (s), 51.46 (s), 49.06 (s), 43.22 (s), 42.52 (s), 38.90 (s), 38.87 (s), 30.95 (s), 29.74 (s), 29.52 (s), 27.00 (s), 24.36 (s), 20.47 (s), 20.39 (s), 15.62 (s), 13.53 (s), 11.69 (s). HRMS ESI: m/z calcd. for C35H43ClNO7 (M + H)^+^ 624.2728, found 624.2708.

***4-chlorophenyl-2-isoxazoline withaferin A (W-1b)***. White solid powder; ^1^H NMR (400 MHz, CDCl_3_) *δ* 7.74 (d, *J* = 8.5 Hz, 2H), 7.39 (d, *J* = 8.5 Hz, 2H), 5.17 (dd, *J* = 11.9, 3.4 Hz, 1H), 4.60 (d, *J* = 11.9 Hz, 1H), 4.4–4.3 (m, 3H), 3.70 (d, *J* = 3.4 Hz, 1H), 3.36 (s, 1H), 2.55–2.39 (m, 1H), 2.18 (d, *J* = 11.9 Hz, 1H), 2.06 (s, 3H), 1.97 (d, *J* = 2.8 Hz, 1H), 1.91 (d, *J* = 9.2 Hz, 2H), 1.62–1.54 (m, 3H), 1.33 (s, 3H), 1.26 (s, 3H), 1.17–1.03 (m, 3H), 0.88 (d, *J* = 6.5 Hz, 3H), 0.85–0.78 (m, 1H), 0.62 (d, *J* = 8.2 Hz, 2H), 0.53 (s, 3H). ^13^C NMR (101 MHz, CDCl_3_) *δ* 203.04 (s), 166.98 (s), 153.59 (s), 152.82 (s), 136.71 (s), 129.21 (s), 128.32 (s), 127.12 (s), 125.71 (s), 83.17 (s), 78.66 (s), 73.69 (s), 62.74 (s), 59.27 (s), 58.34 (s), 57.47 (s), 56.39 (s), 51.85 (s), 51.00 (s), 42.34 (s), 42.21 (s), 38.60 (s), 38.24 (s), 31.15 (s), 29.88 (s), 29.77 (s), 29.71 (s), 27.21 (s), 24.13 (s), 20.09 (s), 20.04 (s), 15.29 (s), 13.31 (s), 11.37 (s). HRMS-ESI: m/z calcd. for C35H43ClNO7 (M + H)^+^ 624.2728, found 624.2728.

***4-nitrophenyl-2-isoxazoline withaferin A (W-2a)*** *.* White solid powder; ^1^H NMR (400 MHz, Pyr) *δ* 8.33 (d, *J* = 8.6 Hz, 2H), 8.18 (d, *J* = 8.7 Hz, 2H), 5.56 (d, *J* = 11.2 Hz, 1H), 5.04 (dd, *J* = 11.3, 2.1 Hz, 2H), 4.91 (d, *J* = 11.7 Hz, 1H), 4.80 (d, *J* = 11.7 Hz, 1H), 4.42 (d, *J* = 13.1 Hz, 1H), 4.12 (s, 1H), 2.88 (s, 1H), 2.51–2.38 (m, 1H), 2.21 (s, 3H), 2.14 (dd, *J* = 18.2, 3.3 Hz, 2H), 1.99–1.86 (m, 2H), 1.80 (s, 3H), 1.56 (s, 1H), 1.52 (s, 2H), 1.44 (dd, *J* = 17.6, 8.6 Hz, 3H), 1.33 (dd, *J* = 16.2, 9.1 Hz, 2H), 1.27 (s, 3H), 1.12 (dd, *J* = 12.9, 5.7 Hz, 1H), 0.97 (d, *J* = 6.6 Hz, 3H), 0.94–0.86 (m, 3H), 0.53 (s, 3H). ^13^C NMR (126 MHz, Pyr) *δ* 205.68 (s), 168.02 (s), 158.34 (s), 155.67 (s), 136.20 (s), 129.99 (s), 128.93 (s), 126.45 (s), 124.74 (s), 88.37 (s), 79.94 (s), 74.03 (s), 65.86 (s), 58.99 (s), 57.97 (s), 57.73 (s), 57.23 (s), 53.28 (s), 51.54 (s), 45.37 (s), 44.17 (s), 40.61 (s), 32.98 (s), 32.80 (s), 31.74 (s), 31.57 (s), 28.76 (s), 25.88 (s), 22.48 (s), 21.79 (s), 17.80 (s), 15.04 (s), 12.91 (s). HRMS ESI**:** m/z calcd. for C35H43N2O9 (M + H)^+^ 635.2969, found 635.2959.

***4-nitrophenyl-2-isoxazoline withaferin A (W-2b)*** *.* White solid powder; ^1^H NMR (400 MHz, Pyr) *δ* 8.37 (d, *J* = 8.7 Hz, 2H), 8.28 (d, *J* = 8.7 Hz, 2H), 5.66 (dd, *J* = 11.9, 3.6 Hz, 1H), 5.16 (d, *J* = 11.9 Hz, 1H), 4.86 (d, *J* = 11.7 Hz, 1H), 4.75 (d, *J* = 11.7 Hz, 1H), 4.29 (d, *J* = 12.9 Hz, 1H), 4.14 (d, *J* = 3.5 Hz, 1H), 3.62 (s, 1H), 3.58 (s, 1H), 2.18 (d, *J* = 13.1 Hz, 2H), 2.07 (s, 3H), 1.87 (s, 2H), 1.71 (s, 3H), 1.46–1.43(m, 4H), 1.40–1.19 (m, 4H), 1.07 (d, *J* = 9.5 Hz, 1H), 0.91 (d, *J* = 11.1 Hz, 3H), 0.79 (d, *J* = 8.9 Hz, 1H), 0.73 (d, *J* = 6.6 Hz, 3H), 0.60 (bs, 1H), 0.40 (s, 3H). ^13^C NMR (101 MHz, Pyr) *δ* 204.28 (s), 166.76 (s), 154.54 (s), 154.31 (s), 149.45 (s), 128.90 (s), 127.76 (s), 125.01 (s), 124.49 (s), 86.35 (s), 78.59 (s), 73.53 (s), 63.82 (s), 59.96 (s), 58.39 (s), 56.98 (s), 56.61 (s), 52.25 (s), 52.01 (s), 43.35 (s), 42.87 (s), 39.38 (s), 39.18 (s), 32.19 (s), 30.93 (s), 30.15 (s), 27.60 (s), 24.77 (s), 21.11 (s), 20.54 (s), 16.33 (s), 13.72 (s), 11.79 (s). HRMS ESI: m/z calcd. for C35H43N2O9 (M + H)^+^ 635.2969, found 635.2961.

***4-bromophenyl-2-isoxazoline withaferin A (W-3a)*** *.* White solid powder; ^1^H NMR (400 MHz, Pyr) *δ* 7.89 (d, *J* = 8.4 Hz, 2H), 7.68 (d, *J* = 8.4 Hz, 2H), 5.47 (d, *J* = 11.2 Hz, 1H), 4.92 (dd, *J* = 16.1, 7.0 Hz, 2H), 4.79 (d, *J* = 11.7 Hz, 1H), 4.41 (d, *J* = 13.2 Hz, 1H), 4.07 (d, *J* = 2.0 Hz, 1H), 2.86 (s, 1H), 2.42–2.40 (m, 1H), 2.21 (s, 3H), 2.18–2.03 (m, 2H), 1.92 (bd, *J* = 36.4 Hz, 2H), 1.78 (s, 3H), 1.53 (d, *J* = 9.8 Hz, 2H), 1.50–1.36 (m, 4H), 1.27 (bs, 1H), 1.12 (d, *J* = 11.1 Hz, 1H), 0.96 (d, *J* = 6.6 Hz, 3H), 0.89–0.87 (m, 3H), 0.77–0.65 (m, 1H), 0.52 (s, 3H). ^13^C NMR (126 MHz, Pyr) *δ* 206.08 (s), 168.09 (s), 158.50 (s), 155.82 (s), 134.53 (s), 130.73 (s), 129.38 (s), 128.93 (s), 126.75 (s), 87.83 (s), 79.92 (s), 74.37 (s), 65.92 (s), 58.97 (s), 58.27 (s), 57.73 (s), 57.22 (s), 53.23 (s), 51.46 (s), 45.32 (s), 44.15 (s), 40.66 (s), 40.60 (s), 32.80 (s), 31.70 (s), 31.50 (s), 28.75 (s), 25.87 (s), 22.48 (s), 21.82 (s), 17.85 (s), 15.03 (s), 12.99 (s). HRMS ESI: m/z calcd. for C35H43BrNO7 (M + H)^+^ 668.2223, found 668.2270.

***4-bromophenyl-2-isoxazoline withaferin A (W-3b)*** *.* White solid powder; ^1^H NMR (400 MHz, CDCl_3_) *δ* 7.66 (d, *J* = 8.6 Hz, 2H), 7.55 (d, *J* = 8.6 Hz, 2H), 5.17 (dd, *J* = 11.9, 3.4 Hz, 1H), 4.60 (d, *J* = 11.9 Hz, 1H), 4.45–4.30 (m, 3H), 3.70 (bs, 1H), 3.35 (s, 1H), 2.58 (bs, 1H), 2.54–2.43 (m, 1H), 2.18 (d, *J* = 12.4 Hz, 1H), 2.05 (s, 3H), 1.98–1.90 (m, 2H), 1.59 (s, 3H), 1.56 (bs, 1H), 1.30 (d, *J* = 11.5 Hz, 1H), 1.27–1.19 (m, 4H), 1.20–1.05 (m, 2H), 0.89 (d, *J* = 6.6 Hz, 3H), 0.80 (d, *J* = 9.9 Hz, 1H), 0.65–0.58 (m, 2H), 0.53 (s, 3H). ^13^C NMR (126 MHz, CDCl_3_) δ 202.87 (s), 166.82 (s), 153.44 (s), 152.69 (s), 131.97 (s), 128.28 (s), 127.37 (s), 125.43 (s),124.82 (s), 82.97 (s), 78.42 (s), 73.49 (s), 62.43 (s), 58.99 (s), 58.05 (s), 57.26 (s), 56.21 (s), 51.60 (s), 50.73 (s), 42.09 (s), 41.89 (s), 38.37 (s), 37.39 (s), 30.86 (s), 29.62 (s), 29.50 (s), 26.97 (s), 23.88 (s), 19.88 (s), 19.83 (s), 15.05 (s), 13.08 (s), 11.14 (s). HRMS ESI: m/z calcd. for C35H43BrNO7 (M + H)^+^ 668.2223, found 668.2256.

***4-fluorophenyl-2-isoxazoline withaferin A (W-4a)*** *.* White solid powder; ^1^H NMR (400 MHz, CDCl_3_) *δ* 7.81 (dd, *J* = 8.5, 5.4 Hz, 2H), 7.11 (t, *J* = 8.5 Hz, 2H),5.16 (dd, *J* = 11.9, 3.3 Hz, 1H), 4.61 (d, *J* = 11.9 Hz, 1H), 4.38–4.35 (m, 3H),), 3.70 (bs, 1H), 3.35 (s, 1H), 2.58 (bs, 1H), 2.54–2.43 (m, 1H), 2.18 (d, *J* = 12.4 Hz, 1H), 2.05 (s, 3H), 1.98–1.90 (m, 2H), 1.59 (s, 3H), 1.56 (bs, 1H), 1.30 (d, *J* = 11.5 Hz, 1H), 1.27–1.19 (m, 4H), 1.20–1.05 (m, 2H), 0.89 (d, *J* = 6.6 Hz, 3H), 0.80 (d, *J* = 9.9 Hz, 1H), 0.65–0.58 (m, 2H), 0.53 (s, 3H). ^13^C NMR (101 MHz, CDCl_3_) *δ* 203.48 (s), 179.85, 168.05 (d), 155.50 (s), 152.83 (s), 128.96(d), 127.06 (s), 125.72 (s), 116.69(d), 84.87 (s), 78.73 (s), 73.22 (s), 63.47 (s), 57.97 (s), 57.50 (s), 55.73 (s), 55.17 (s), 51.82 (s), 49.34 (s), 42.90 (s), 42.72 (s), 38.82 (s), 31.93 (s), 30.82 (s), 29.36 (s), 27.37 (s), 24.23 (s), 22.70 (s), 20.29 (s), 20.02 (s), 15.60 (s), 13.37 (s), 11.47 (s). HRMS ESI: m/z calcd. for C35H43FNO7 (M + H)^+^ 608.3024, found 608.3017.

***4-fluorophenyl-2-isoxazoline withaferin A (W-4b)*** *.* White solid powder; ^1^H NMR (400 MHz, CDCl_3_) *δ* 7.81 (dd, *J* = 8.5, 5.4 Hz, 2H), 7.11 (t, *J* = 8.5 Hz, 2H), 5.16 (dd, *J* = 11.9, 3.6 Hz, 1H), 4.61 (d, *J* = 11.9 Hz, 1H), 4.38–4.32 (m, 3H), 3.72 (d, *J* = 3.3 Hz, 1H), 3.36 (s, 1H), 2.89 (bs, 1H), 2.66 (bs, 1H), 2.52–2.41 (m, 1H), 2.19 (d, *J* = 11.0 Hz, 1H), 2.05 (s, 3H), 1.94 (d, *J* = 18.3 Hz, 2H), 1.29 (s, 3H), 1.27 (s, 3H), 1.25 (s, 3H), 0.87 (d, *J* = 6.8 Hz, 3H), 0.82 (d, *J* = 10.1 Hz, 2H), 0.73–0.58 (m, 2H), 0.53 (s, 3H). ^13^C NMR (101 MHz, CDCl_3_) *δ* 203.01 (s), 179.05, 167.40 (d), 155.09 (s), 152.08 (s), 128.50(d), 126.68(s), 125.25 (s), 116.22(d), 84.40 (s), 78.26 (s), 72.75 (s), 63.00 (s), 57.50 (s), 57.03 (s), 55.26 (s), 54.70 (s), 51.35 (s), 48.87 (s), 42.43 (s), 42.25(s), 38.35 (s), 31.47 (s), 30.35 (s), 28.90 (s), 26.90 (s), 22.23 (s), 20.02 (s), 19.82 (s), 19.55 (s), 15.13 (s), 12.90 (s), 11.00 (s). HRMS ESI: m/z calcd. for C35H43FNO7 (M + H)^+^ 608.3024, found 608.3058.

***pyridenyl-2-isoxazoline withaferin A (W-5a)*** *.* White solid powder; ^1^H NMR (400 MHz, CDCl_3_) *δ* 8.70 (s, 2H)H), 7.67 (d, *J* = 5.1 Hz, 2H), 5.24 (dd, *J* = 12.0, 3.5 Hz, 1H), 4.60 (d, *J* = 12.0 Hz, 1H), 4.41–4.32 (m, 3H), 3.73 (d, *J* = 3.5 Hz, 1H), 3.35 (s, 1H), 2.52–2.39 (m, 1H), 2.18 (d, *J* = 13.8 Hz, 1H), 2.04 (s, 3H), 1.91 (dd, *J* = 18.2, 2.9 Hz, 2H), 1.73 (bs, 1H), 1.63–1.53 (m, 3H), 1.29 (s, 3H), 1.25 (s, 3H), 1.20 (dd, *J* = 13.0, 8.0 Hz, 2H), 1.13–0.99 (m, 2H), 0.86 (d, *J* = 6.6 Hz, 3H), 0.82–0.76 (m, 1H), 0.67 (m, 1H), 0.53 (s, 3H). ^13^C NMR (101 MHz, CDCl_3_) *δ* 202.57 (s), 166.95 (s), 152.97 (s), 152.90 (s), 150.53 (s), 136.07 (s), 125.66 (s), 120.59 (s), 83.91 (s), 78.61 (s), 73.41 (s), 62.53 (s), 58.54 (s), 58.18 (s), 57.42 (s), 56.29 (s), 51.55 (s), 51.00 (s), 42.39 (s), 42.32 (s), 38.79 (s), 38.28 (s), 31.16 (s), 29.91 (s), 29.69 (s), 27.28 (s), 24.19 (s), 20.04 (s), 20.01 (s), 15.37 (s), 13.19 (s), 11.31 (s). HRMS ESI: m/z calcd. for C34H43N2O7 (M +H)H)^+^ 591.3070, found 591.3053.

***pyridenyl-2-isoxazoline withaferin A (W-5b)*** *.* White solid powder; ^1^H NMR (400 MHz, CDCl_3_) *δ* 8.75 (bs, 2H), 7.58 (bs, 2H), 5.04 (d, *J* = 10.8 Hz, 1H), 4.47–4.26 (m, 4H), 3.53 (s, 1H), 2.66 (s, 1H), 2.56–2.45 (m, 1H), 2.10 (d, *J* = 14.6 Hz, 1H), 2.05 (s, 3H), 2.02–1.95 (m, 3H), 1.65 (d, *J* = 6.3 Hz, 3H), 1.42 (s, 2H), 1.34 (s, 3H), 1.29 (s, 2H), 1.25 (s, 4H), 1.13 (d, *J* = 11.8 Hz, 2H), 1.00 (d, *J* = 6.6 Hz, 3H), 0.87 (d, *J* = 7.0 Hz, 1H), 0.67 (s, 3H). ^13^C NMR (101 MHz, CDCl_3_) *δ* 202.83(s), 167.12 (s), 155.05 (s), 153.09 (s), 150.91 (s), 135.15 (s), 125.70 (s), 123.48 (s), 85.47 (s), 78.73 (s), 72.84 (s), 63.43 (s), 57.86 (s), 57.46 (s), 55.69 (s), 54.19 (s), 51.75 (s), 49.39 (s), 42.91 (s), 42.69 (s), 38.77 (s), 31.64 (s), 30.77 (s), 29.79 (s), 27.35 (s), 24.21 (s), 22.73 (s), 20.26 (s), 20.10 (s), 15.65 (s), 13.39 (s), 11.49 (s). HRMS ESI: m/z calcd. for C34H43N2O7 (M + H)^+^ 591.3070, found 591.3079.

***2,6-dichlorophenyl-2-isoxazoline withaferin A (W-6a)*** *.* White solid powder; ^1^H NMR (400 MHz, CDCl_3_) *δ* 7.51 (d, *J* = 8.4 Hz, 1H), 7.46 (d, *J* = 1.9 Hz, 1H), 7.28 (d, *J* = 11.2 Hz, 1H), 4.96 (d, *J* = 11.2 Hz, 1H), 4.64 (dd, *J* = 11.2, 2.0 Hz, 1H), 4.37–4.30 (m, 2H), 4.05 (q, *J* = 7.1 Hz, 2H), 3.25 (bs, 1H), 2.81 (bs, 1H), 2.43–2.39 (m, 1H), 2.08 (d, *J* = 9.3 Hz, 1H), 1.98 (s, 3H), 1.53 (s, 3H), 1.37–1.30 (m, 3H), 1.25 (s, 3H), 1.19 (d, *J* = 2.3 Hz, 4H), 1.11–1.04 (m, 1H), 0.93 (d, *J* = 6.6 Hz, 2H), 0.83–0.79 (m, 2H), 0.60 (s, 3H). ^13^C NMR (126 MHz, CDCl_3_) *δ* 201.16 (s), 165.14, 152.94, 151.01, 135.40, 131.66, 129.57, 129.44, 126.05, 123.69, 123.07, 82.69, 76.78, 61.61, 58.51, 56.14, 55.55, 53.89, 53.79, 59.82, 47.46, 40.95, 40.95, 40.75, 36.84, 28.92, 27.63, 25.41, 22.28, 18.36, 18.13, 18.09, 13.68, 12.27, 11.43, 9.52. HRMS ESI: m/z calcd. for C35H42Cl2NO7 (M + H)^+^ 658.2338, found 658.2338.

***2,6-dichlorophenyl-2-isoxazoline withaferin A (W-6b)*** *.* White solid powder; ^1^H NMR (400 MHz, CDCl_3_) *δ* 7.55 (d, *J* = 8.2 Hz, 2H), 7.34 (d, *J* = 8.3 Hz, 1H), 5.17 (dd, *J* = 12.0, 3.4 Hz, 1H), 4.82 (d, *J* = 12.0 Hz, 1H), 4.82 (d, *J* = 12.0 Hz, 1H), 4.41–4.38 (m, 3H), 3.72 (bs, 2H), 3.37 (s, 1 H), 2.90 (t, *J* = 6.5 Hz, 1H), 2.69 (bs, 1 H), 2.57–2.45 (m, 1H), 2.20 (d, *J* = 13.9 Hz, 1H), 2.09 (s, 3H), 1.96 (d, *J* = 21.5 Hz, 1H), 1.61 (s, 7H), 1.48–1.38 (m, 1H), 1.36–1.31 (m, 1H), 1.25 (s, 3H), 1.12 (s, 2H), 0.91 (d, *J* = 6.3 Hz, 3H), 0.75 (bs, 1H), 0.55 (s, 3H). ^**13**^ **C** NMR (126 MHz, CDCl_3_) *δ* 201.54 (s), 166.28 (s), 152.17 (s), 151.34 (s), 135.86 (s), 132.89 (s), 130.96 (s), 130.55 (s), 126.66 (s), 124.88 (s), 124.16 (s), 81.38 (s), 77.93 (s), 72.54 (s), 62.02 (s), 59.04 (s), 57.66 (s), 56.72 (s), 54.77 (s), 51.17 (s), 49.94 (s), 41.59 (s), 40.89 (s), 37.81 (s), 37.53 (s), 30.28 (s), 29.04 (s), 28.89 (s), 26.45 (s), 23.50 (s), 19.34 (s), 19.26 (s), 14.61 (s), 12.63 (s), 10.46 (s). HRMS ESI: m/z calcd. for C35H42Cl2NO7 (M + H)^+^ 658.2338, found 658.2396.

***3-bromo-6-methoxyphenyl-2-isoxazoline withaferin A (*** **W-7a** ***)*** *.* White solid powder; ^1^H NMR (400 MHz, CDCl_3_) *δ* 7.66 (d, *J* = 2.4 Hz, 1H), 7.43 (dd, *J* = 8.9, 2.4 Hz, 1H), 6.80 (d, *J* = 8.9 Hz, 1H), 5.25 (d, *J* = 14.8 Hz, 1H), 5.07 (dd, *J* = 12.2, 3.2 Hz, 1H), 4.95 (d, *J* = 12.2 Hz, 1 H), 4.82 (q, *J* = 11.9 Hz, 2H), 4.71 (d, *J* = 3.2 Hz, 1H), 4.33 (d, *J* = 13.1 Hz, 1H), 3.84 (s, 3H), 3.35 (s, 1H), 2.86 (s, 1H), 2.58 (s, 1H), 2.53–2.42 (m, 1H), 2.18 (d, *J* = 12.4 Hz, 1H), 2.05 (d, *J* = 4.8 Hz, 3H), 1.94 (dd, *J* = 25.4, 7.2 Hz, 2H), 1.59 (s, 5H), 1.25 (s, 3H), 1.17–0.99 (m, 2H), 0.89 (d, *J* = 6.6 Hz, 3H), 0.80 (d, *J* = 9.9 Hz, 1H), 0.61 (s, 2 H), 0.53 (s, 3H). ^13^C NMR (101 MHz, CDCl_3_) *δ* 201.80 (s), 165.26 (s), 156.99 (s), 156.88 (s), 153.23 (s), 134.52 (s), 133.29 (s), 121.94 (s), 118.89 (s), 113.92 (s), 112.74 (s), 80.53 (s), 78.09 (s), 74.40 (s), 59.79 (s), 59.71 (s), 58.20 (s), 58.02 (s), 56.54 (s), 56.03 (s), 51.76 (s), 51.21 (s), 42.27 (s), 41.57 (s), 38.57 (s), 38.07 (s), 31.04 (s), 30.00 (s), 29.69 (s), 27.22 (s), 24.07 (s), 20.02 (s), 14.34 (s), 13.26 (s), 11.34 (s). HRMS ESI: m/z calcd. C36H45BrNO8 (M + H)^+^ 698.2329, found 698.2317.

***2-trifluoromethylphenyl-2-isoxazoline withaferin A (*** **W-8a** ***)*** *.* White solid powder; ^1^H NMR (400 MHz, CDCl_3_) *δ* 7.83–7.79 (m, 1H), 7.62 (dd, *J* = 8.6, 6.5 Hz, 2H), 7.56 (d, *J* = 8.3 Hz, 1H), 5.00 (d, *J* = 10.6 Hz, 1H), 4.41 (dt, *J* = 13.2, 3.4 Hz, 1H), 4.37–4.30 (m, 2H), 4.10 (dd, *J* = 14.3, 7.1 Hz, 1H), 3.23 (bs, 1H), 2.95 (s, 1H), 2.55–2.43 (m, 1H), 2.23 (s, 1H), 2.17 (d, *J* = 8.9 Hz, 1H), 2.03 (s, 3H), 1.99 (d, *J* = 3.3 Hz, 1H), 1.95 (d, *J* = 3.2 Hz, 1H), 1.73–1.63 (m, 3H), 1.55 (bs, 3H), 1.43–1.37 (m, 2H), 1.32 (s, 3H), 1.23 (s, 3H), 1.15–1.07 (m, 2H), 0.99 (d, *J* = 6.6 Hz, 3H), 0.88–0.83 (m, 1H), 0.66 (s, 3H). ^13^C NMR (101 MHz, CDCl_3_) *δ* 203.63 (s), 167.22 (s), 156.39 (s), 153.03 (s), 132.62 (s), 131.68 (s), 130.97 (s), 129.06 (s), 127.80 (d, *J* = 5.8 Hz), 126.34 (s), 125.93 (s), 125.23 (s), 85.10 (s), 78.95 (s), 72.60 (s), 63.82 (s), 58.62 (s), 57.73 (s), 57.29 (s), 55.98 (s), 52.03 (s), 49.60 (s), 42.95 (s), 42.83 (s), 39.04 (s), 30.98 (s), 30.04 (s), 29.91 (s), 29.80 (s), 27.60 (s), 24.46 (s), 20.56 (s), 20.22 (s), 15.79 (s), 13.57 (s), 11.70 (s). HRMS ESI: m/z calcd. for C36H43F3NO7 (M + H)^+^ 658.2992, found 658.2973.

***2-methoxy-4-trifluorophenyl-2-isoxazoline withaferin A (*** **W-9b** ***)*** *.* White solid powder; ^1^H NMR (400 MHz, CDCl_3_) *δ* 7.81 (d, *J* = 8.1 Hz, 1H), 7.23 (d, *J* = 6.1 Hz, 2H), 5.13 (dd, *J* = 12.2, 3.2 Hz, 1H), 4.96 (d, *J* = 12.2 Hz, 1H), 4.35 (q, *J* = 12.3 Hz, 3H), 3.92 (s, 3H), 3.60 (d, *J* = 3.1 Hz, 1H), 3.32 (s, 1H), 2.44 (dd, *J* = 17.3, 13.8 Hz, 1H), 2.28 (bs, 1H), 2.04 (s, 3H), 1.92 (dd, *J* = 18.2, 2.7 Hz, 2H), 1.39 (s, 3H), 1.26 (bs, 6H), 1.23 (s, 3H), 1.19 (s, 3H), 0.87 (s, 1H), 0.85 (s, 2H), 0.83 (d, *J* = 6.4 Hz, 3H), 0.66–0.60 (m, 1H), 0.49 (s, 3H). ^13^C NMR (101 MHz, CDCl_3_) *δ* 202.46 (s), 167.17 (s), 157.82 (s), 153.12 (s), 152.99 (s), 133.63 (s), 131.06 (s), 125.86 (s), 121.35 (s), 117.60 (d, *J* = 3.8 Hz), 114.24 (s), 108.97 (d, *J* = 3.8 Hz), 83.38 (s), 78.74 (s), 73.93 (s), 63.00 (s), 59.95 (s), 58.40 (s), 57.60 (s), 56.54 (s), 56.16 (s), 52.13 (s), 50.84 (s), 42.47 (s), 41.49 (s), 38.83 (s), 38.30 (s), 32.10 (s), 31.33 (s), 27.40 (s), 24.27 (s), 22.87 (s), 20.15 (s), 15.32 (s), 14.29 (s), 13.22 (s), 11.39 (s). HRMS ESI**:** m/z calcd. for C37H45F3NO8 (M + H)^+^ 688.3097, found 688.3099.

***2,6-dichlorophenyl-2-isoxazoline withaferin A (*** **W-10b** ***)*** *.* White solid powder; ^1^H NMR (400 MHz, CDCl_3_) *δ* 7.42 (t, *J* = 7.0 Hz, 2H), 7.36–7.32 (m, 1H), 5.14 (d, *J* = 10.0 Hz, 1H), 4.46–4.44 (m, 1H), 4.40–4.33 (m, 3H), 3.48 (s, 1H), 3.46 (s, 1H), 2.57–2.46 (m, 1H), 2.18 (d, *J* = 11.2 Hz, 1H), 2.05 (s, 3H), 2.03–1.94 (m, 3H), 1.67 (s, 5H), 1.36 (bs, 5H), 1.25 (s, 3H), 1.15 (d, *J* = 9.7 Hz, 2H), 1.01 (d, *J* = 6.6 Hz, 3H)H), 0.93–0.85 (m, 2H), 0.69 (s, 3H). ^13^C NMR (126 MHz, CDCl_3_) *δ* 202.68 (s), 165.42 (s), 152.86 (s), 151.41 (s), 133.34 (s), 129.84 (s), 127.25 (s), 124.86 (s), 123.88 (s), 82.63 (s), 77.04 (s), 68.99 (s), 62.58 (s), 57.26 (s), 55.75 (s), 55.18 (s), 54.04 (s), 51.78 (s), 50.09 (s), 47.61 (s), 41.01 (s), 39.84 (s), 37.12 (s), 37.08 (s), 28.75 (s), 28.06 (s), 27.39 (s), 25.61 (s), 22.51 (s), 18.88 (s), 18.37 (s), 13.89 (s), 11.67 (s). HRMS ESI: m/z calcd. for C35H42Cl2NO7 (M +H)^+^ 658.2338, found 658.2339.

***4-trifluorophenyl-2-isoxazoline withaferin A (*** **W-11a** ***)*** *.* White solid powder; ^1^H NMR (400 MHz, CDCl_3_) *δ* 7.79 (d, *J* = 8.2 Hz, 2H), 7.70 (d, *J* = 8.3 Hz, 2H), 5.00 (d, *J* = 10.7 Hz, 1H), 4.43–4.28 (m, 4H), 3.50 (s, 1H), 2.65 (s, 1H), 2.54–2.45 (m, 1H), 2.12–2.07 (m, 1H), 2.03 (s, 3H), 2.00–1.92 (m, 3H), 1.66 (dd, *J* = 16.8, 5.9 Hz, 4H), 1.31 (s, 3H), 1.24 (s, 5H), 1.16–1.06 (m, 3H), 0.99 (d, *J* = 6.6 Hz, 3H), 0.85 (dd, *J* = 16.2, 9.3 Hz, 2H), 0.65 (s, 3H). ^13^C NMR (101 MHz, CDCl_3_) *δ* 203.20 (s), 167.36 (s), 153.79 (s), 153.22 (s), 132.74 (s), 132.42 (s), 127.74 (s), 126.32 (dd, *J* = 7.4, 3.6 Hz), 126.10 (s), 122.75 (s), 84.01 (s), 78.95 (s), 74.16 (s), 62.87 (s), 59.51 (s), 58.56 (s), 57.87 (s), 57.07 (s), 52.23 (s), 51.25 (s), 42.67 (s), 42.39 (s), 39.03 (s), 38.53 (s), 31.47 (s), 30.25 (s), 29.94 (s), 27.61 (s), 24.44 (s), 20.36 (s), 20.34 (s), 15.64 (s), 13.42 (s), 11.72 (s). HRMS ESI: m/z calcd. for C36H43F3NO7 (M + H)^+^ 658.2992, found 658.2984.

***4-trifluorophenyl-2-isoxazoline withaferin A (*** **W-11b** ***)*** *.* White solid powder; ^1^H NMR (400 MHz, CDCl_3_) *δ* 7.89 (d, *J* = 8.2 Hz, 2H), 7.66 (d, *J* = 8.3 Hz, 2H), 5.20 (dd, *J* = 12.0, 3.3 Hz, 1H), 4.63 (d, *J* = 12.0 Hz, 1H), 4.42–4.25 (m, 3H), 3.68 (d, *J* = 3.3 Hz, 1H), 3.33 (s, 1H), 2.47–2.34 (m, 1H), 2.16 (d, *J* = 12.9 Hz, 1H), 2.03 (s, 3H), 1.88 (dd, *J* = 17.9, 3.2 Hz, 2H), 1.57–1.48 (m, 4H), 1.22 (s, 3H), 1.23 (s, 3H), 1.19 (d, *J* = 12.9 Hz, 2H), 1.15–0.97 (m, 3H), 0.86 (s, 1H), 0.80 (d, *J* = 6.6 Hz, 3H), 0.68 (dd, *J* = 19.6, 9.6 Hz, 1H), 0.59 (dd, *J* = 14.3, 3.4 Hz, 1H), 0.49 (s, 3H). ^13^C NMR (101 MHz, CDCl_3_) *δ* 203.00 (s), 167.16 (s), 153.59 (s), 153.02 (s), 132.55 (s), 132.22 (s), 127.54 (s), 126.12 (dd, *J* = 7.4, 3.6 Hz), 125.90 (s), 122.55 (s), 83.81 (s), 78.75 (s), 73.96 (s), 62.67 (s), 59.31 (s), 58.37 (s), 57.67 (s), 56.87 (s), 52.03 (s), 51.05 (s), 42.47 (s), 42.19 (s), 38.83 (s), 38.34 (s), 31.27 (s), 30.06 (s), 29.91 (s), 29.74 (s), 27.41 (s), 24.24 (s), 20.17 (s), 15.44 (s), 13.22 (s), 11.52 (s). HRMS ESI: m/z calcd. for C36H43F3NO7 (M + H)^+^ 658.2992, found 658.2981.

***4-fluorophenyl-2-isoxazoline 4,27-acetylwithaferin A (*** **W-12b** ***)*** *.* White solid powder; ^1^H NMR (400 MHz, CDCl_3_) *δ* 7.78 (dd, *J* = 8.6, 5.3 Hz, 2H), 7.13 (t, *J* = 8.5 Hz, 2H), 5.17 (dd, *J* = 11.9, 3.2 Hz, 1H), 4.93–4.83 (m, 2H), 4.79 (d, *J* = 3.2 Hz, 1H), 4.58 (d, *J* = 11.9 Hz, 1H), 4.35 (bd, *J* = 13.1 Hz, 1H), 3.43 (s, 1H), 2.77 (s, 1H), 2.57–2.42 (m, 1H), 2.19 (d, *J* = 12.0 Hz, 1H), 2.12 (s, 3H), 2.09 (s, 3H), 2.06 (s, 3H), 2.00–1.91 (m, 2H), 1.58 (dd, *J* = 17.7, 5.3 Hz, 4H), 1.29 (s, 2H), 1.25 (s, 4H), 1.25 (s, 3H), 1.10–1.05 (m, 1H), 0.87 (d, *J* = 6.7 Hz, 3H), 0.70–0.62 (m, 1H), 0.53 (s, 3H). ^13^C NMR (126 MHz, CDCl_3_) *δ* 202.78 (s), 170.96 (s), 169.12 (s), 165.28 (s), 163.04 (s), 157.03 (s), 153.25 (s), 129.06 (d, *J* = 8.5 Hz), 124.59 (d, *J* = 3.4 Hz), 121.85 (s), 116.21 (d, *J* = 22.0 Hz), 80.59 (s), 78.10 (s), 74.37 (s), 59.75 (s), 59.65 (s), 58.22 (s), 58.01 (s), 56.14 (s), 51.78 (s), 51.60 (s), 42.33 (s), 42.15 (s), 38.57 (s), 38.23 (s), 31.10 (s), 27.21 (s), 24.13 (s), 20.96 (s), 20.93 (s), 20.64 (s), 20.02 (s), 14.52 (s), 13.29 (s), 11.37 (s).). HRMS ESI: m/z calcd. for C41H49F3NO10 (M + H)^+^ 772.3309, found 772.3343.

## Electronic supplementary material

Supporting information
