# Supplementary material for: Regiospecific Synthesis of Ring A Fused Withaferin A Isoxazoline Analogues: Induction of Premature Senescence by W-2b in Proliferating Cancer Cells
Source: Sci Rep. 2017 Oct 23;7:13749. doi: 10.1038/s41598-017-13664-x (PMC5653814; doi:10.1038/s41598-017-13664-x)
Supplement: Supplementary file 1 — Supporting information [file 41598_2017_13664_MOESM1_ESM.pdf]

Supporting Information

Regiospecific Synthesis of Ring A Fused Withaferin A Isoxazoline Analogues: Induction of Premature Senescence by W-2b in Proliferating Cancer Cells

Faheem Rasool,[1,2,#] Debasis Nayak,[1,3,#] Archana Katoch,[1,3] Mir Mohd Faheem,[1,3] Syed Khalid Yousuf,[1,4] Nazar Hussain,[1,2] Chetan Belawal,[5] N. K. Satti,[5] Anindya Goswami,[1,3,*] Debaraj Mukherjee[1,2,*]

[1]Academy of Scientific and Innovative Research (AcSIR), New Delhi, India.

[2]Natural Product Chemistry-Microbes, CSIR-Indian Institute of Integrative Medicine, Jammu-180001, India.

[3]Cancer Pharmacology Division, CSIR-Indian Institute of Integrative Medicine, Jammu-180001, India.

[4]Medicinal Chemistry Division, CSIR-Indian Institute of Integrative Medicine, Srinagar-190005, India.

[5]Natural Product Chemistry-Plants, CSIR-Indian Institute of Integrative Medicine, Jammu-180001, India.

Table of Contents:

Sl. No.	Contents	Page No.
1	Supporting figures	S2-S3
2	HPLC of W-2b	S4
3	HRMS OF W-2b	S5
4	Copies of ^{1}H NMR, ^{13}C NMR	S6-S43
5	2D NMR	S44-S51

1. Supplementary figures

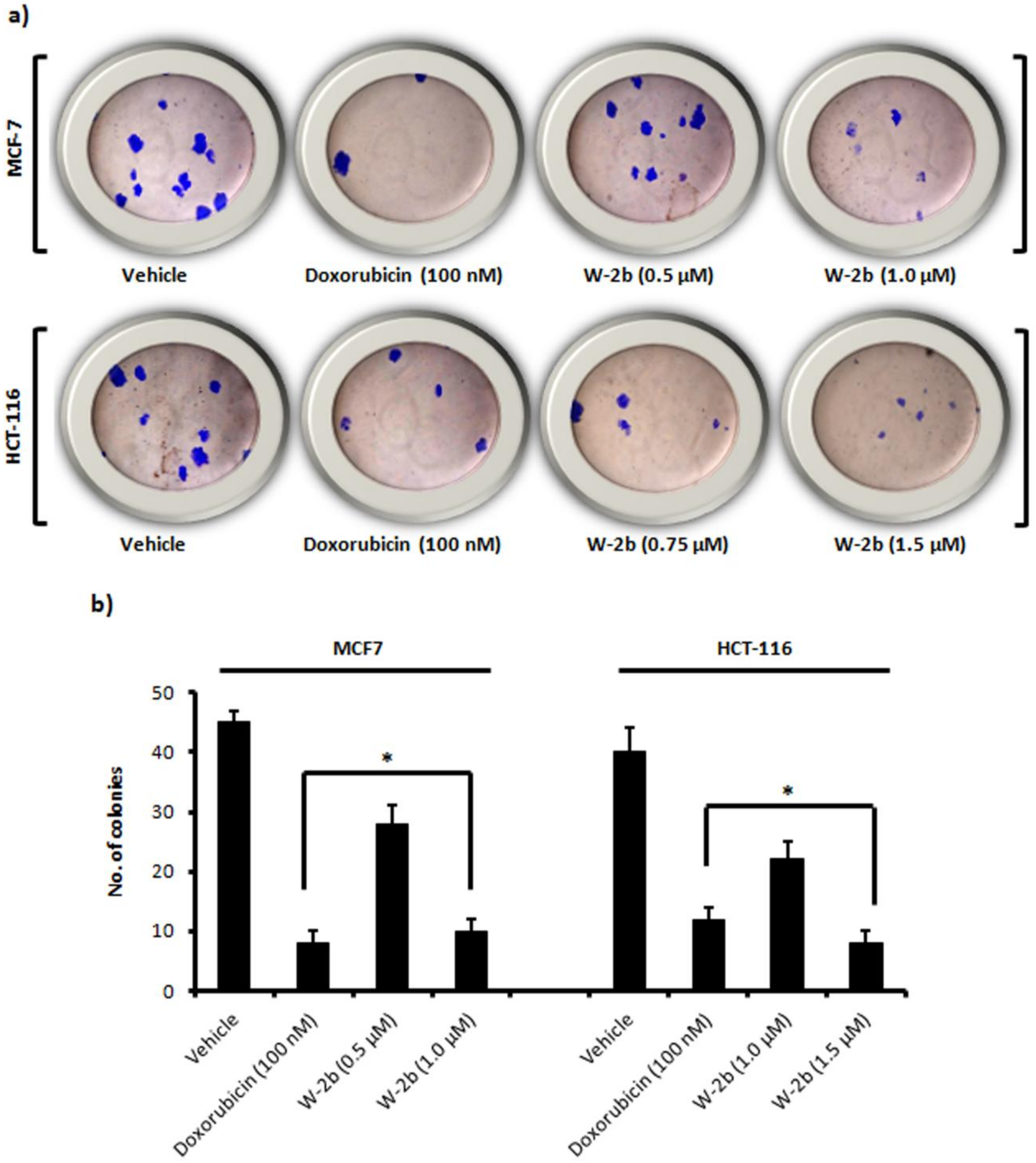

Supplementary Figure S1. W-2b inhibits cell proliferation. (a) Effect of vehicle, doxorubicin and increasing concentrations of **W-2b** on colony formation ability of MCF7 and HCT-116 cells. Original magnification 20x. (b) Bar graph showing the quantification of number of colonies in vehicle and each treatment conditions. Error bars: mean ± s.d., $*P < 0.05$.

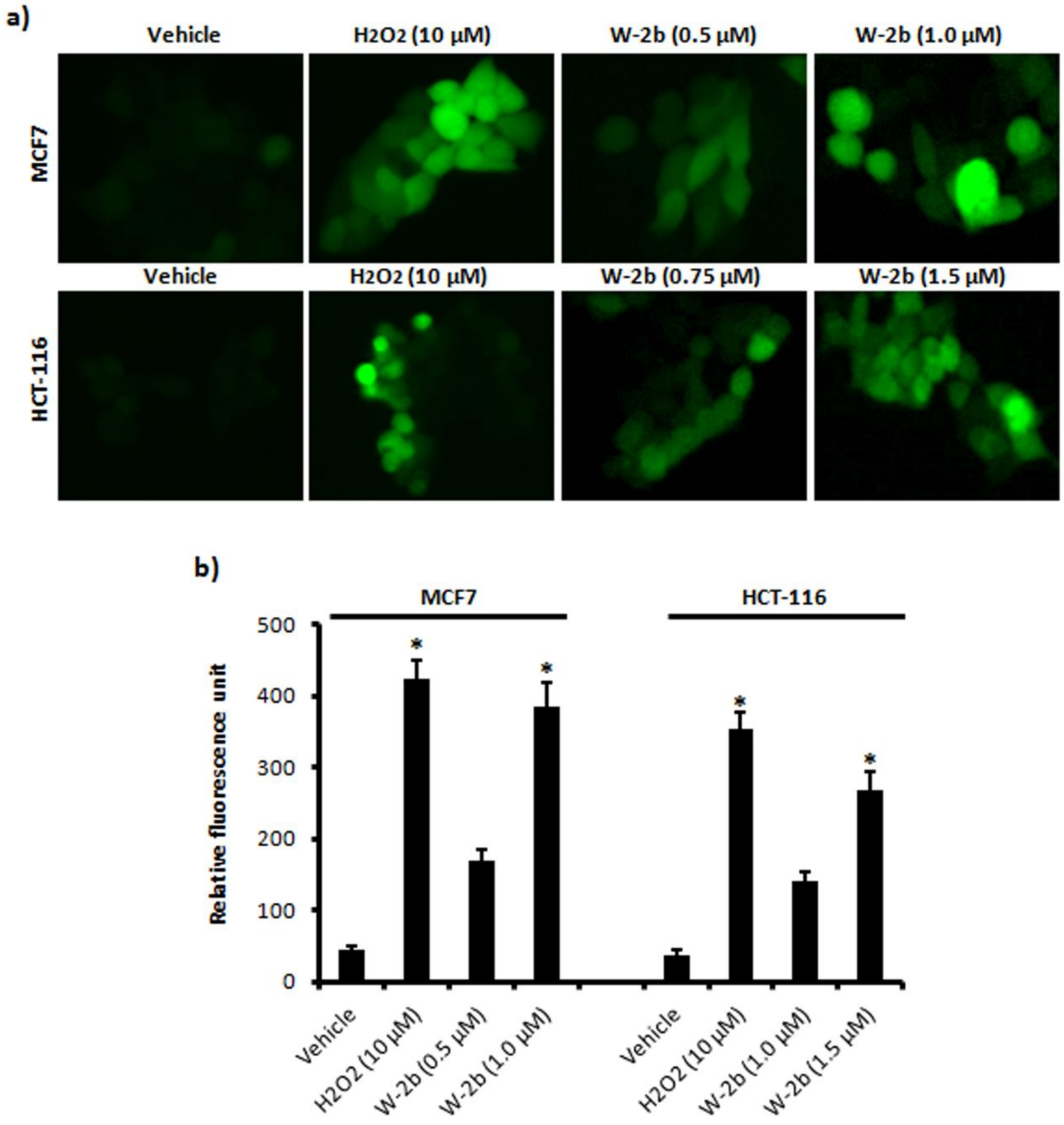

Supplementary Figure S2. Induction of ROS generation by **W-2b**. (a) Effects of vehicle, H_2O_2 (10 µM) and increasing concentrations of **W-2b** on ROS generation in MCF7 and HCT-116 cells. Original magnification 20x. (b) Bar graph showing the quantification of ROS generation in terms of relative fluorescence units (RFU) in each case. Error bars: mean ± s.d. of three independent experiments. *$P < 0.05$.

HPLC of W-2b

NPC DIVISION IIIM JAMMU

Reported by User: System

Project Name: NP

SAMPLE INFORMATION

Sample Name:	W-2a	Acquired By:	System
		Date Acquired:	5/14/2015 5:03:38 PM
Sample Type:	Unknown	Acq. Method Set:	Withenia
Vial:	5	Date Processed:	5/15/2015 4:07:11 PM
Injection #:	1	Processing Method:	Withaferin A
Injection Volume:	5.00 ul	Channel Name:	W2996 237.0nm-1.2
Run Time:	60.0 Minutes	Proc. Chnl. Descr.:	W2996 PDA 237.0 nm at 1.2
Sample Set Name:	Withania	Flow rate: 0.70 ml/min	
column_name	RP-18,5um	Sample conc: 1 mg/ml	
		Mobile phase: ACN:Water(gradient)	

Auto-Scaled Chromatogram

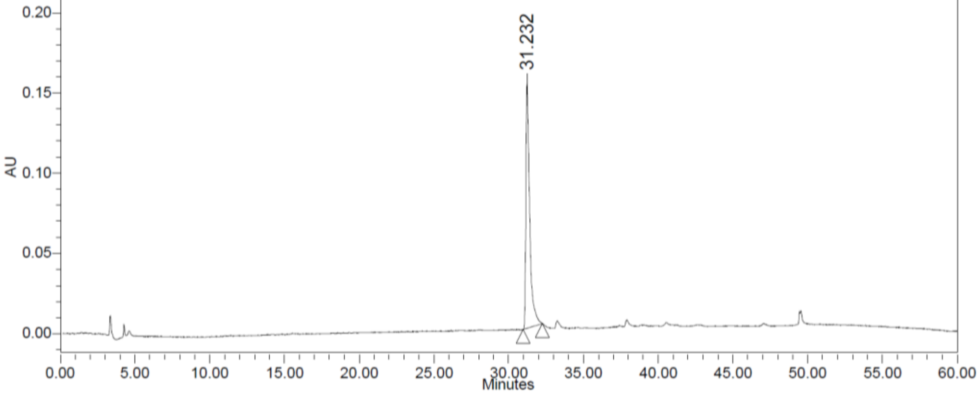

	RT	Area (μV*sec)	% Area	Height (μV)
1	31.232	2717828	100.00	152379

HRMS of W-2b

Qualitative Compound Report

**Data File**	W-2b	**Sample Name**	W-2b
**Sample Type**	Sample	**Position**	Vial 7
**Instrument Name**	Instrument 1	**User Name**	
**Acq Method**	vishal_12-01-13.m	**Acquired Time**	17-06-2015 PM 12:52:38
**IRM Calibration Status**	Success	**DA Method**	daily_report.m
**Comment**			

**Sample Group**		**Info.**
**Acquisition SW Version**	6200 series TOF/6500 series Q-TOF B.05.01 (B5125)	

Compound Table

Compound Label	RT	Mass	Formula	MFG Formula	MFG Diff (ppm)	DB Formula
Cpd 7: C35 H42 N2 O9	0.265	634.2888	C35 H42 N2 O9	C35 H42 N2 O9	0.36	C35 H42 N2 O9

Compound Label	*m/z*	RT	Algorithm	Mass
Cpd 7: C35 H42 N2 O9	635.2961	0.265	Find by Molecular Feature	634.2888

MFE MS Spectrum

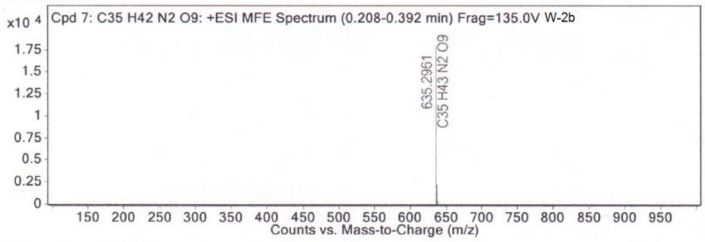

MS Spectrum Peak List

m/z	z	Abund	Formula	Ion
635.2961	1	17890.38	C35 H43 N2 O9	(M+H)+
636.2992	1	6736.49	C35 H43 N2 O9	(M+H)+
637.3036	1	2241.15	C35 H43 N2 O9	(M+H)+
638.3013	1	443.86	C35 H43 N2 O9	(M+H)+

Predicted Isotope Match Table

Isotope	*m/z*	Calc m/z	Diff (ppm)	Abund %	Calc Abund %	Abund Sum %	Calc Abund Sum %
1	635.2961	635.2963	0.38	100	100	65.5	66.44
2	636.2992	636.2996	0.66	37.65	39.42	24.67	26.19
3	637.3036	637.3024	-1.82	12.53	9.41	8.21	6.26
4	638.3013	638.3051	6.06	2.48	1.67	1.63	1.11

--- End Of Report ---

^{1}H NMR of W-1a

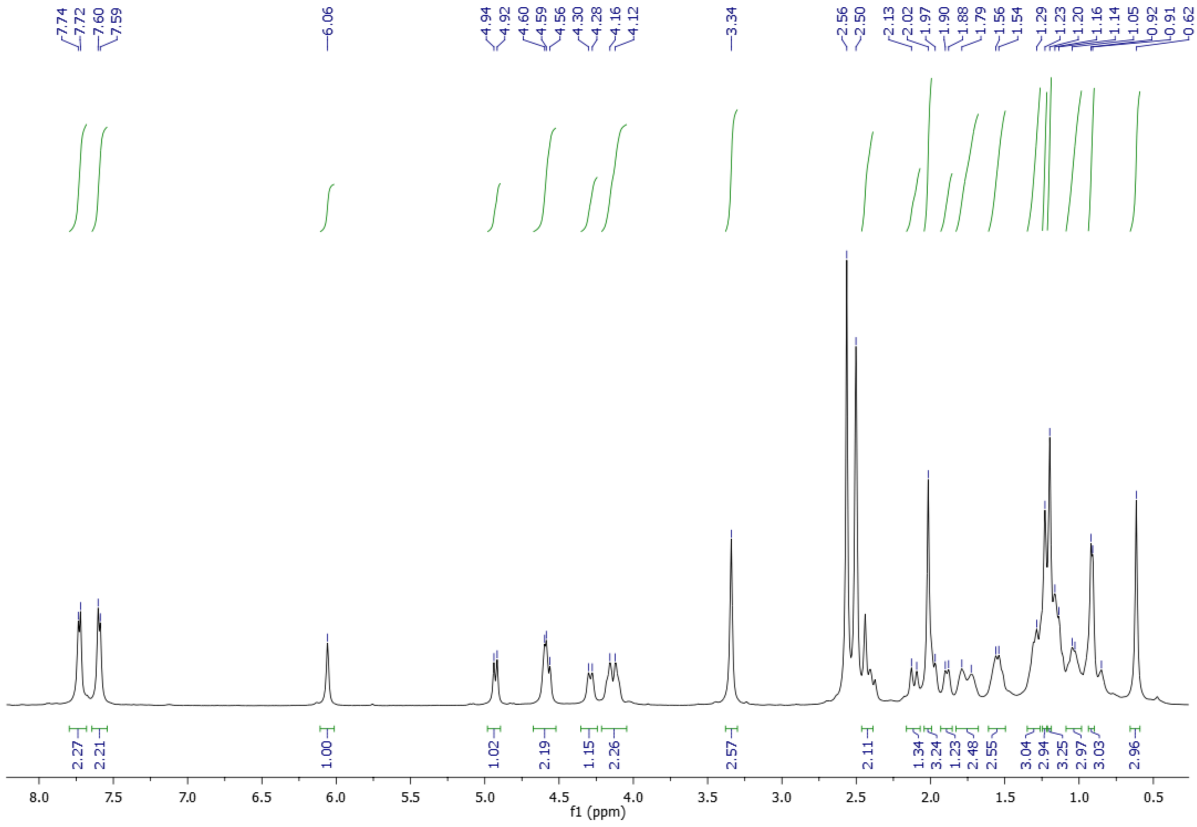
7.74
7.72
7.60
7.59
6.06
4.94
4.92
4.60
4.59
4.56
4.30
4.28
4.16
4.12
3.34
2.56
2.50
2.13
2.02
1.97
1.90
1.88
1.79
1.56
1.54
1.29
1.23
1.20
1.16
1.14
1.05
0.92
0.91
0.62
2.27
2.21
1.00
1.02
2.19
1.15
2.26
2.57
2.11
1.34
3.24
1.23
2.48
2.55
3.04
2.94
3.25
2.97
3.03
2.96
8.0
7.5
7.0
6.5
6.0
5.5
5.0
4.5
4.0
3.5
3.0
2.5
2.0
1.5
1.0
0.5
f1 (ppm)

^{13}C NMR of W-1a

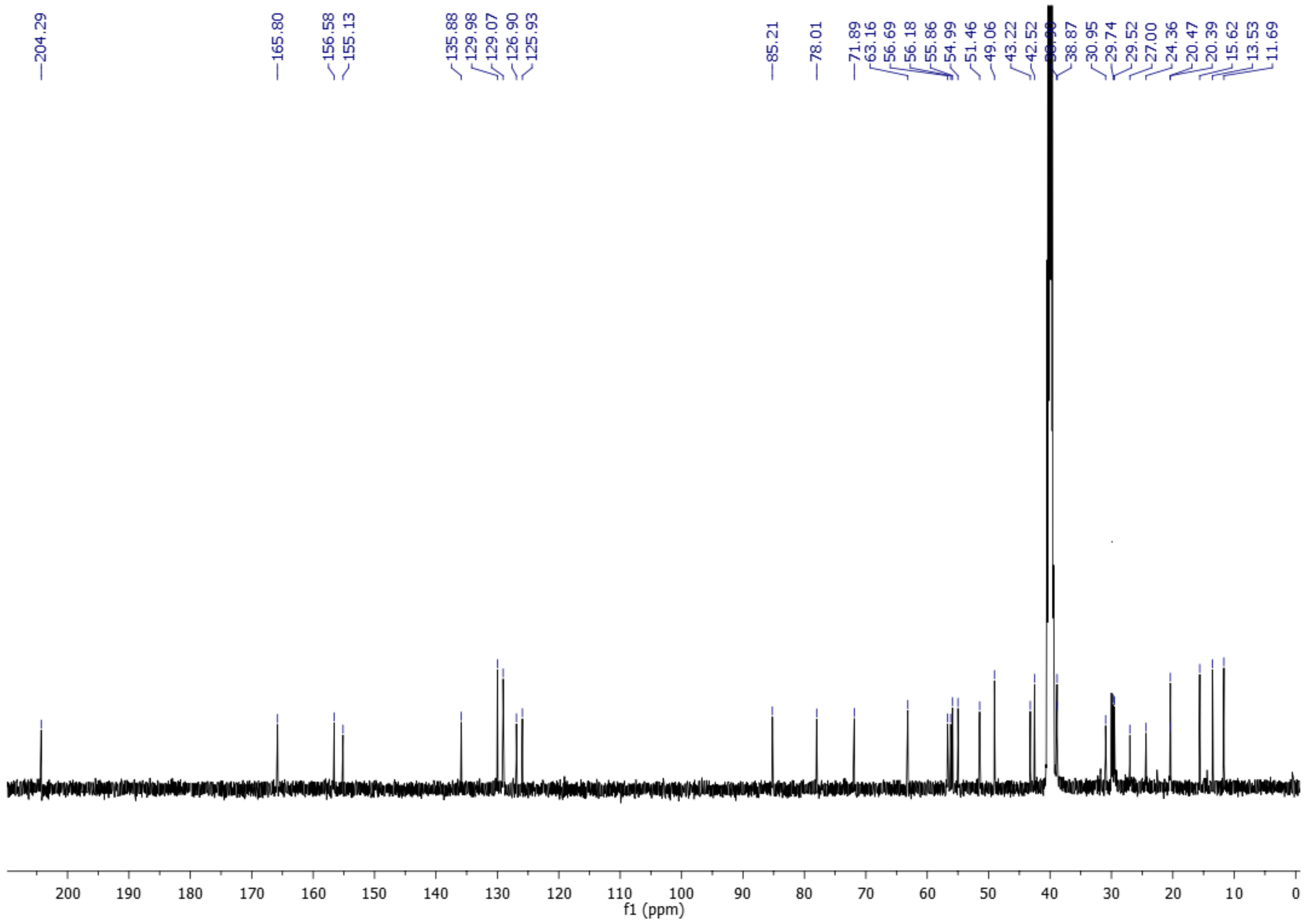

^{1}H NMR of W-1b

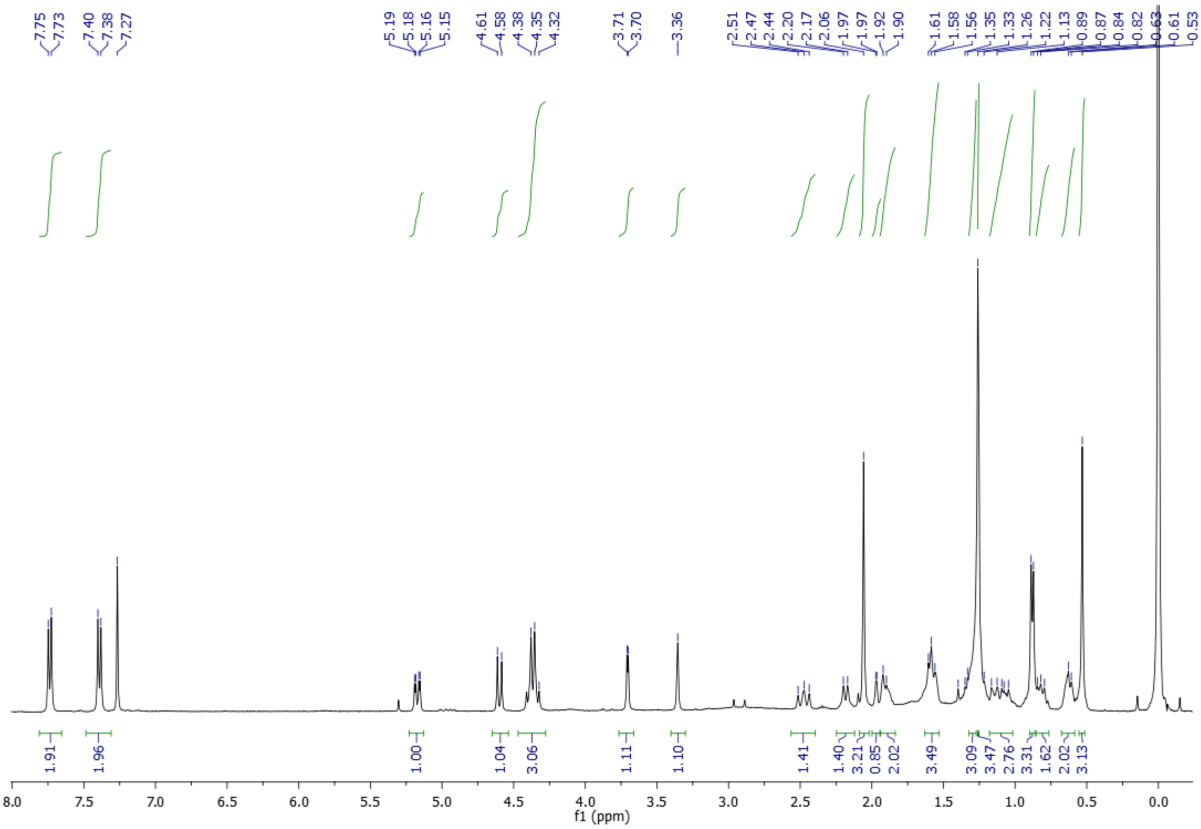
7.75
7.73
7.40
7.38
7.27
5.19
5.18
5.16
5.15
4.61
4.58
4.38
4.35
4.32
3.71
3.70
3.36
2.51
2.47
2.44
2.20
2.17
2.06
1.97
1.97
1.92
1.90
1.61
1.58
1.56
1.35
1.33
1.26
1.22
1.13
0.89
0.87
0.84
0.82
0.63
0.61
0.53
1.91
1.96
1.00
1.04
3.06
1.11
1.10
1.41
1.40
3.21
0.85
2.02
3.49
3.09
3.47
2.76
3.31
1.62
2.02
3.13
f1 (ppm)

^{13}C NMR of W-1b

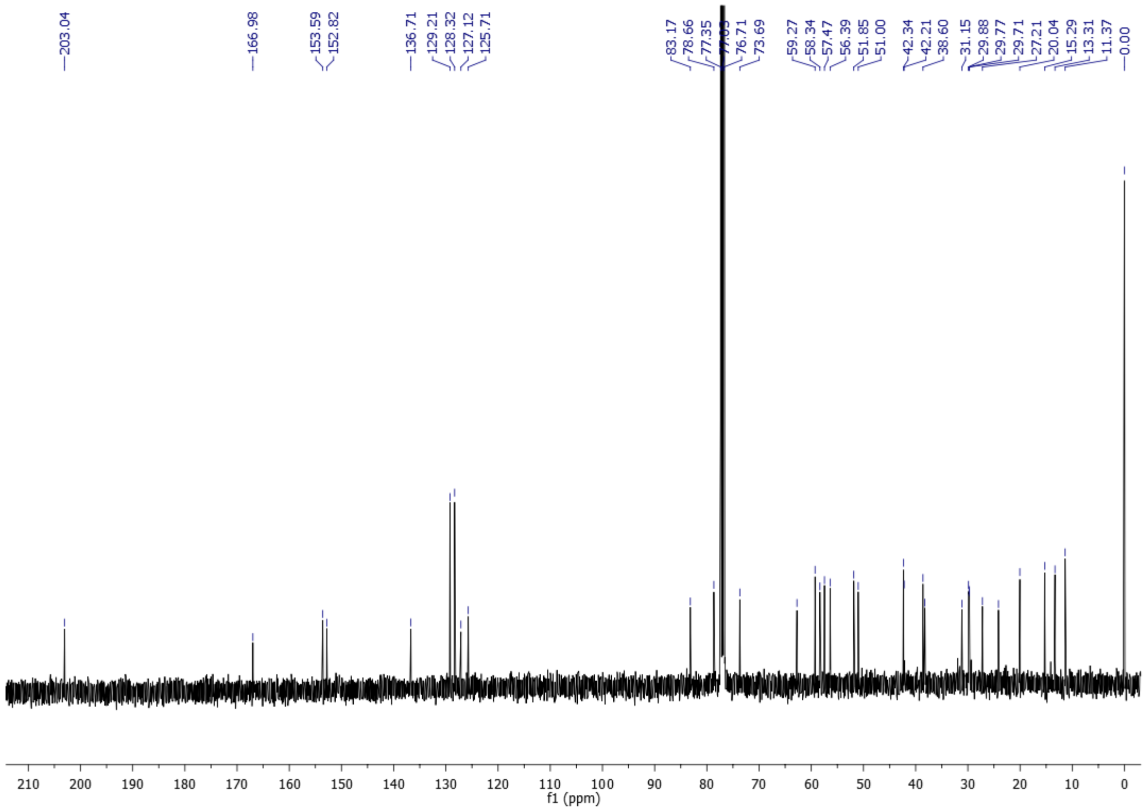

^{1}H NMR of W-2a

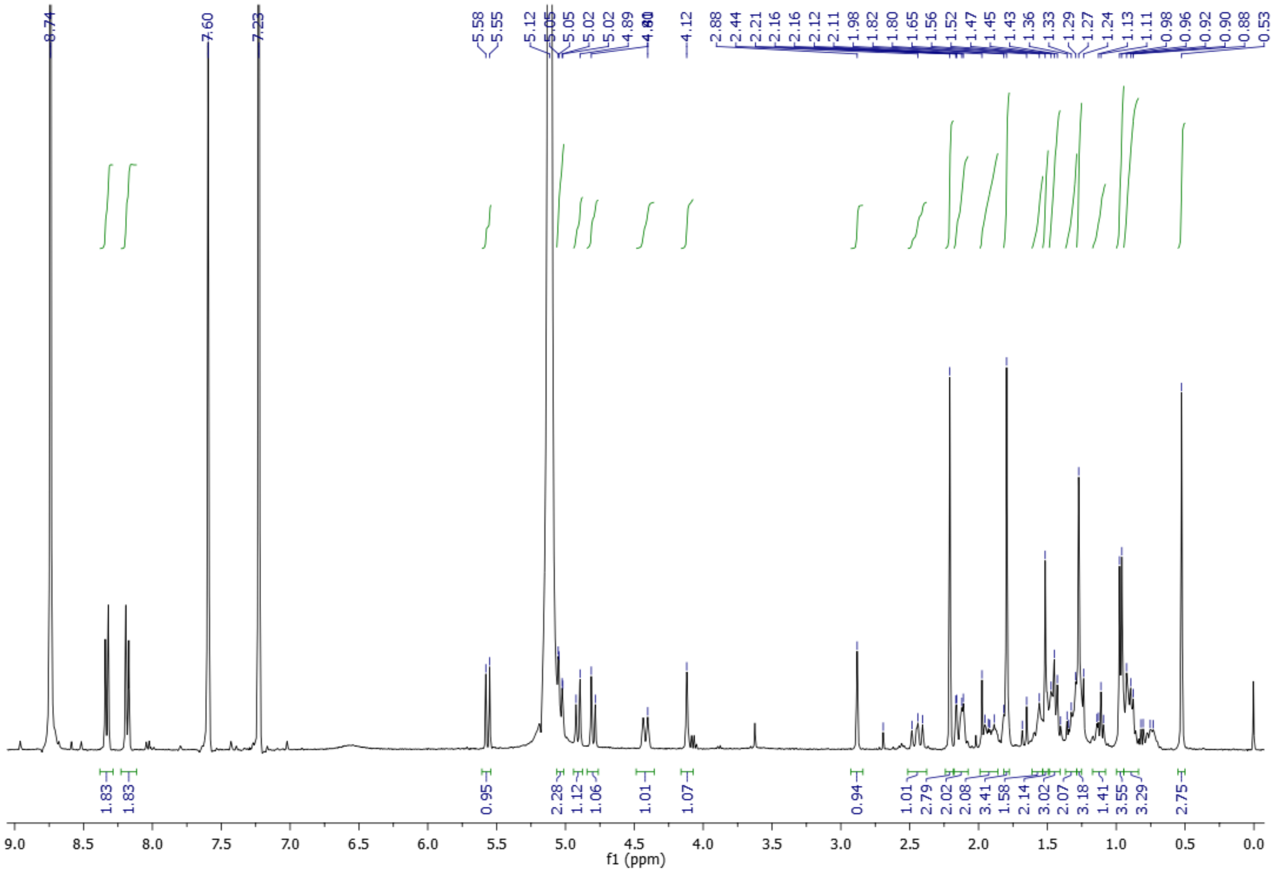

^{13}C NMR of W-2a

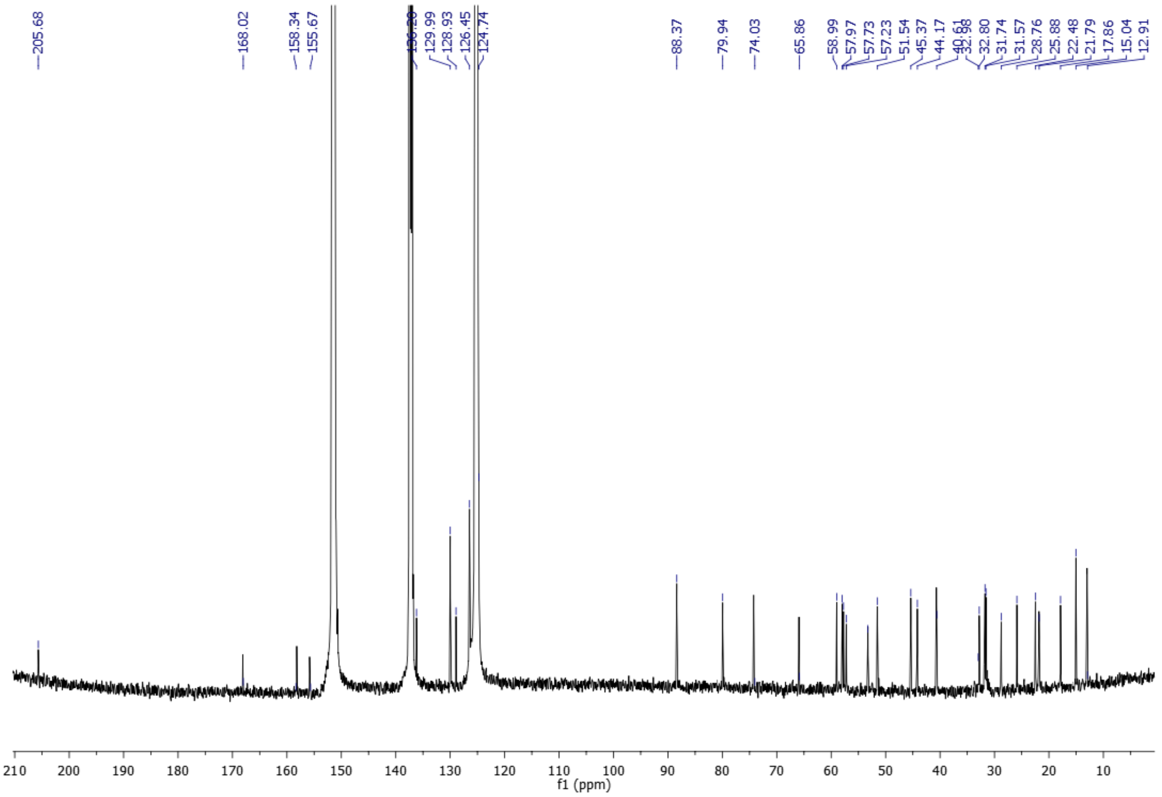

^{1}H NMR of W-2b

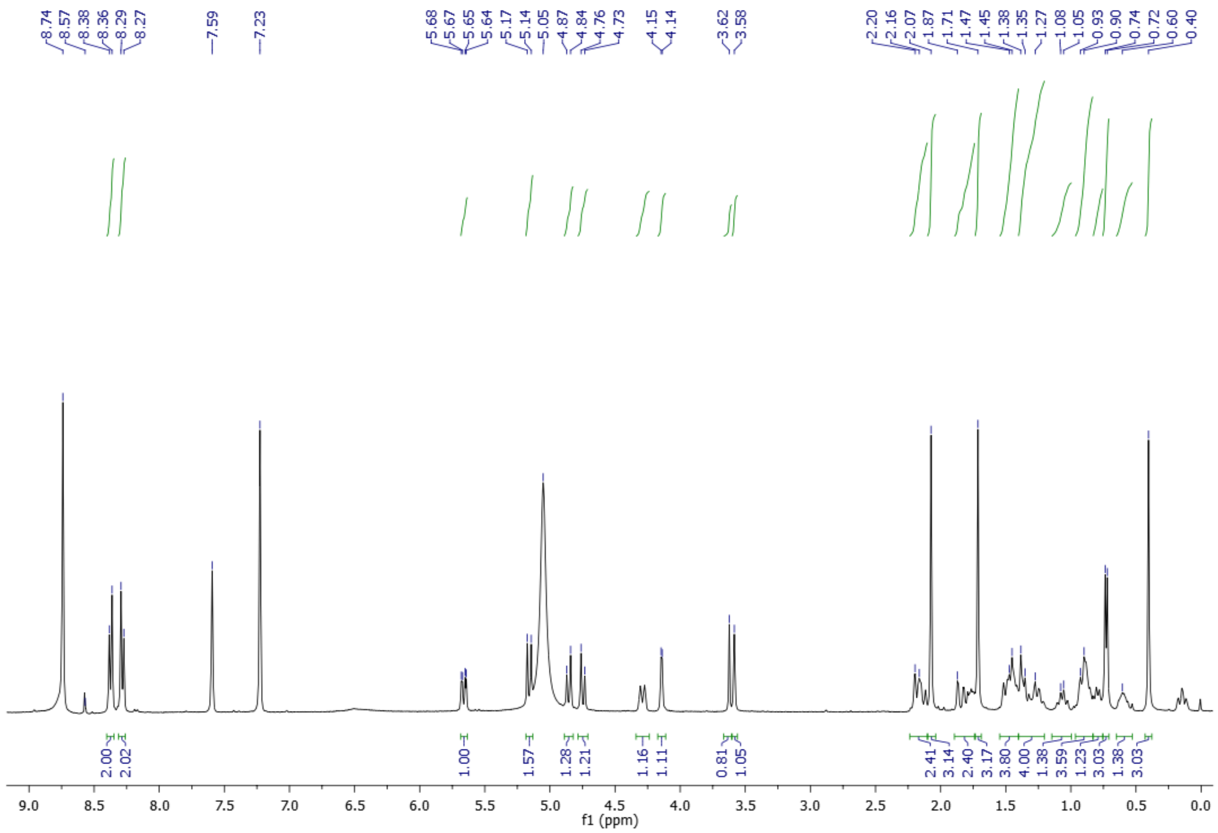

^{13}C NMR of W-2b

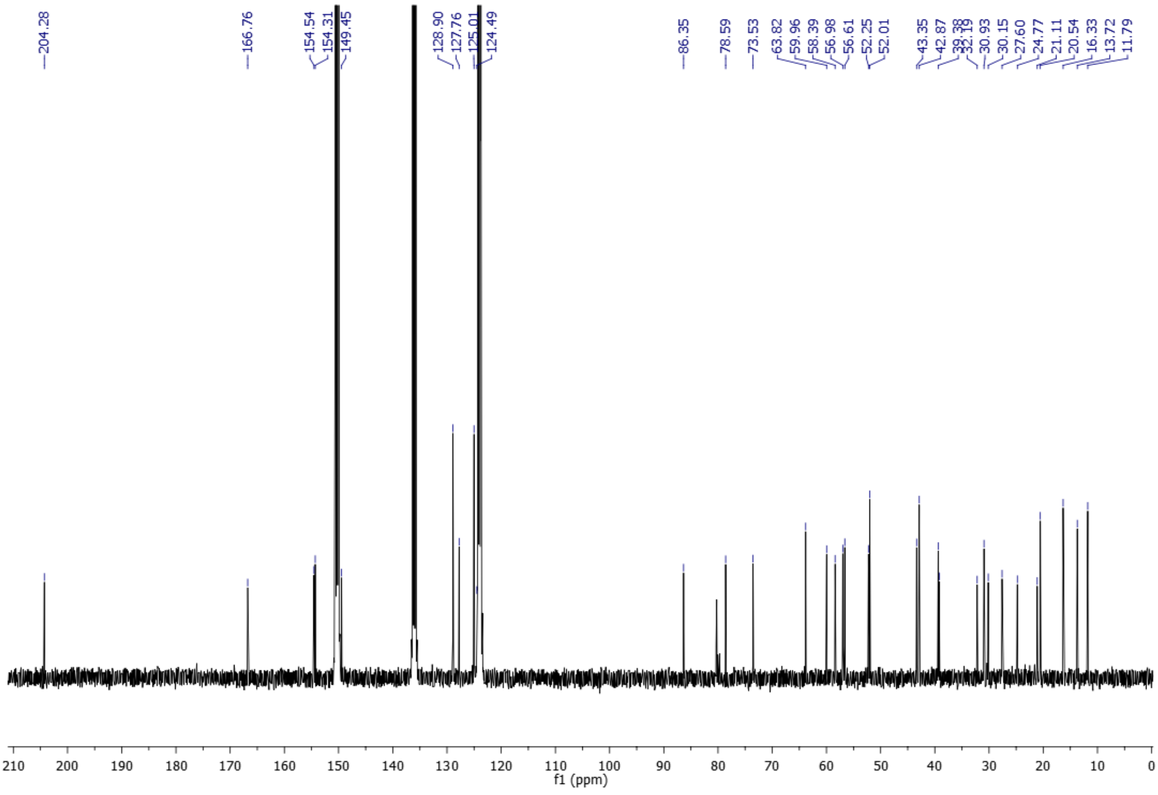

^{1}H NMR of W-3a

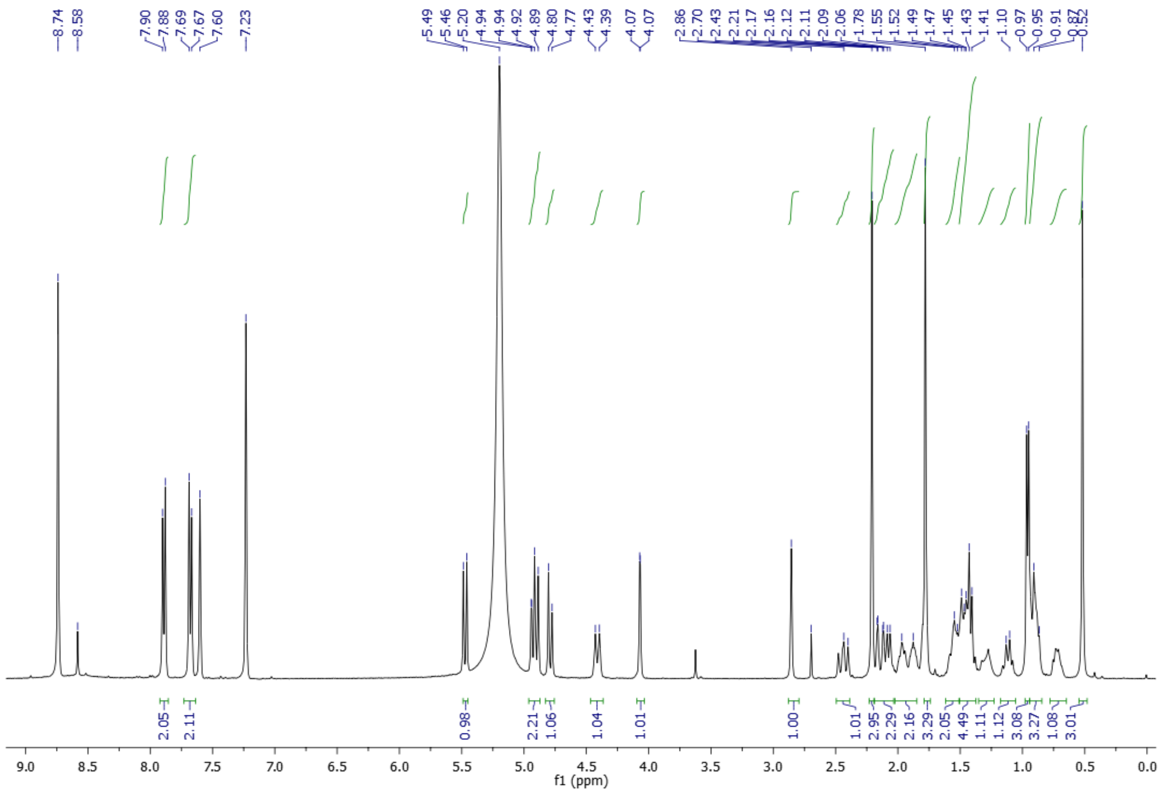

^{13}C NMR of W-3a

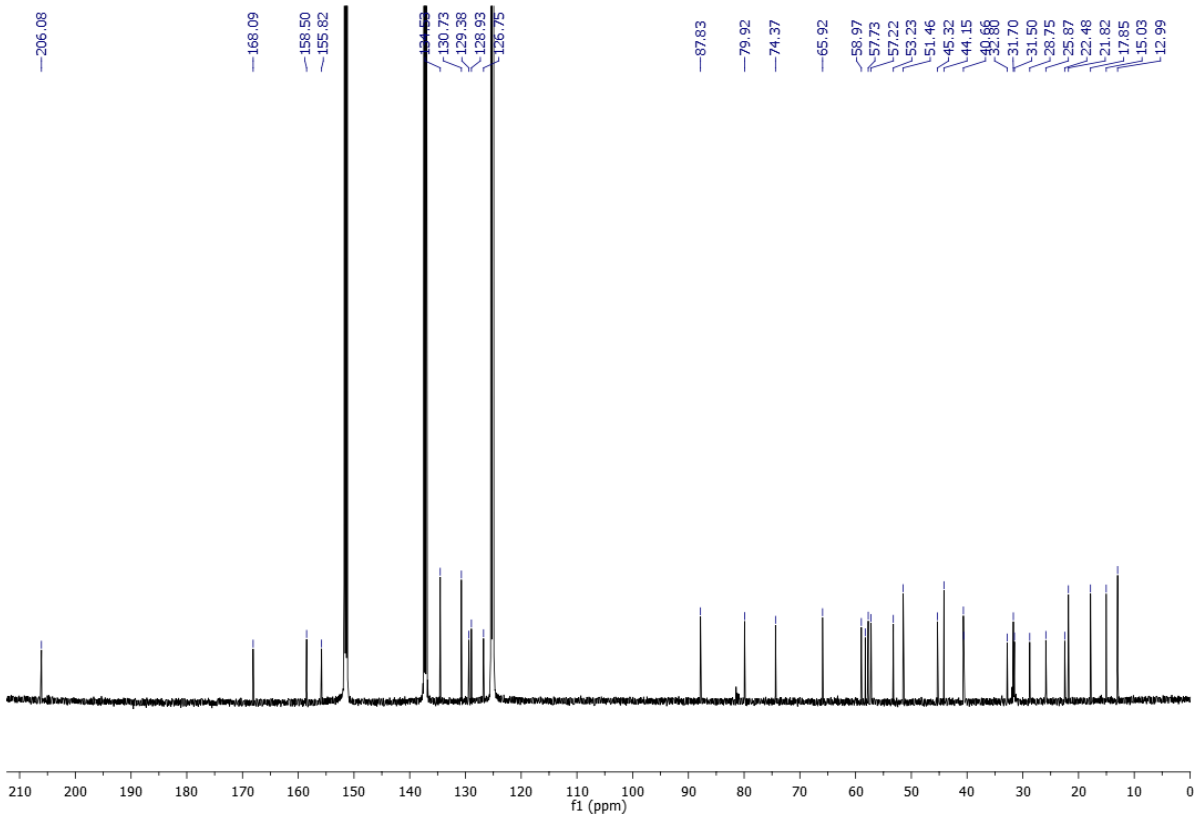

^{1}H NMR of W-3b

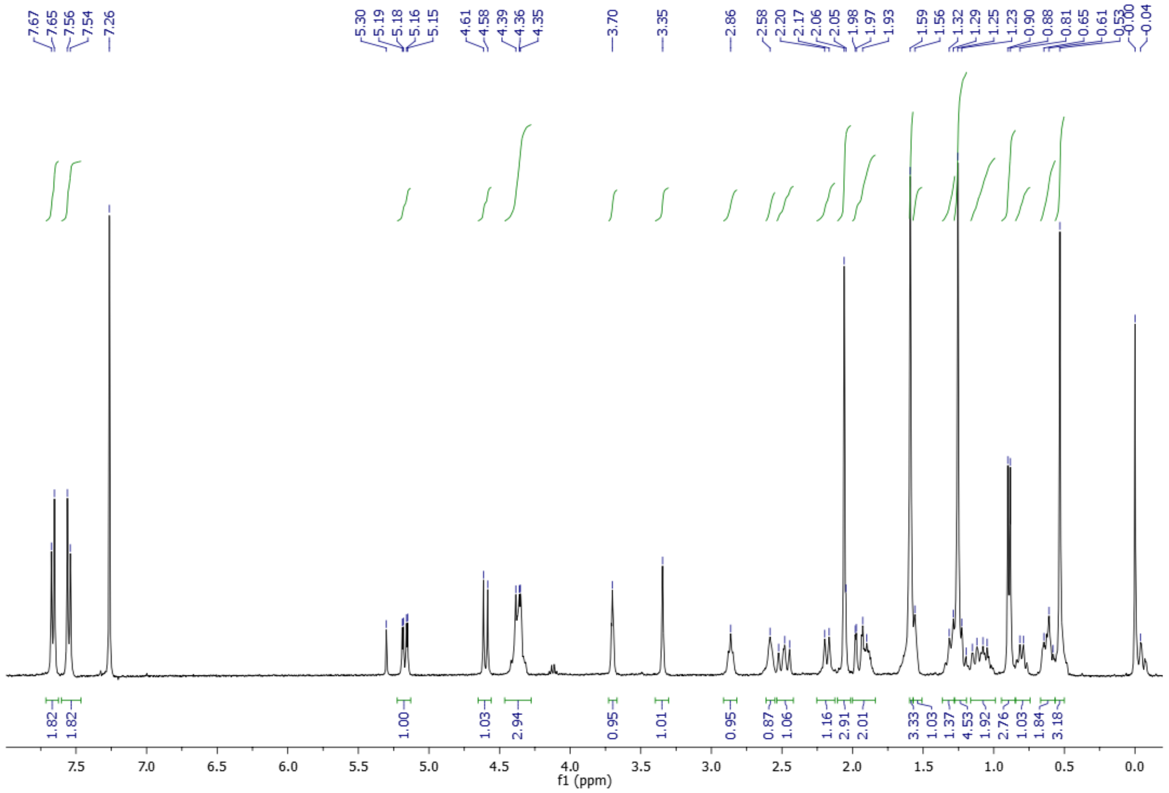

^{13}C NMR of W-3b

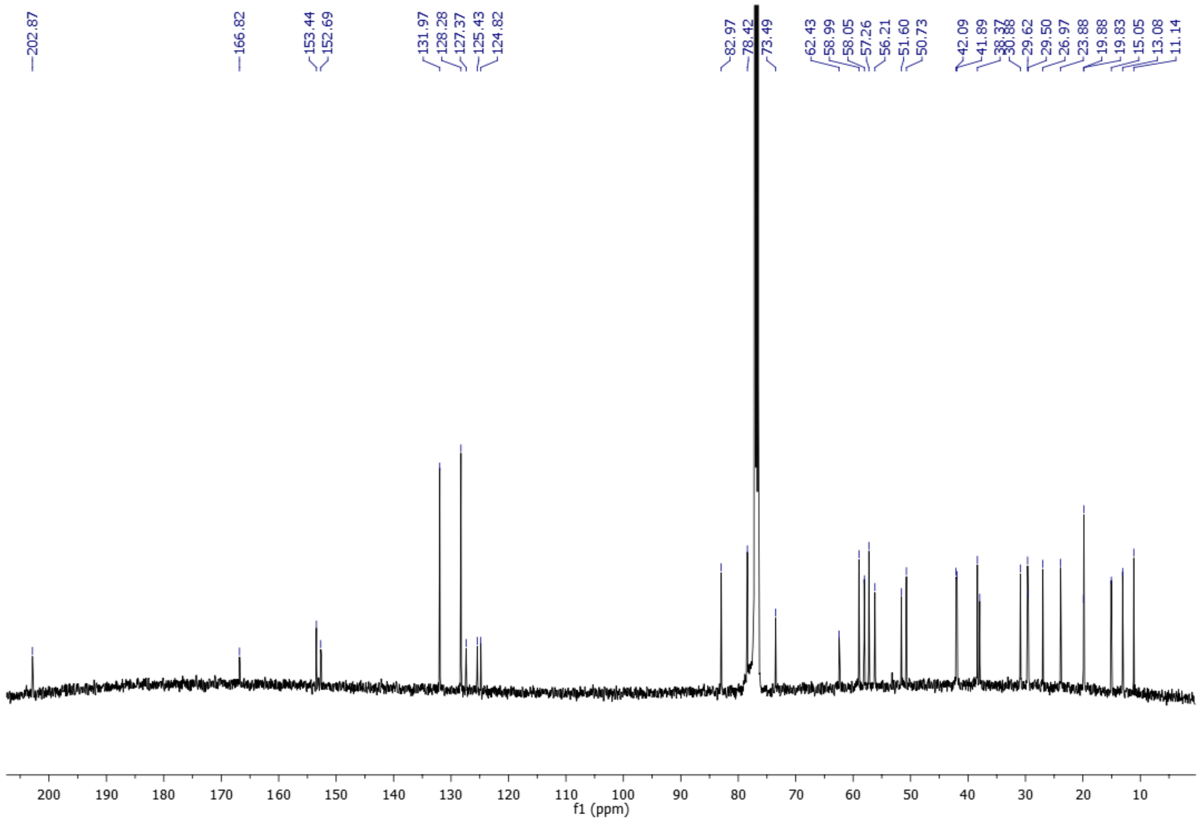

^{1}H NMR of W-4a

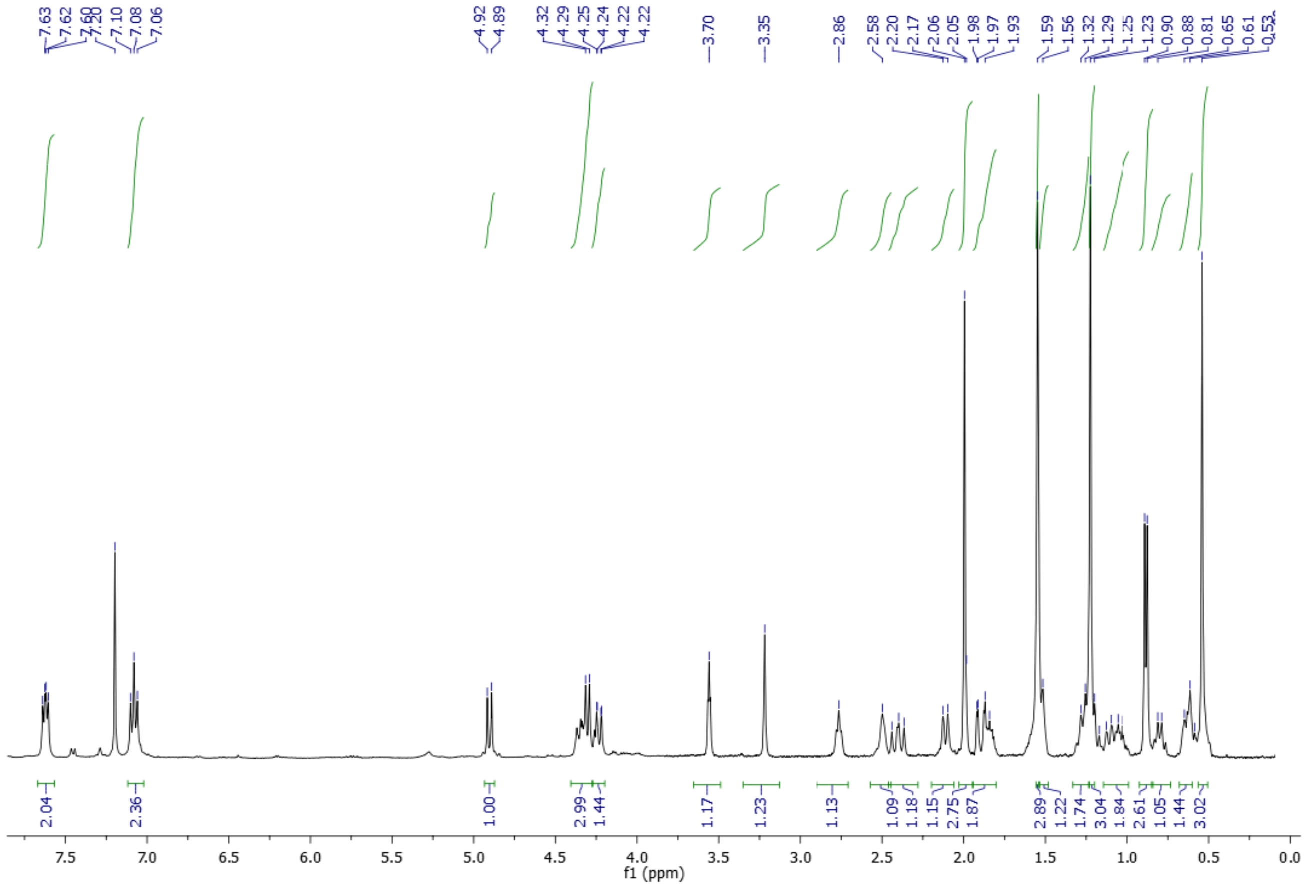

^{13}C NMR of W-4a

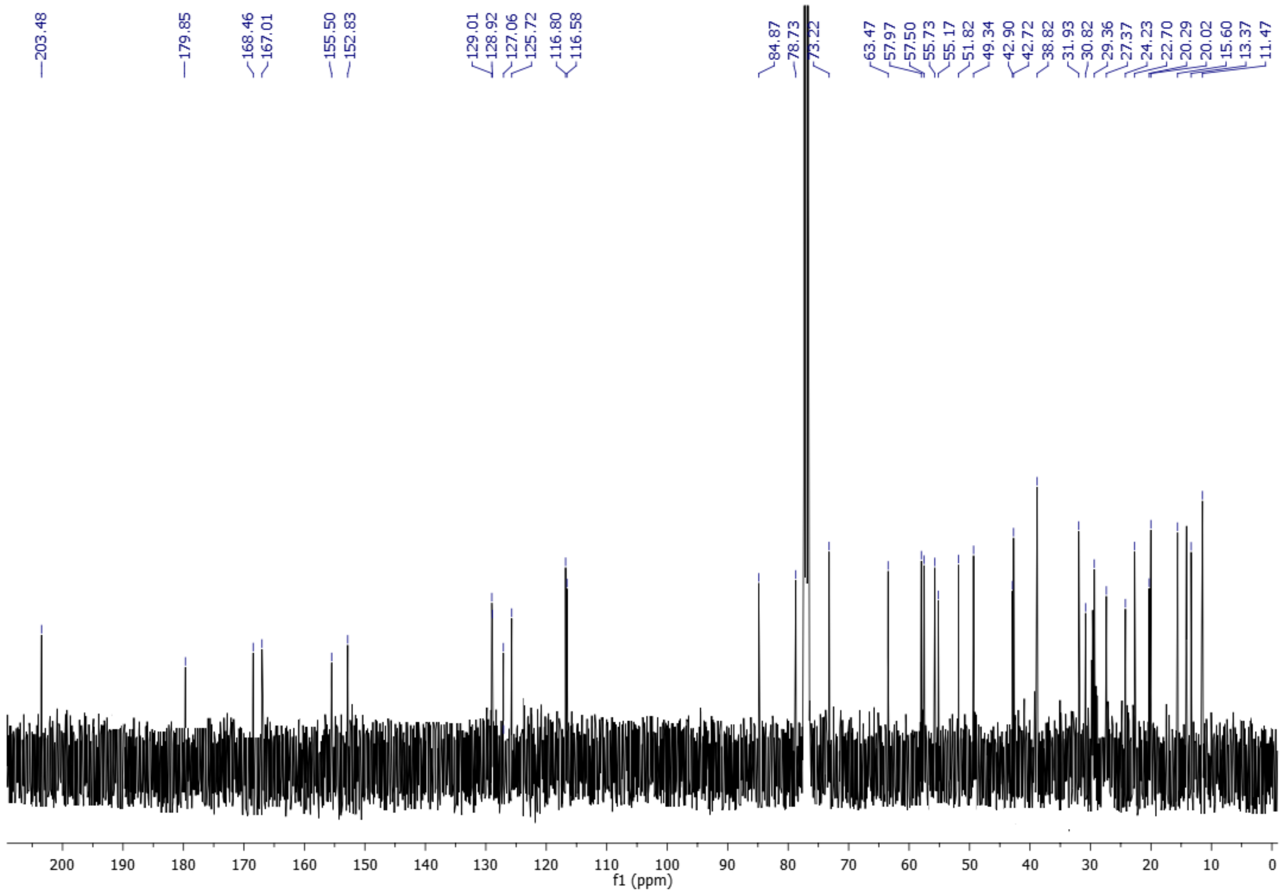

^{1}H NMR of W-4b

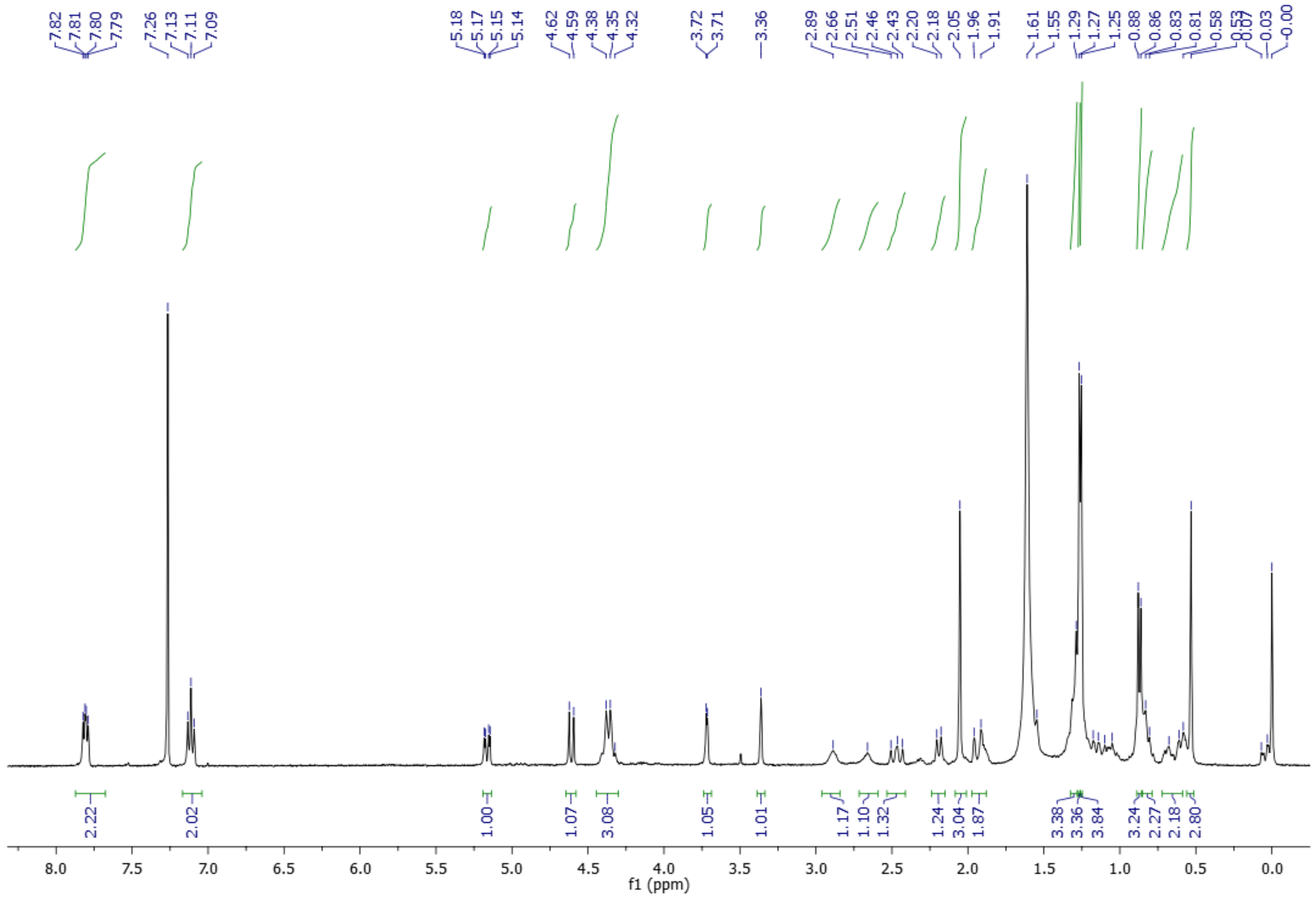

7.82
7.81
7.80
7.79
7.26
7.13
7.11
7.09
5.18
5.17
5.15
5.14
4.62
4.59
4.38
4.35
4.32
3.72
3.71
3.36
2.89
2.66
2.51
2.46
2.43
2.20
2.18
2.05
1.96
1.91
1.61
1.55
1.29
1.27
1.25
0.88
0.86
0.83
0.81
0.58
0.53
0.07
0.03
0.00
2.22
2.02
1.00
1.07
3.08
1.05
1.01
1.17
1.10
1.32
1.24
3.04
1.87
3.38
3.36
3.84
3.24
2.27
2.18
2.80
f1 (ppm)

^{13}C NMR of W-4b

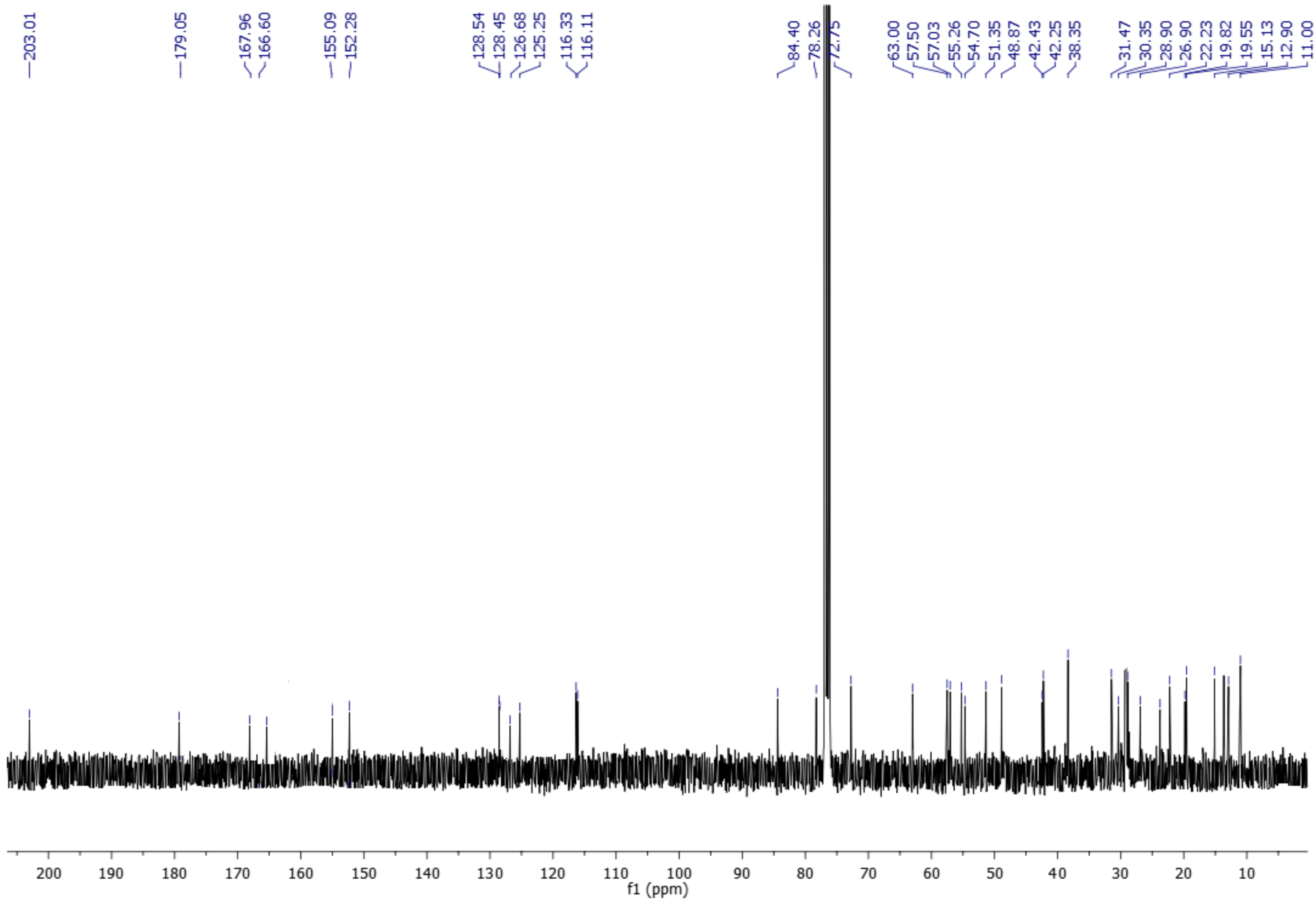

^{1}H NMR of W-5a

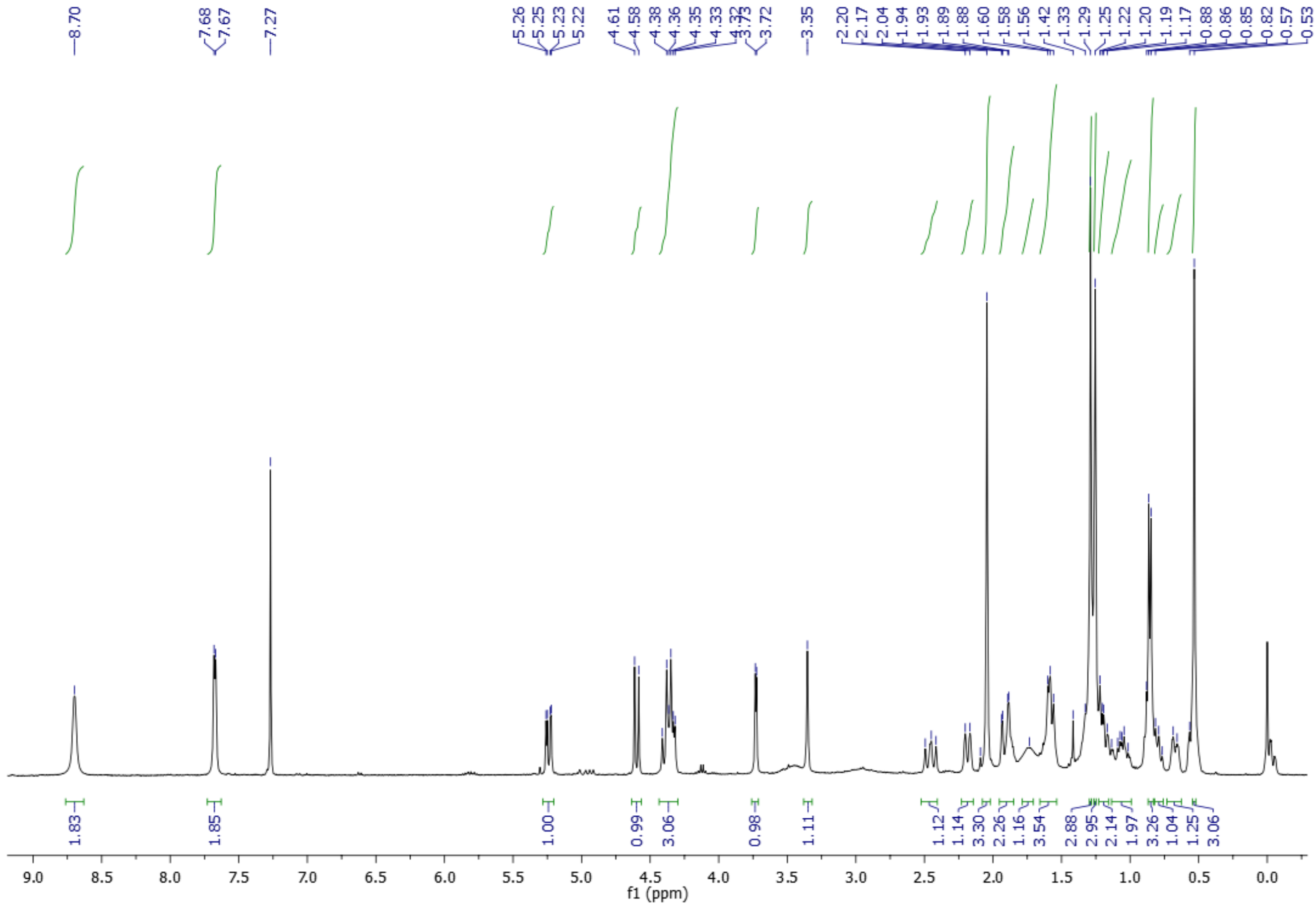

^{13}C NMR of W-5a

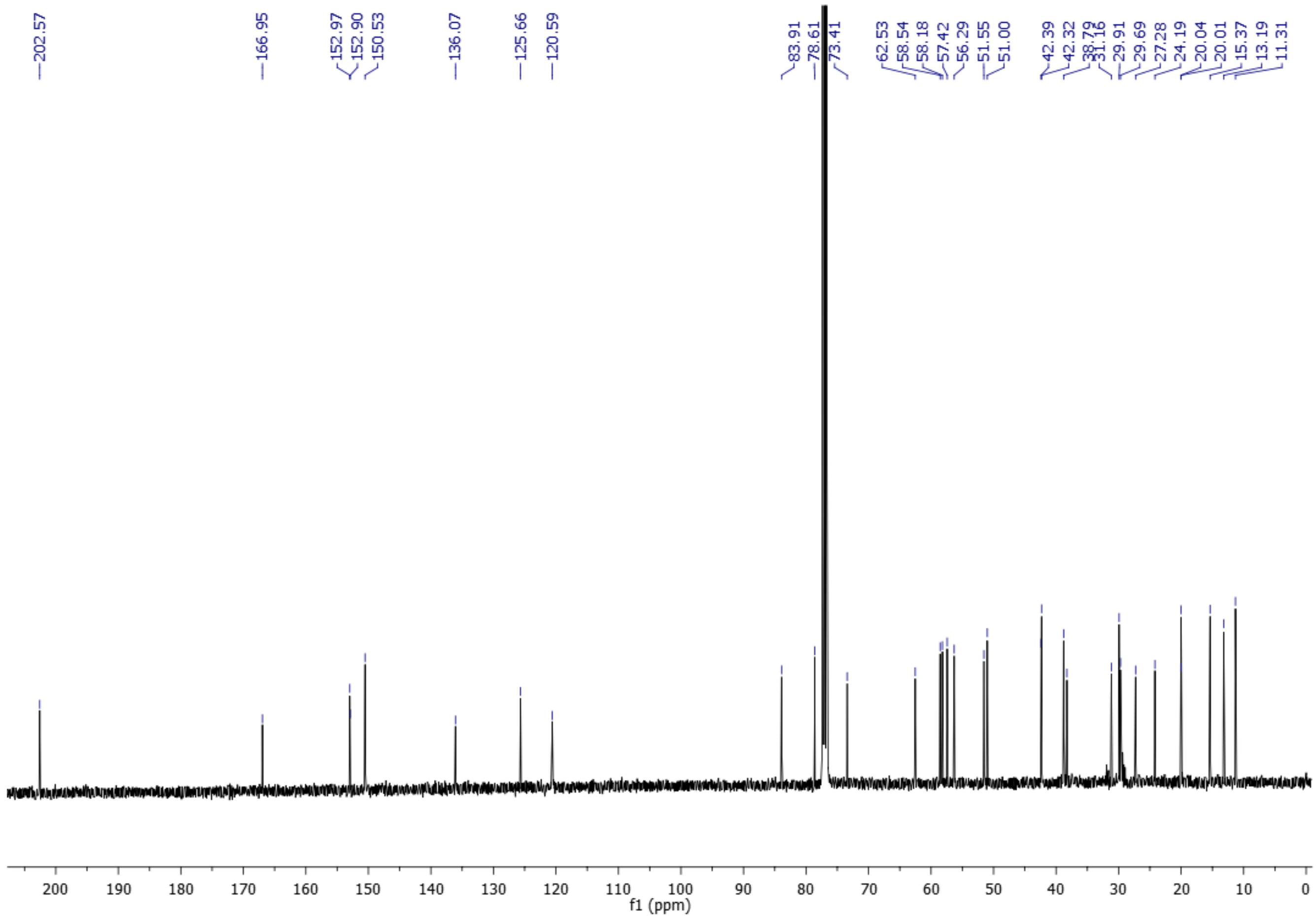

^{1}H NMR of W-5b

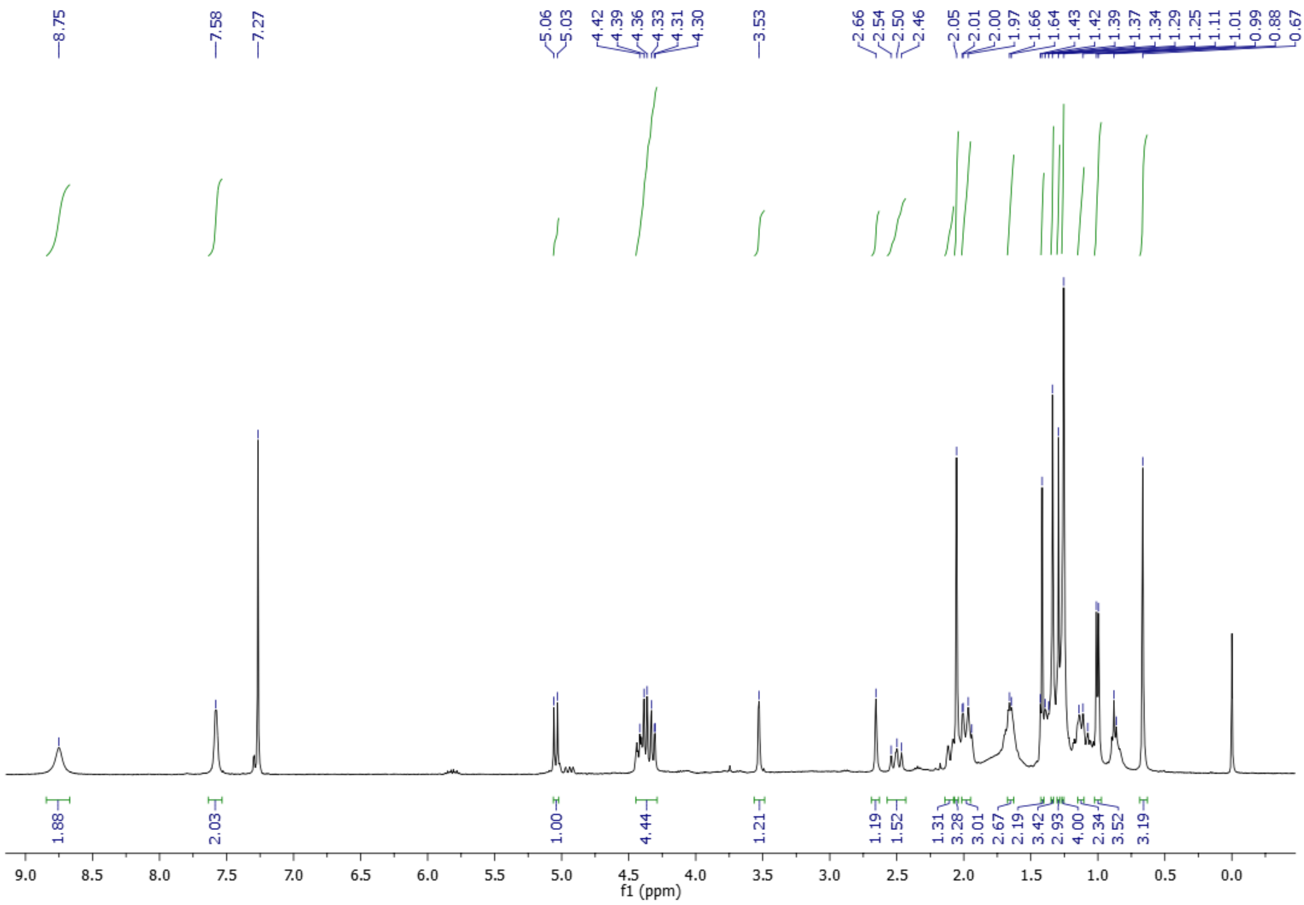

^{1}H NMR of W-5b

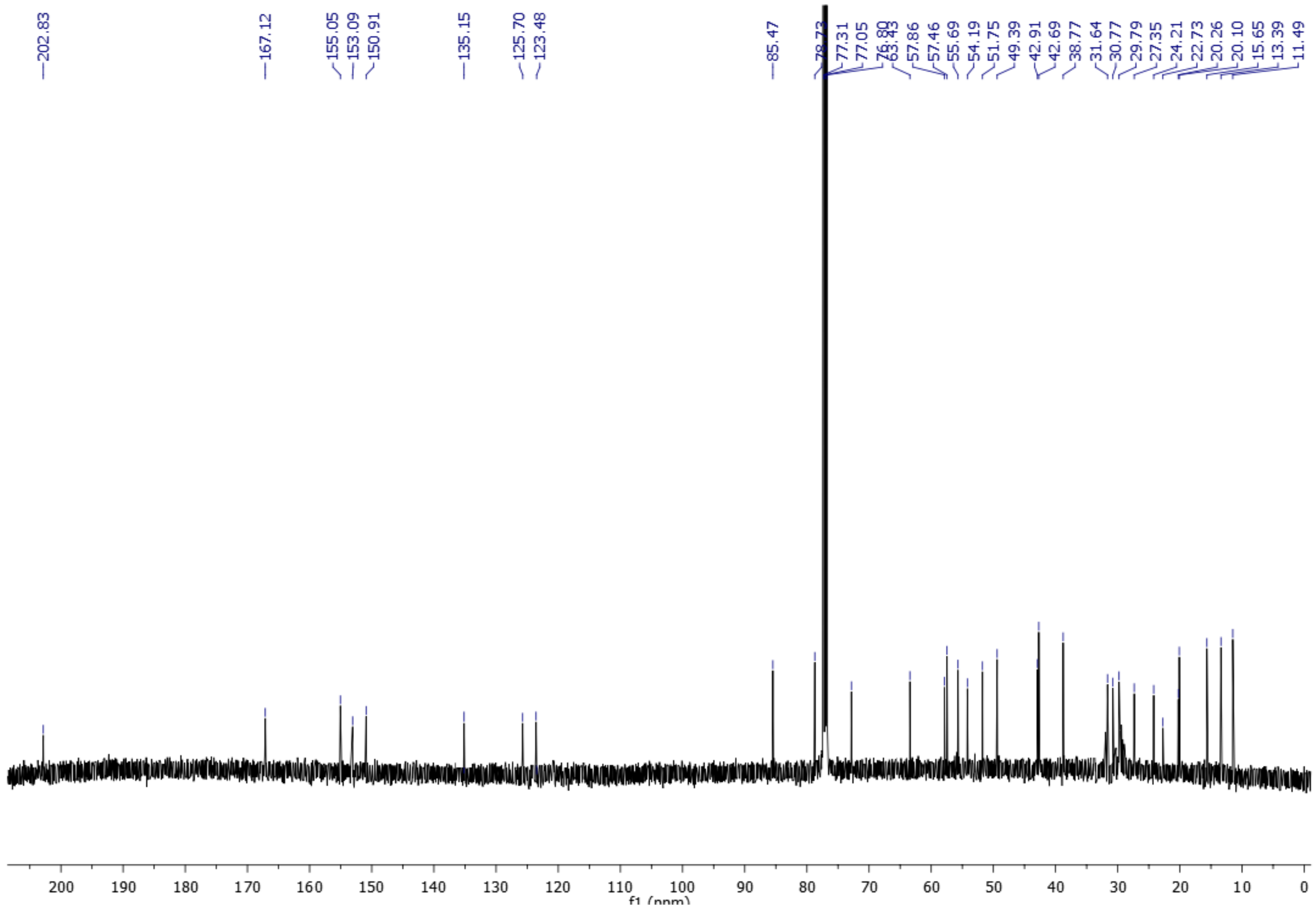

^{1}H NMR of W-6a

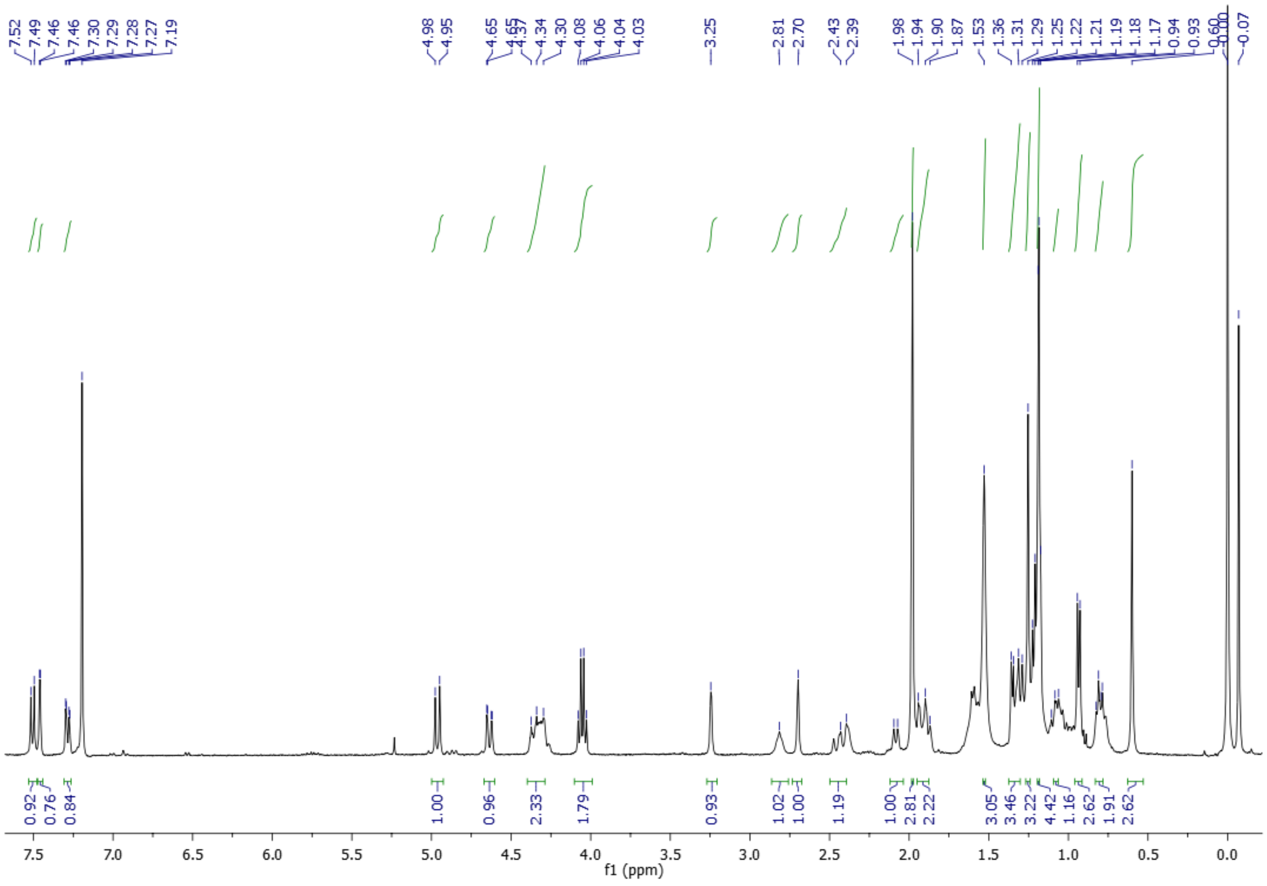

7.52
7.49
7.46
7.46
7.30
7.29
7.28
7.27
7.19
4.98
4.95
4.65
4.62
4.37
4.34
4.30
4.08
4.06
4.04
4.03
3.25
2.81
2.70
2.43
2.39
1.98
1.94
1.90
1.87
1.53
1.36
1.31
1.29
1.25
1.22
1.21
1.19
1.18
1.17
0.94
0.93
0.60
0.00
-0.07
0.92
0.76
0.84
1.00
0.96
2.33
1.79
0.93
1.02
1.00
1.19
1.00
2.81
2.22
3.05
3.46
3.22
4.42
1.16
2.62
1.91
2.62
f1 (ppm)

^{13}C NMR of W-6a

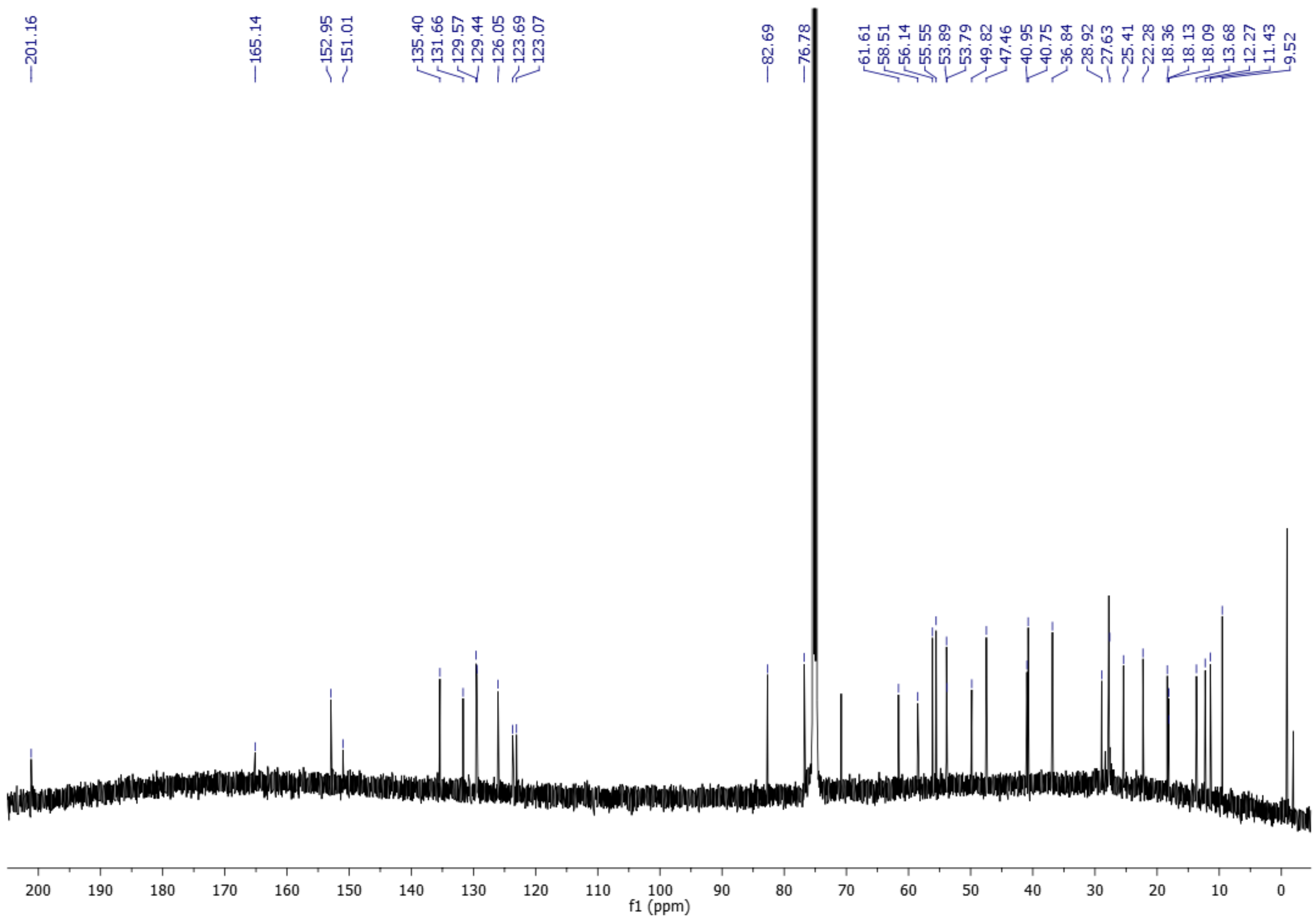

^{1}H NMR of W-6b

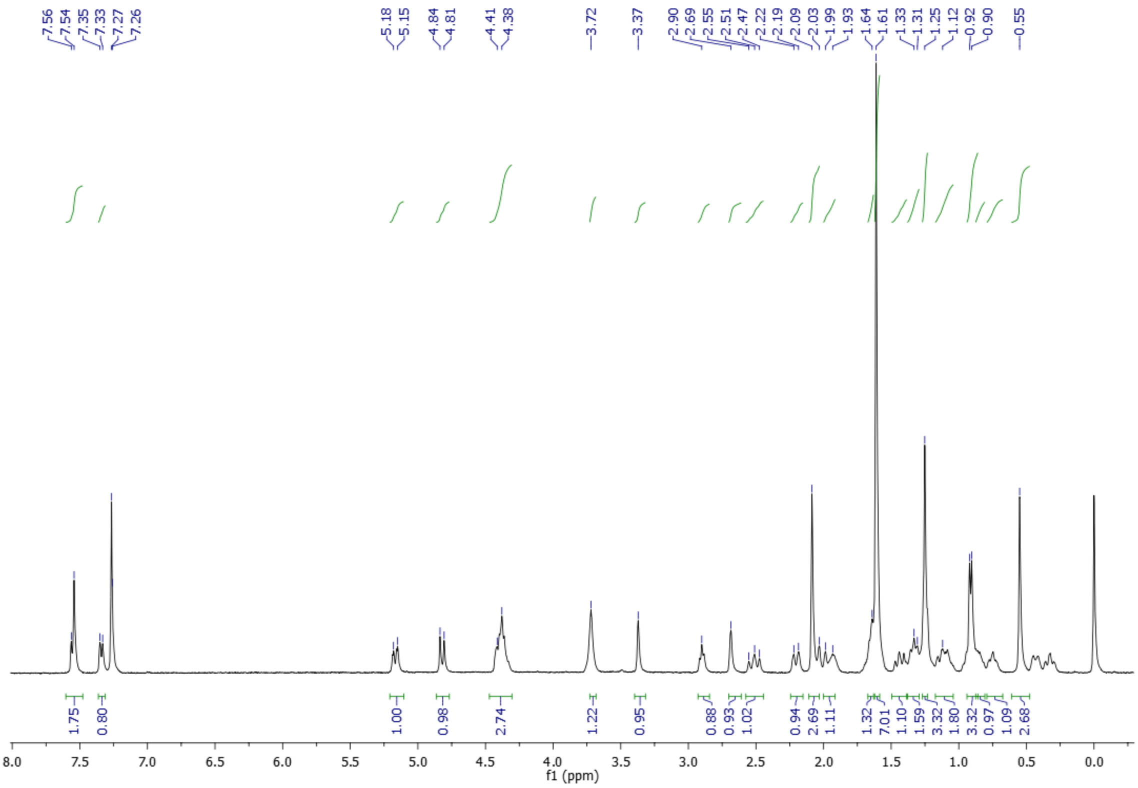

^{13}C NMR of W-6b

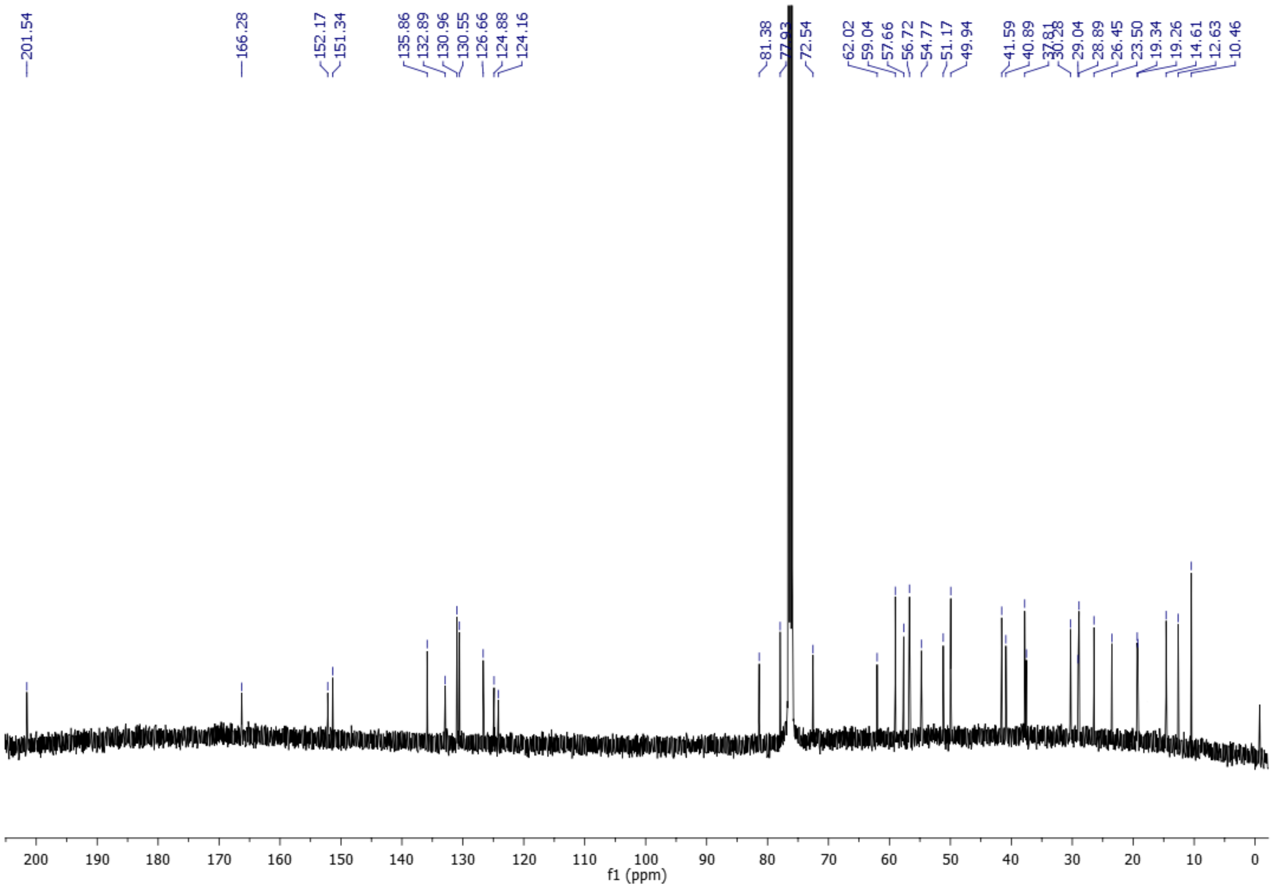

^{1}H NMR of W-7a

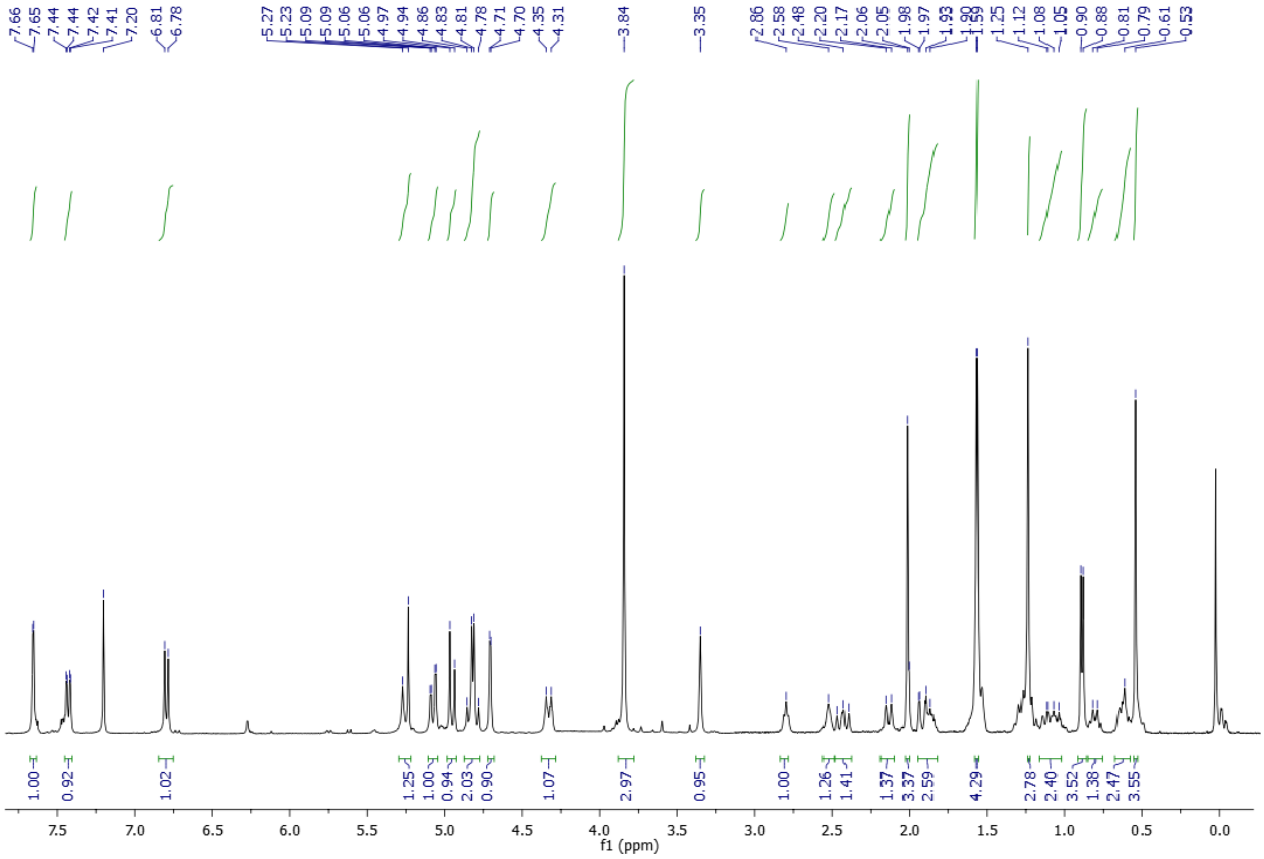

^{13}C NMR of W-7a

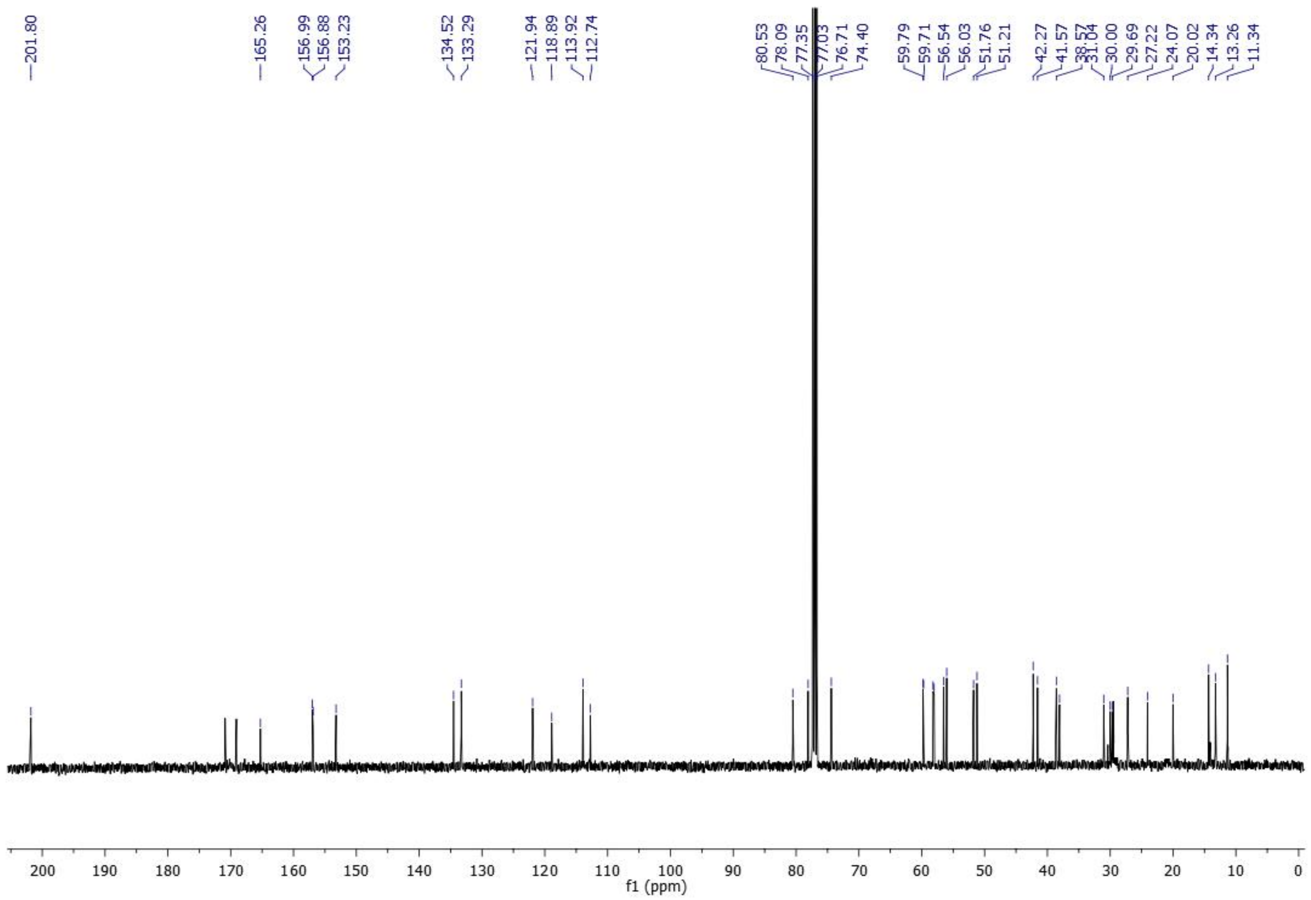

^{1}H NMR of W-8a

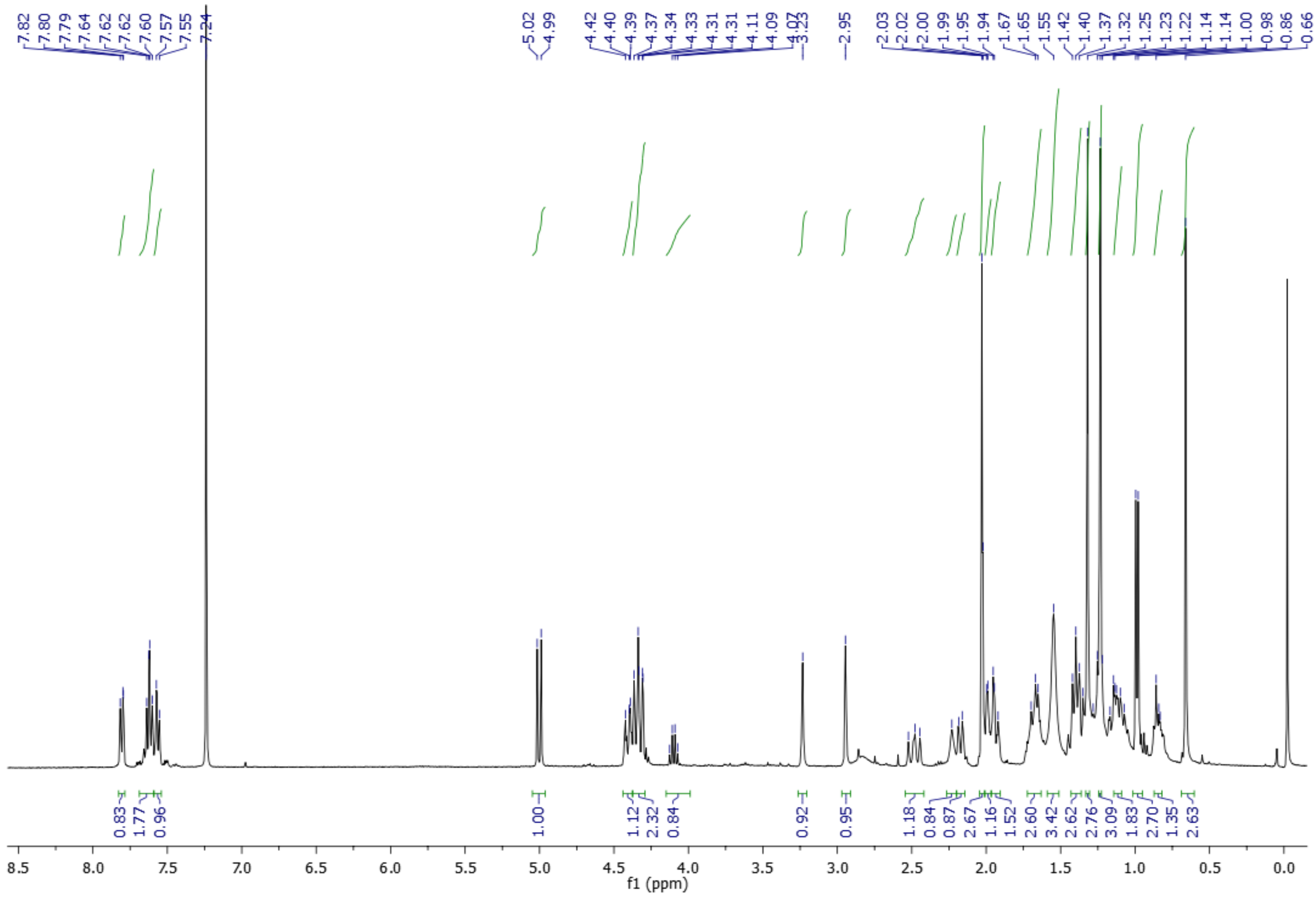

^{13}C NMR of W-8a

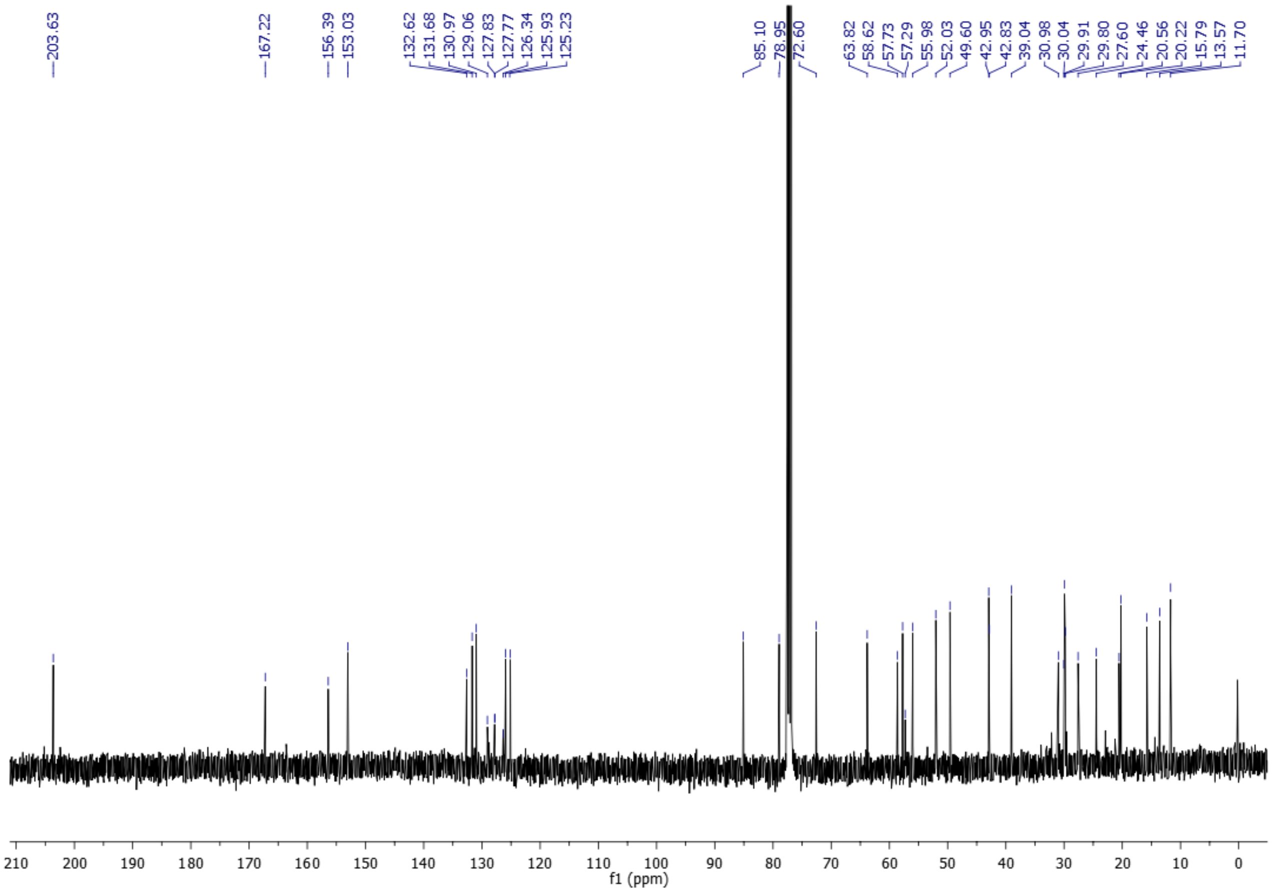

^{1}H NMR of W-9b

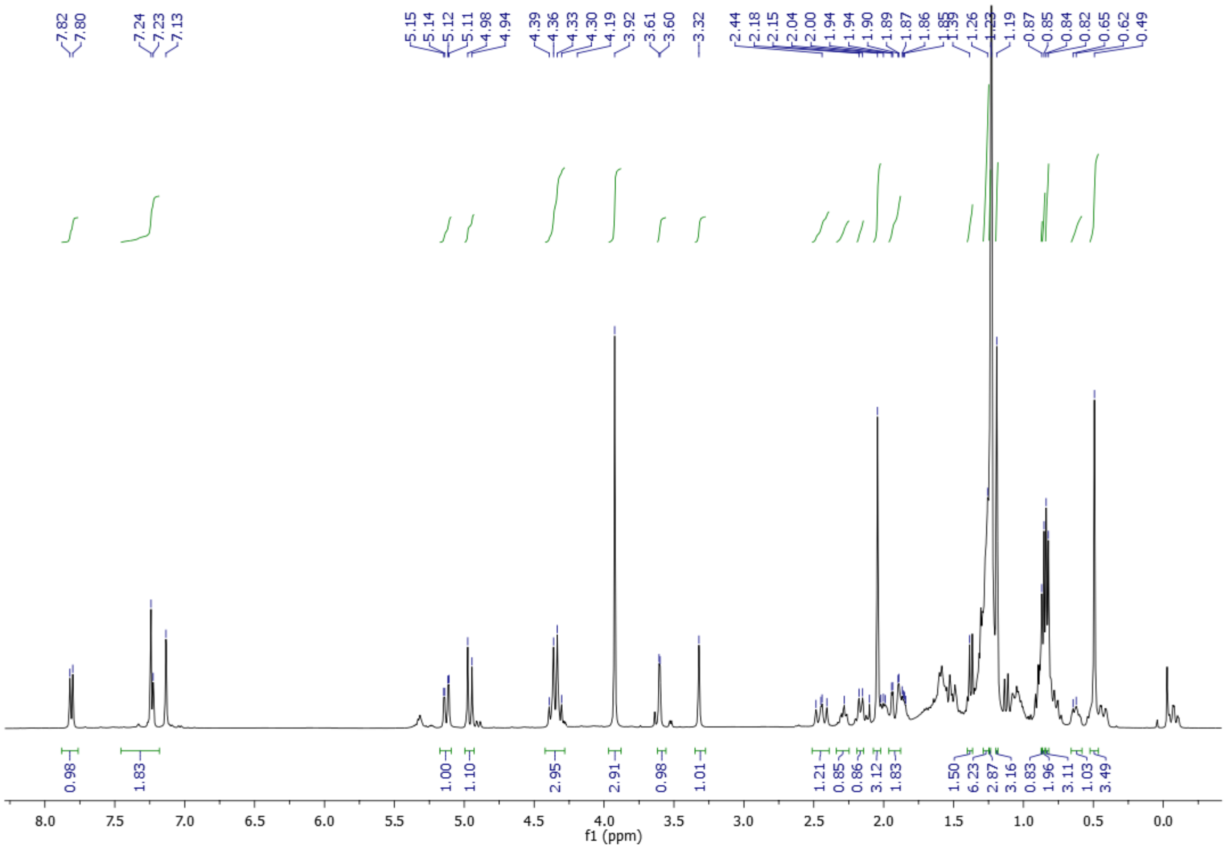
7.82
7.80
7.24
7.23
7.13
5.15
5.14
5.12
5.11
4.98
4.94
4.39
4.36
4.33
4.30
4.19
3.92
3.61
3.60
3.32
2.44
2.18
2.15
2.04
2.00
1.94
1.94
1.90
1.89
1.87
1.86
1.85
1.39
1.26
1.23
1.19
0.87
0.85
0.84
0.82
0.65
0.62
0.49
0.98
1.83
1.00
1.10
2.95
2.91
0.98
1.01
1.21
0.85
0.86
3.12
1.83
1.50
6.23
2.87
3.16
0.83
1.96
3.11
1.03
3.49
8.0
7.5
7.0
6.5
6.0
5.5
5.0
4.5
4.0
3.5
3.0
2.5
2.0
1.5
1.0
0.5
0.0
f1 (ppm)

^{13}C NMR of W-9b

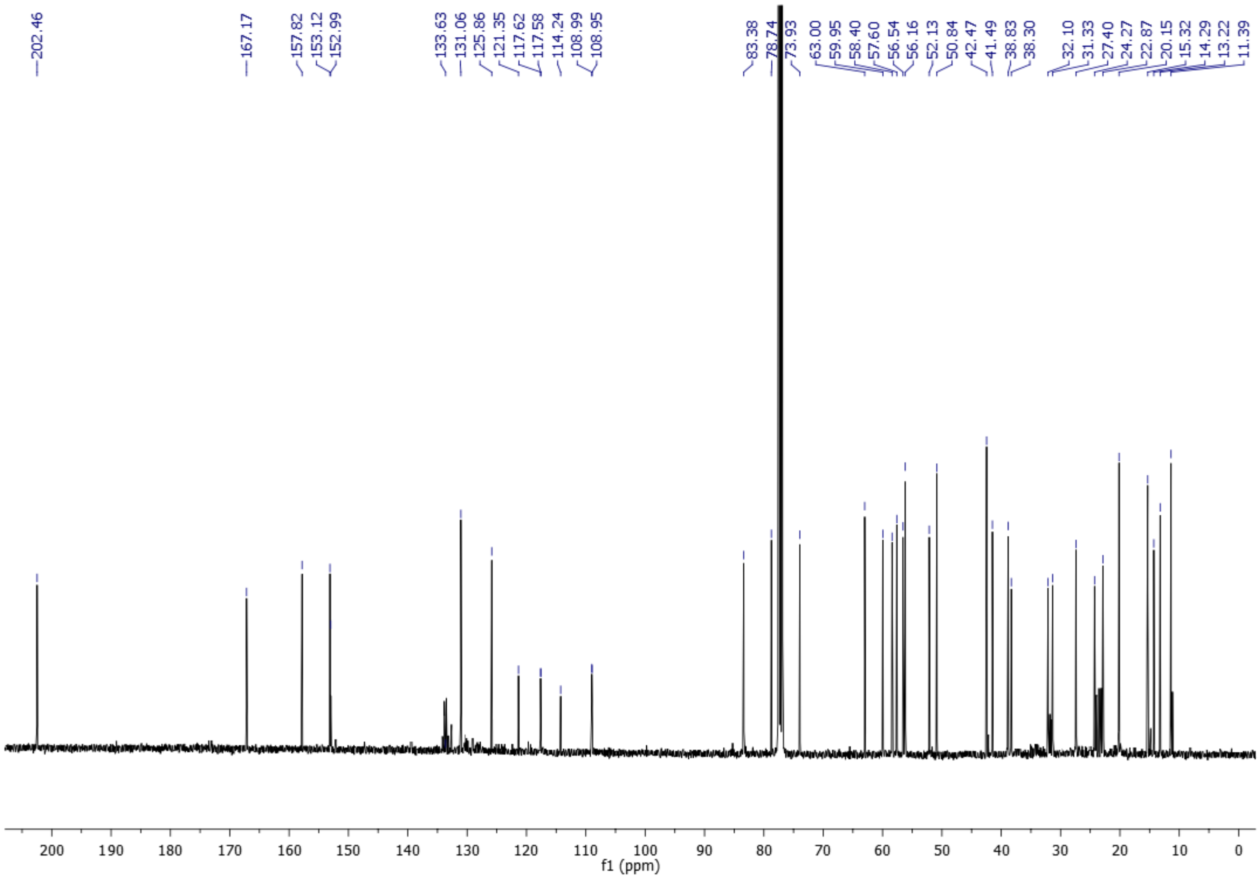

^{1}H NMR of W-10a

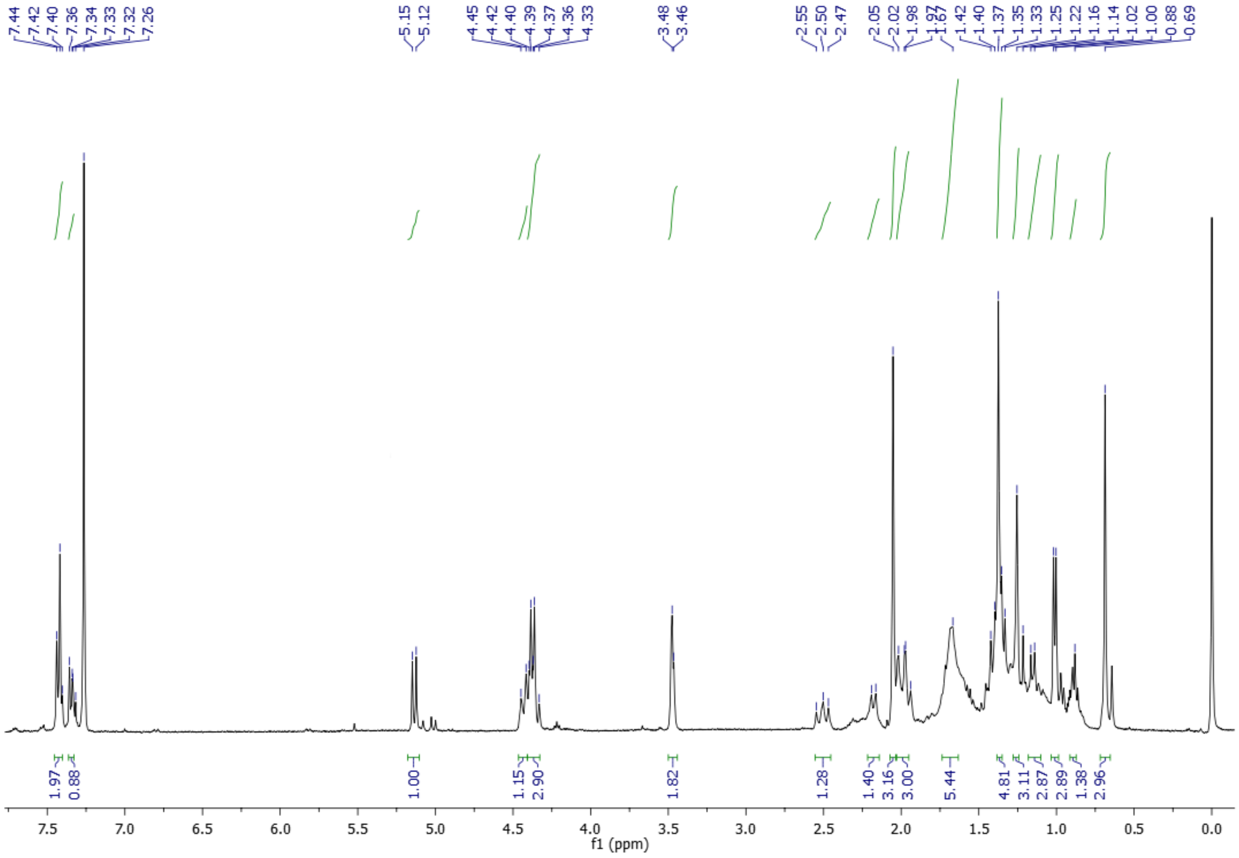

^{13}C NMR of W-10a

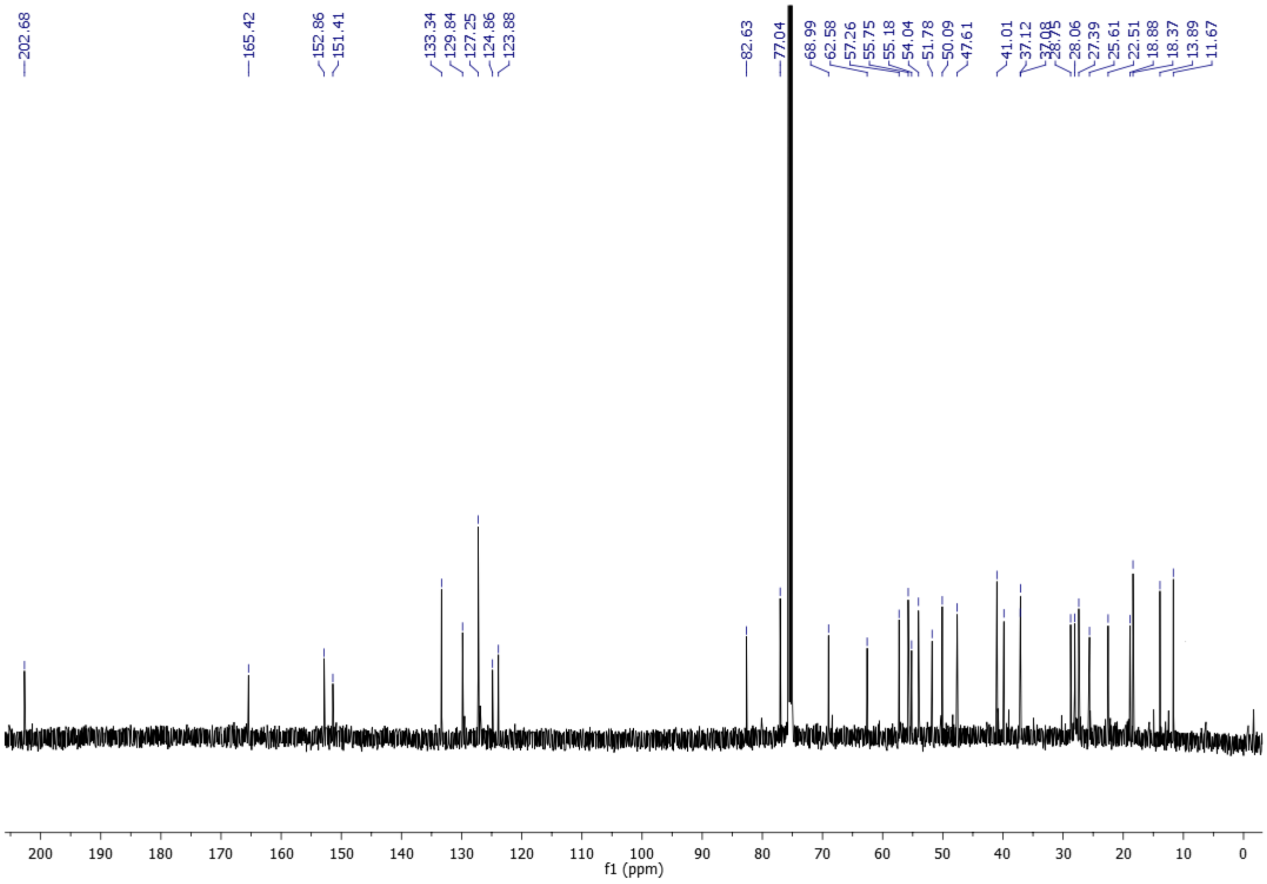

^{1}H NMR of W-11a

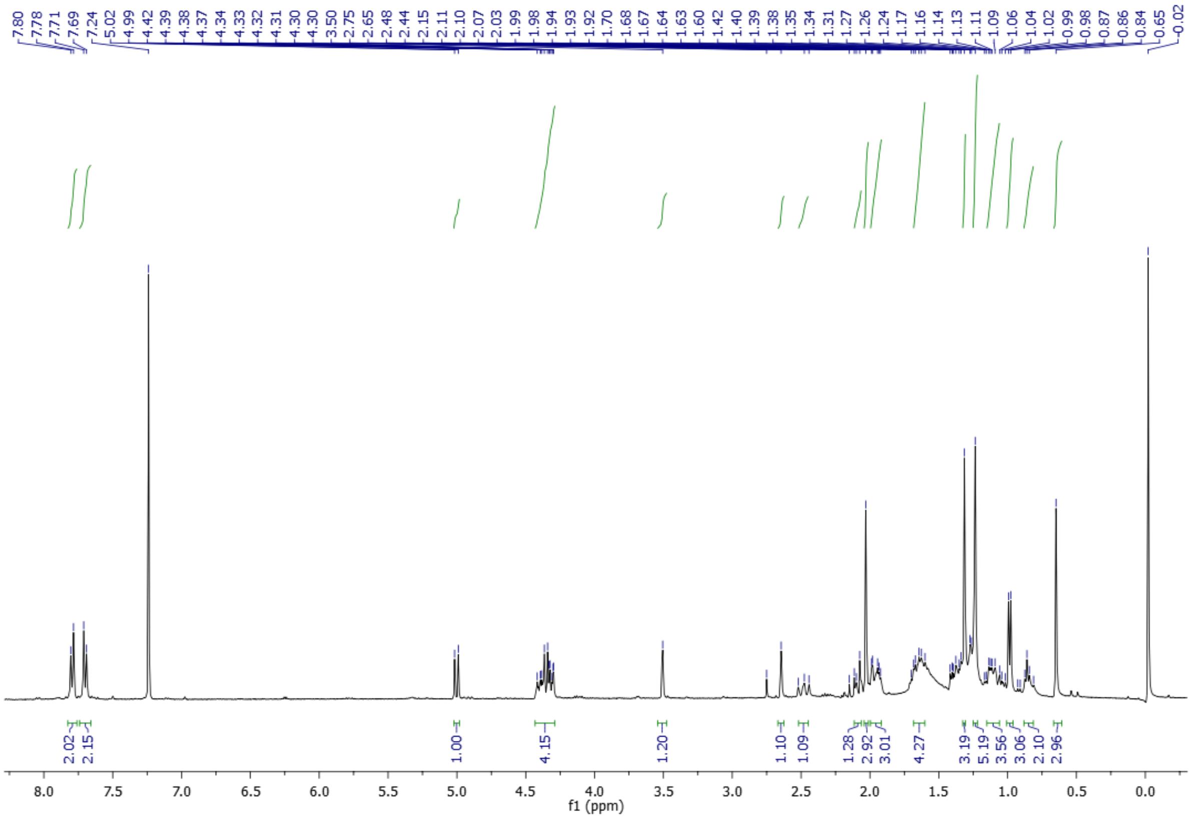

^{13}C NMR of W-11a

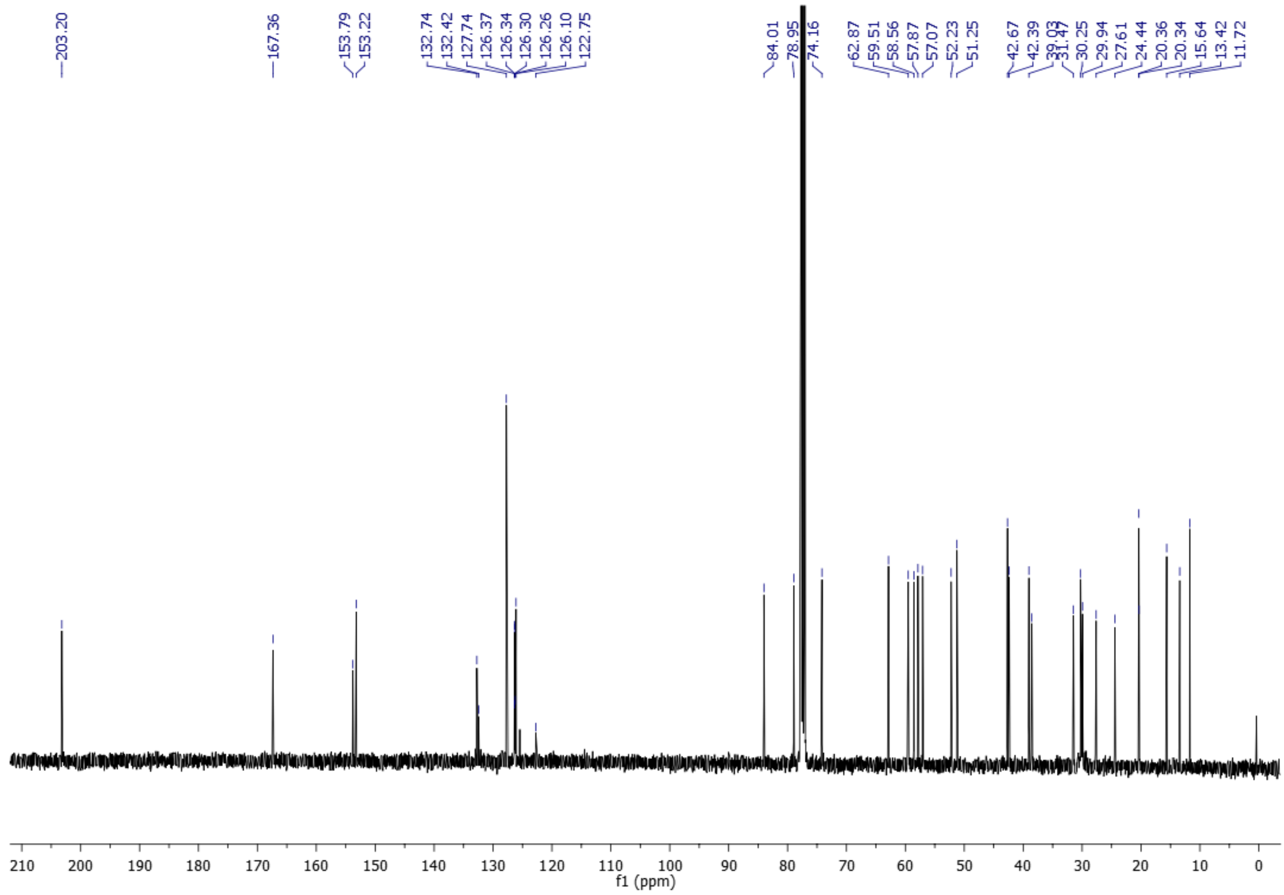

^{1}H NMR of W-11b

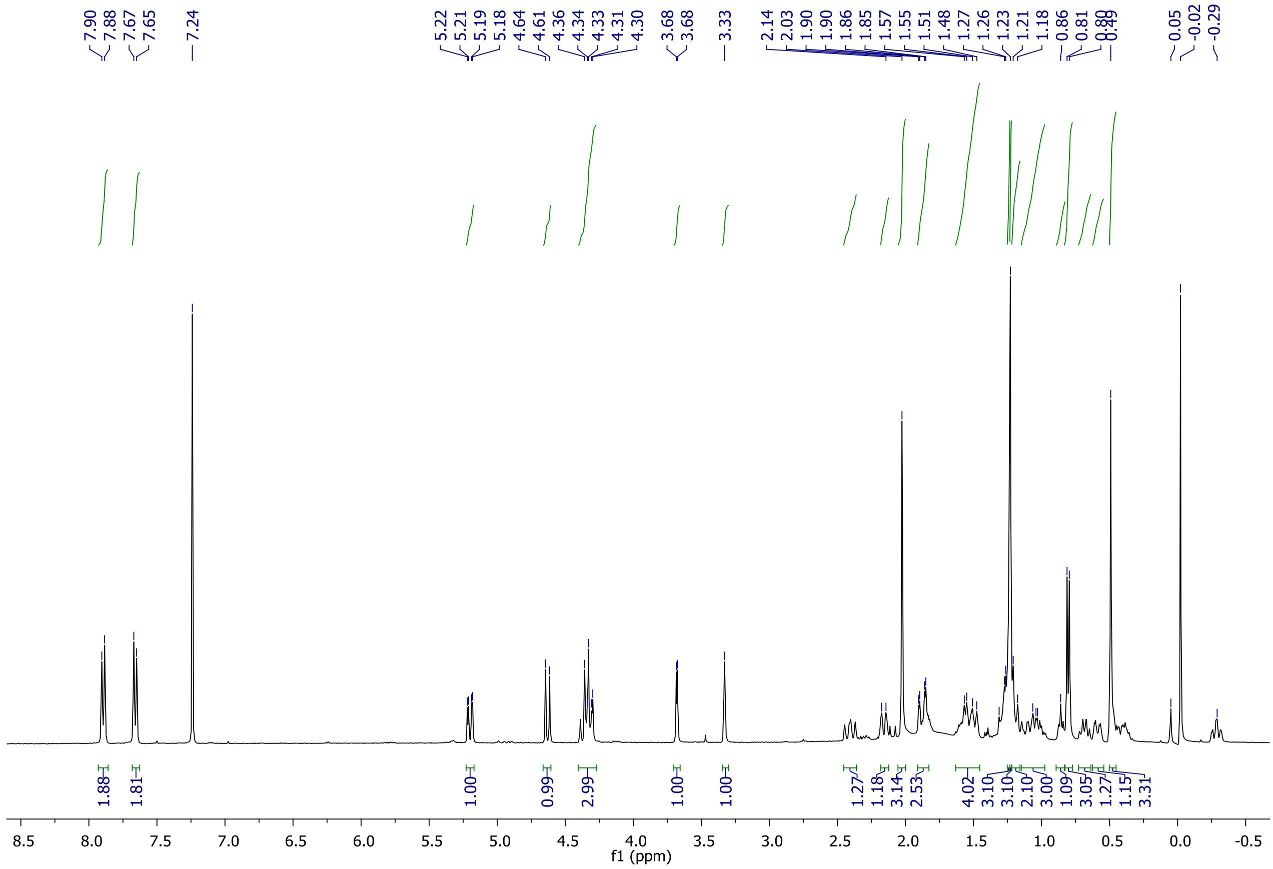

^{13}C NMR of W-11b

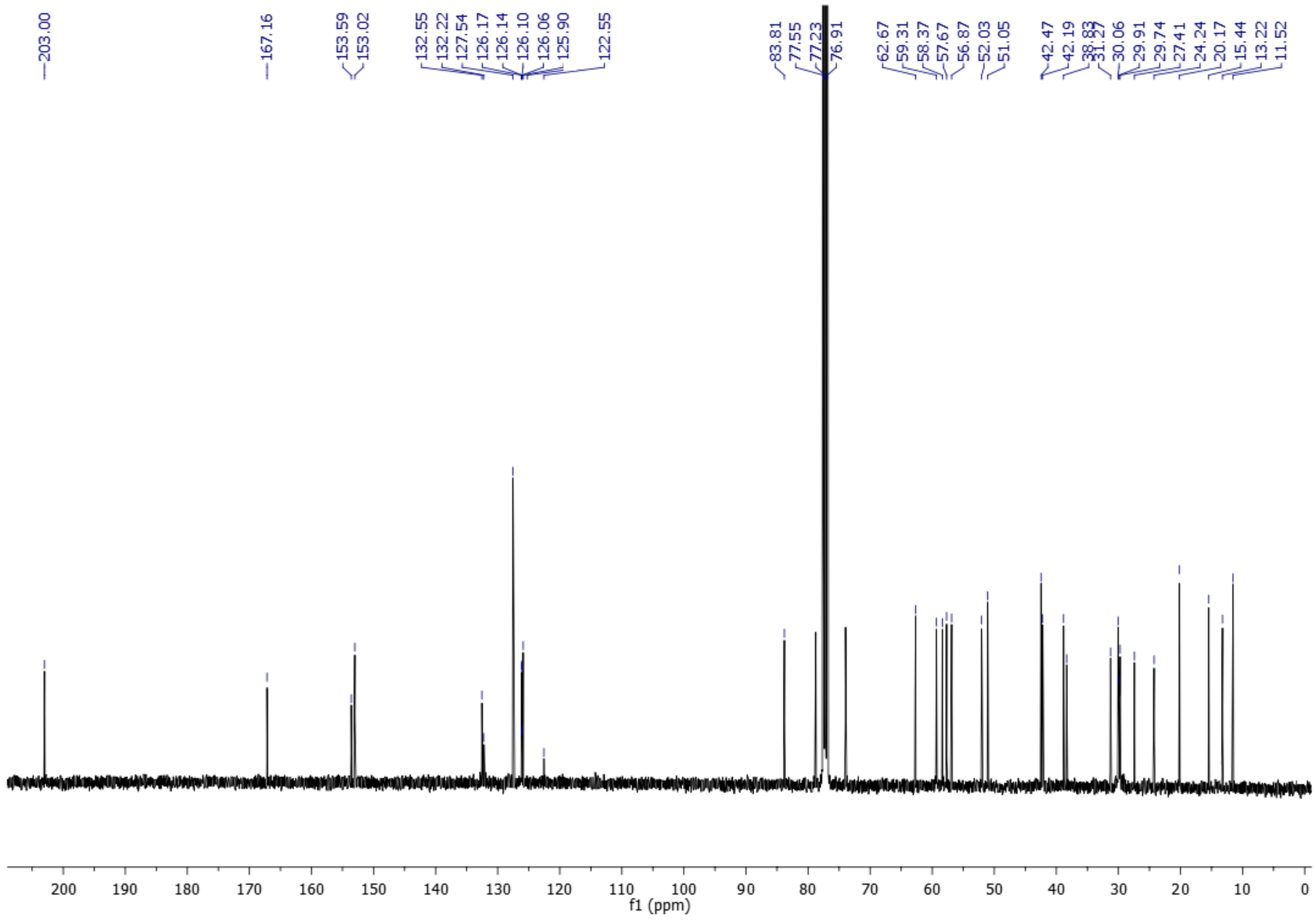

^{1}H NMR of W-12b

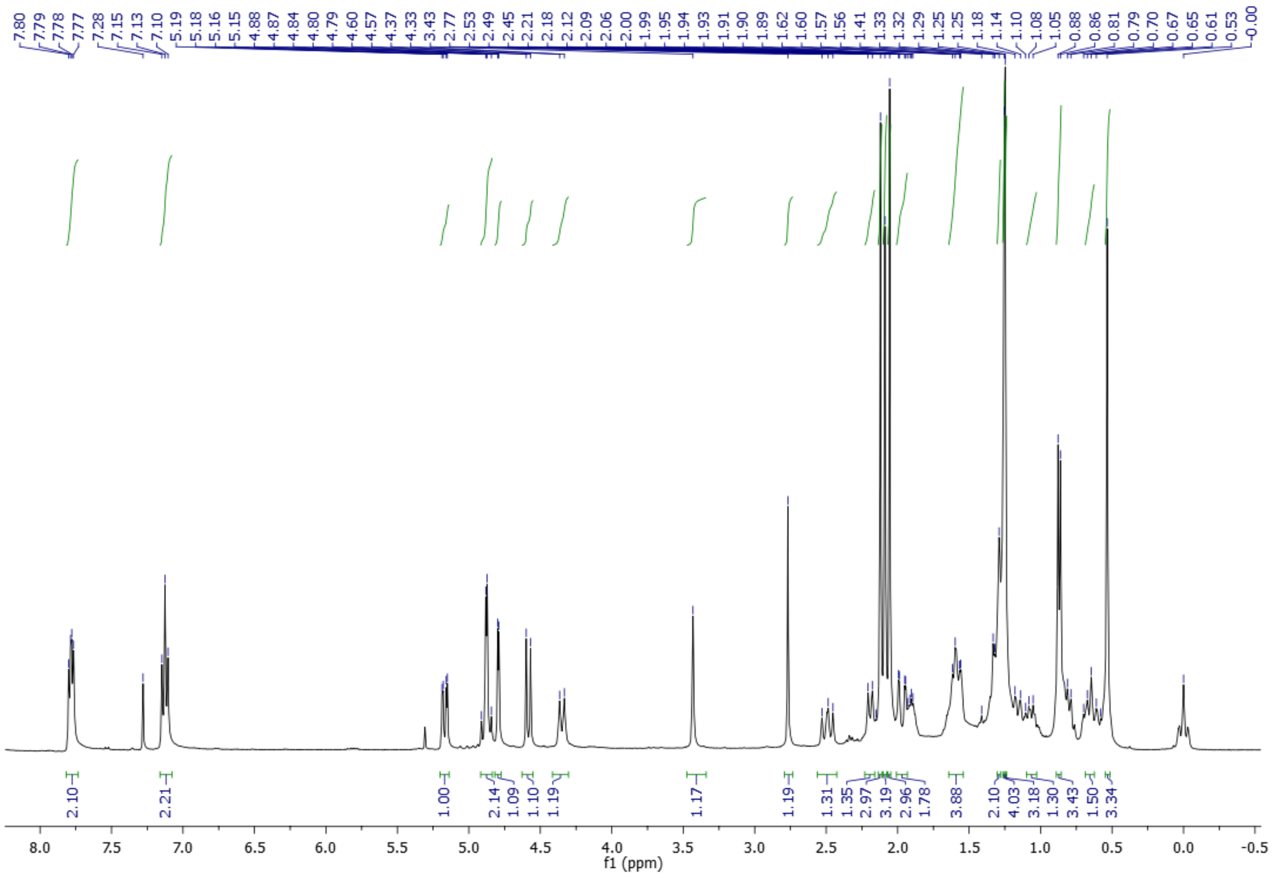

^{13}C NMR of W-12b

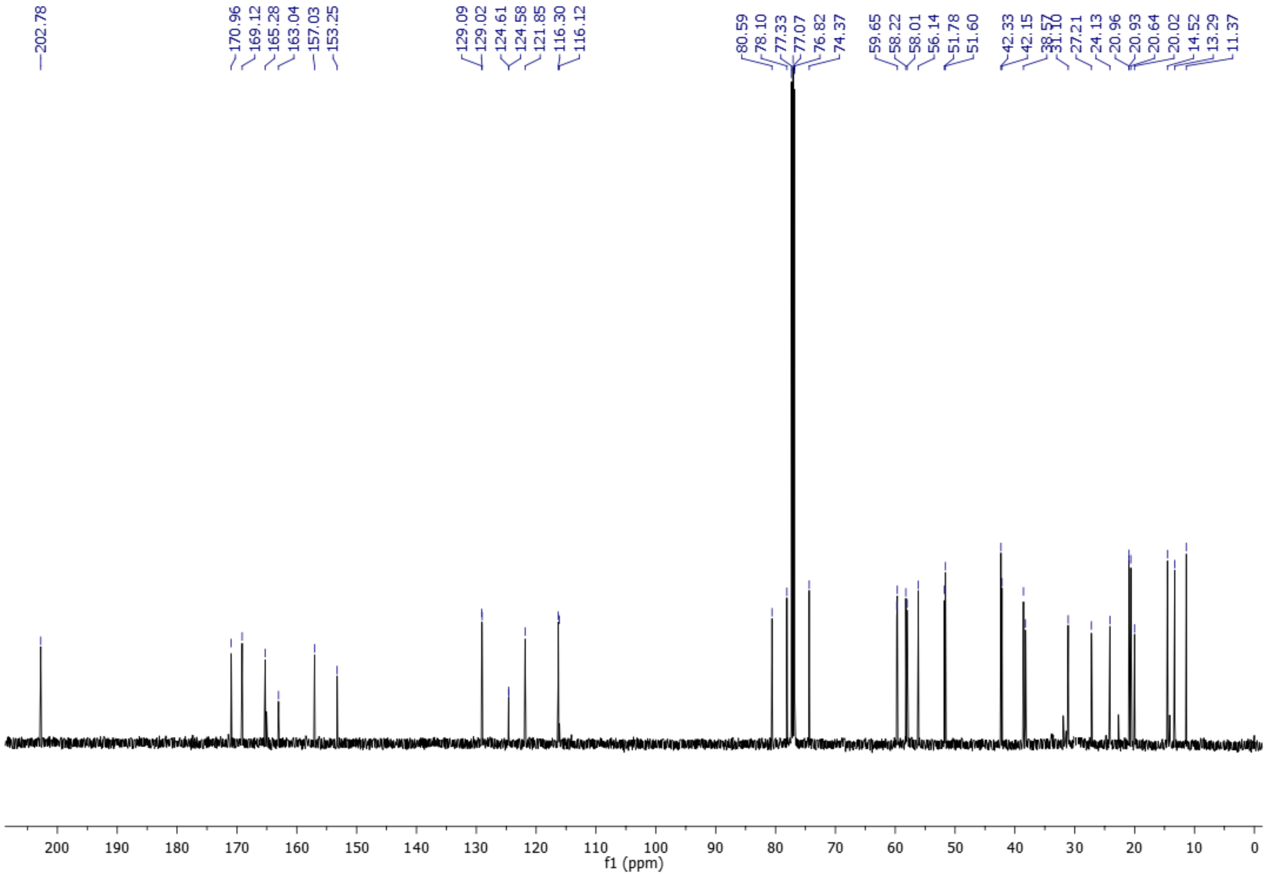

NOESY OF W-1a

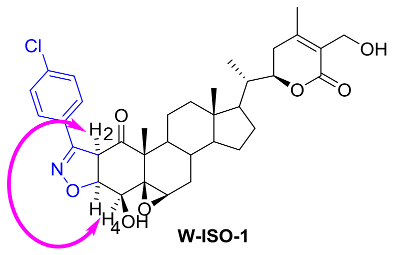

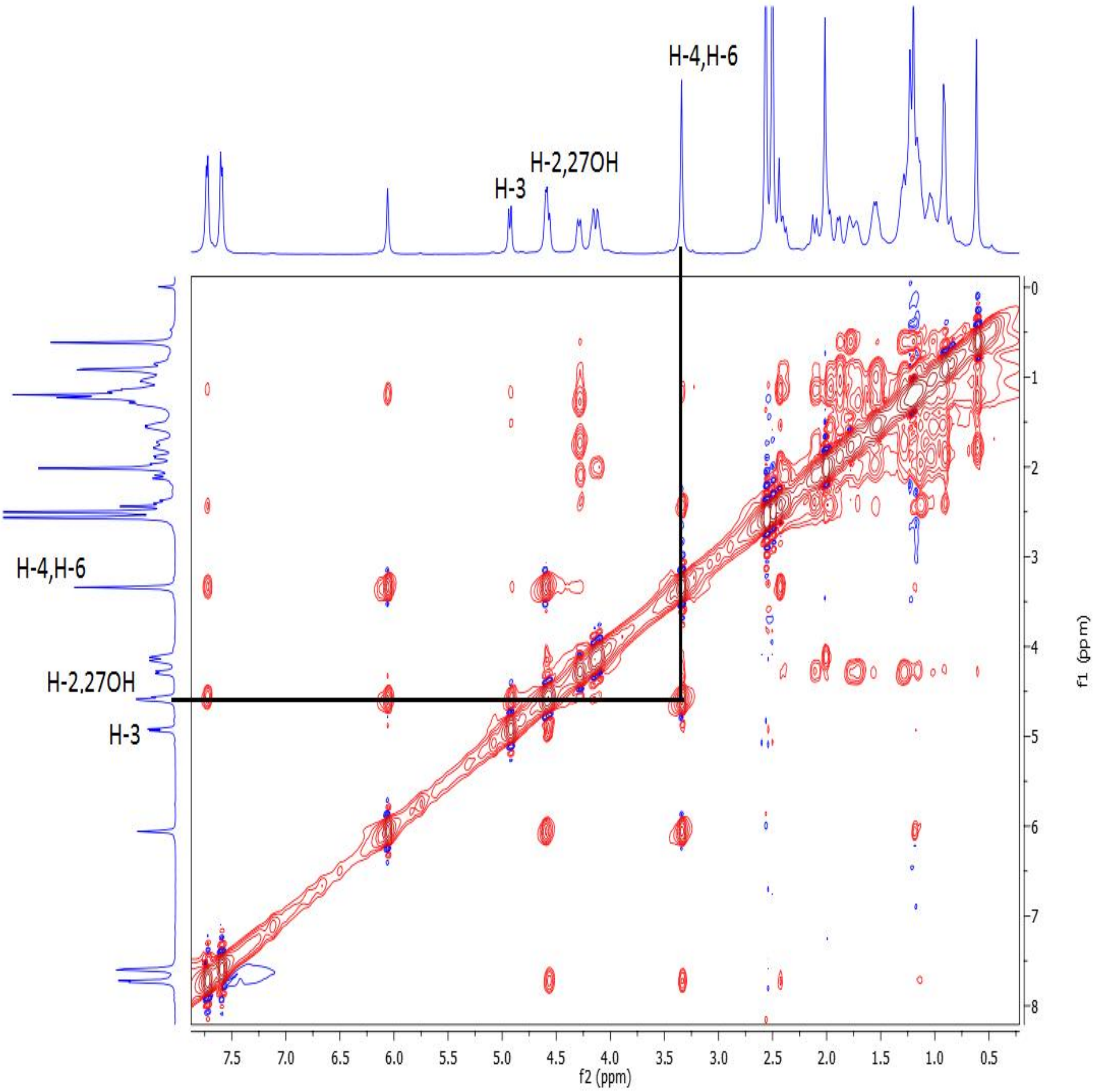

Cosy of W-1a

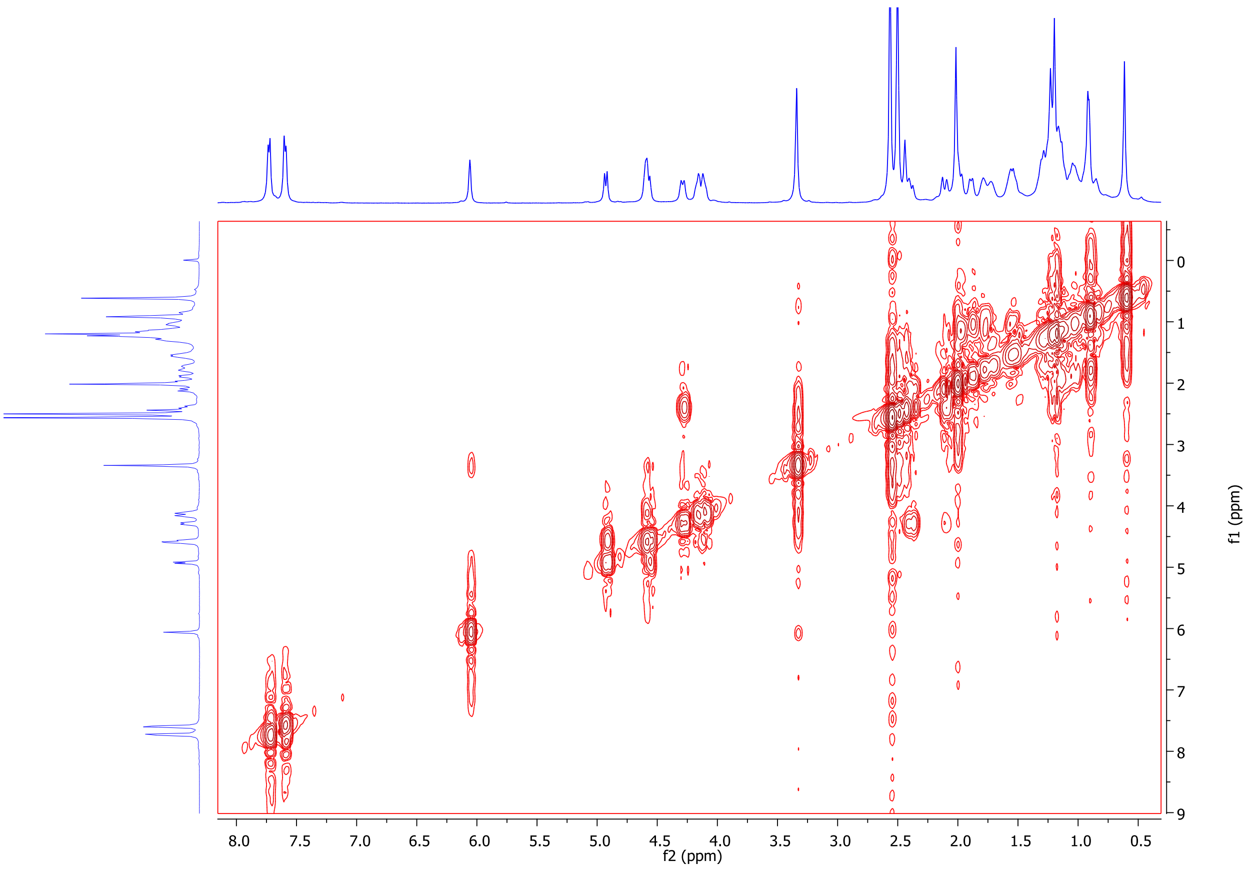

f2 (ppm)
f1 (ppm)

HMBC of W-1a

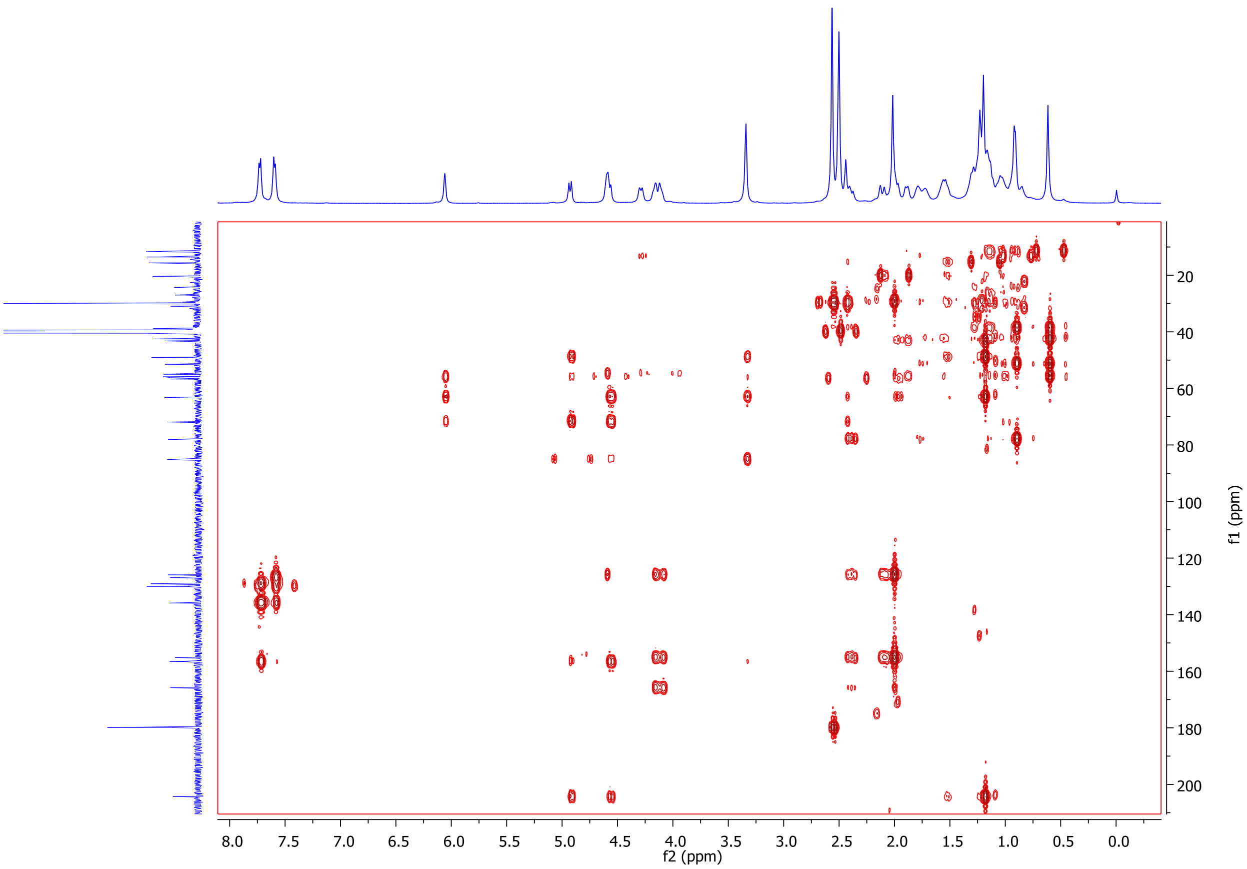

8.0
7.5
7.0
6.5
6.0
5.5
5.0
4.5
4.0
3.5
3.0
2.5
2.0
1.5
1.0
0.5
0.0
f2 (ppm)
20
40
60
80
100
120
140
160
180
200
f1 (ppm)

HSQC of W-1a

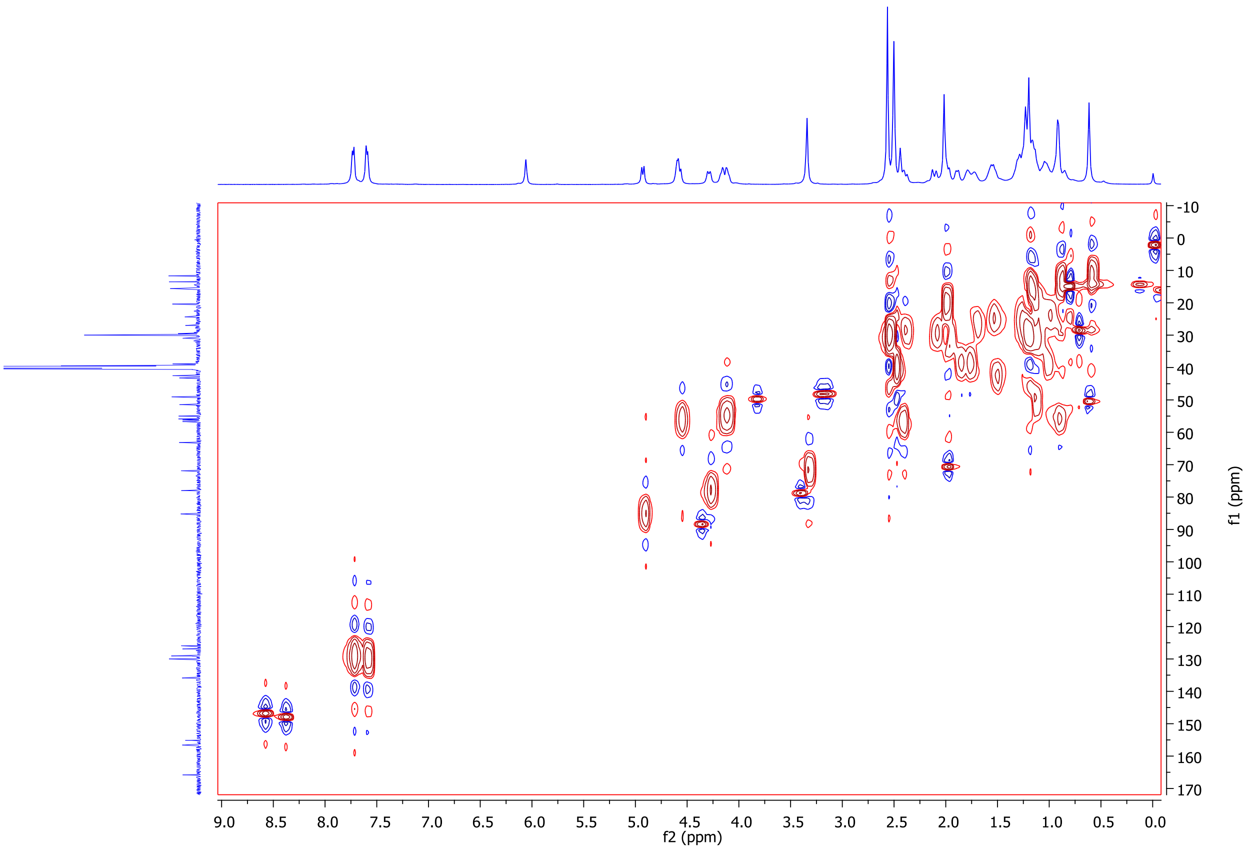

f2 (ppm)
f1 (ppm)
9.0 8.5 8.0 7.5 7.0 6.5 6.0 5.5 5.0 4.5 4.0 3.5 3.0 2.5 2.0 1.5 1.0 0.5 0.0
-10 0 10 20 30 40 50 60 70 80 90 100 110 120 130 140 150 160 170

NOESY OF W-1b

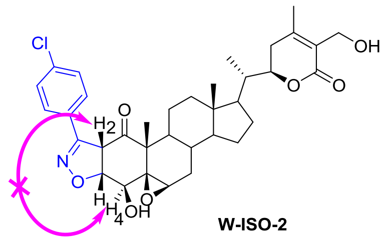

W-ISO-2

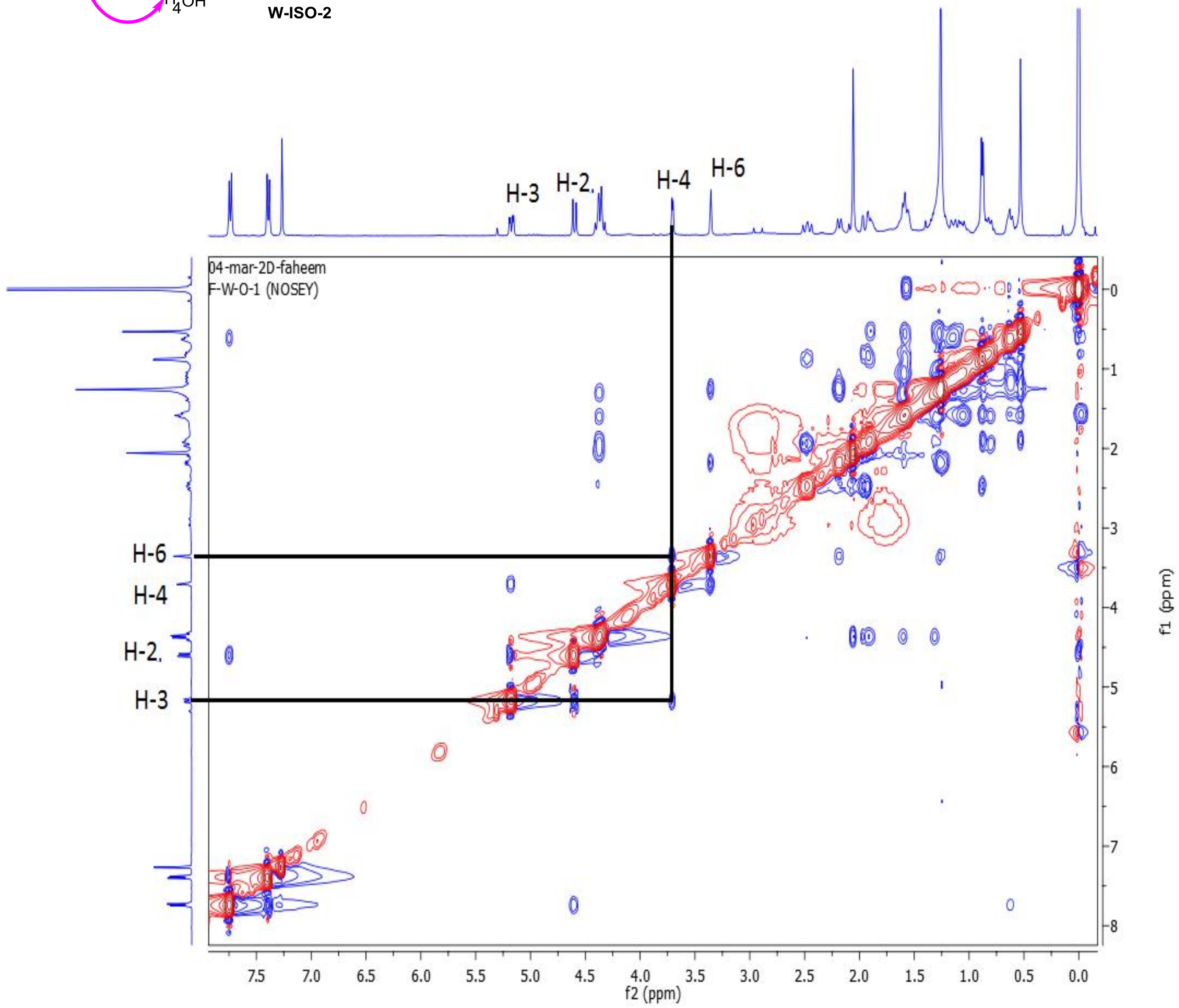

COSY of W-1b

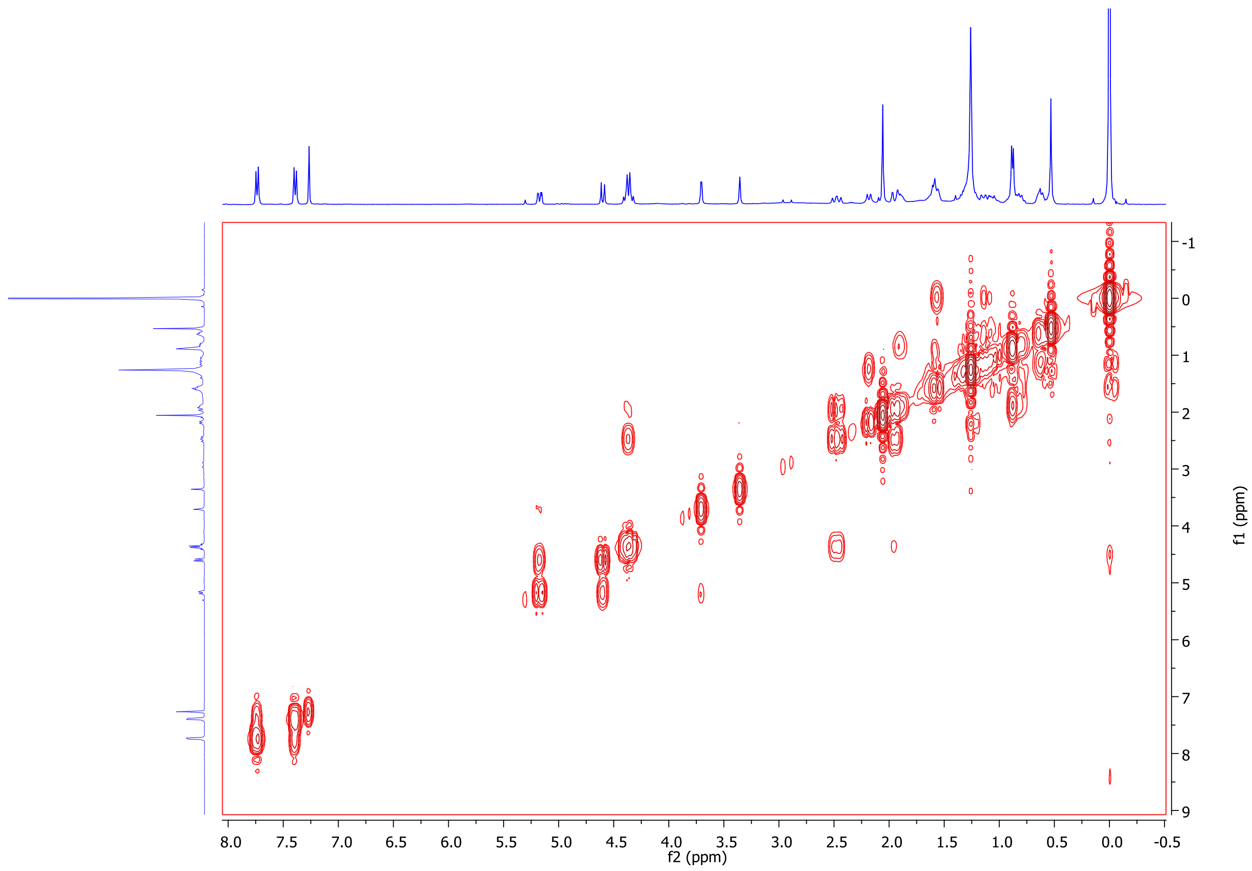

HSQC of W-1b

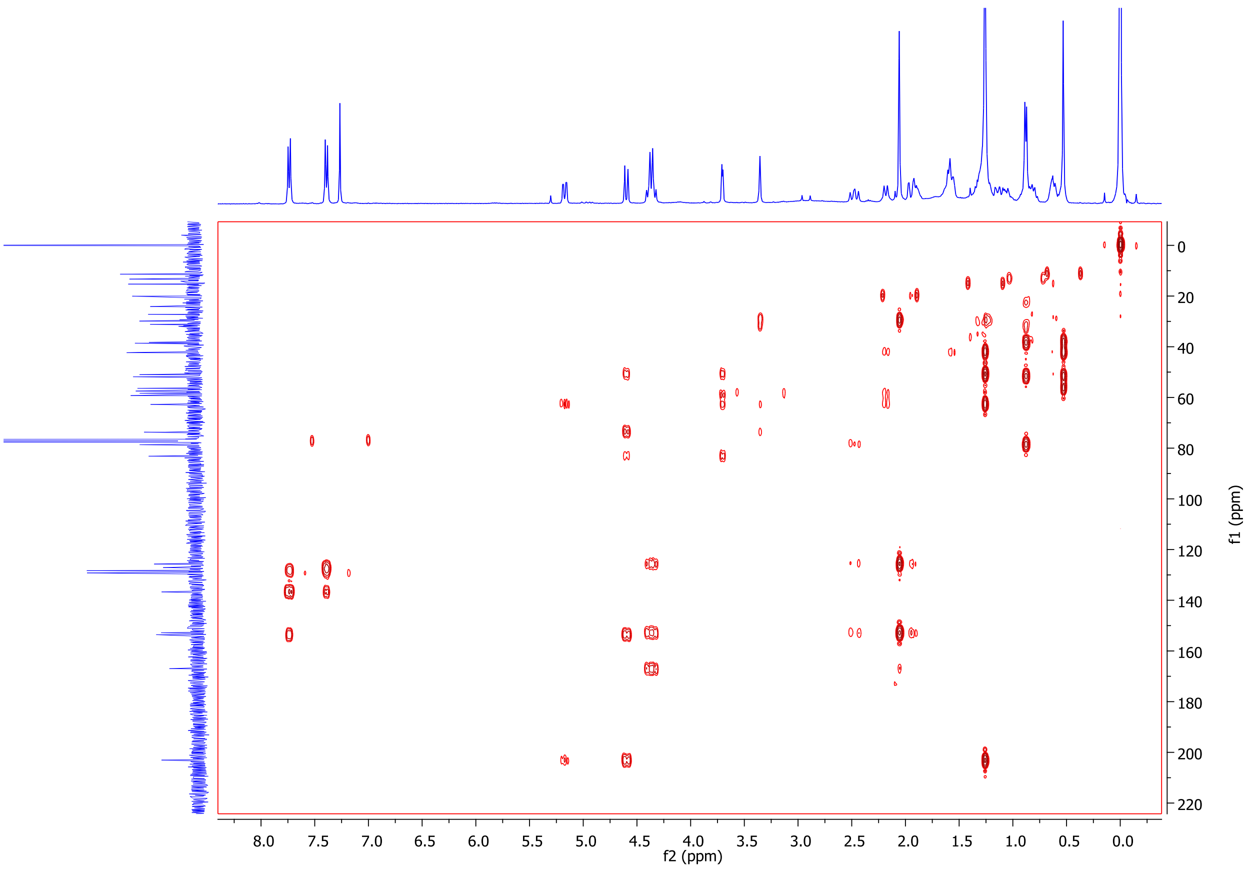

0
20
40
60
80
100
120
140
160
180
200
220
f1 (ppm)
8.0
7.5
7.0
6.5
6.0
5.5
5.0
4.5
4.0
3.5
3.0
2.5
2.0
1.5
1.0
0.5
0.0
f2 (ppm)

HMBC of W-1b

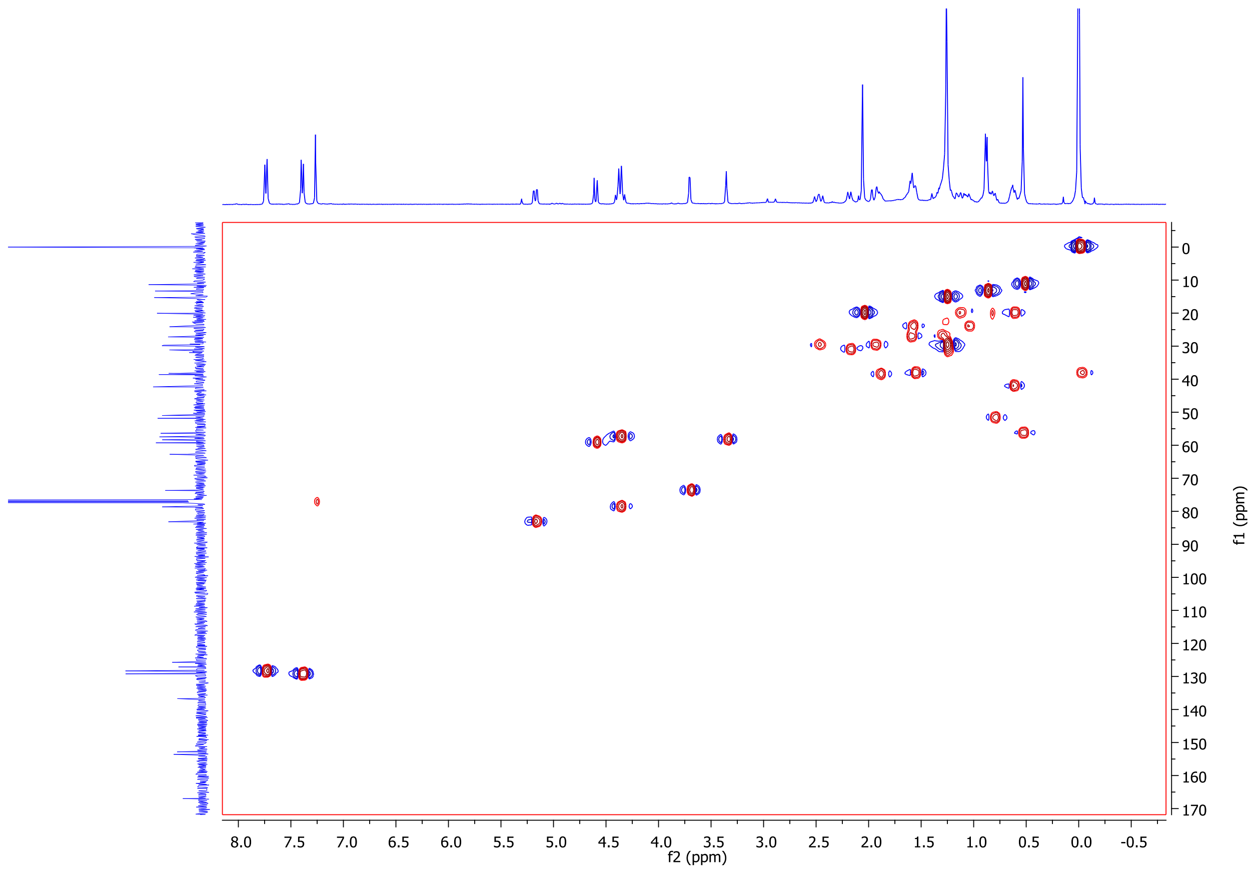

f2 (ppm)
f1 (ppm)
8.0 7.5 7.0 6.5 6.0 5.5 5.0 4.5 4.0 3.5 3.0 2.5 2.0 1.5 1.0 0.5 0.0 -0.5
0 10 20 30 40 50 60 70 80 90 100 110 120 130 140 150 160 170
